# Dampened sensory representations for expected input across the ventral visual stream

**DOI:** 10.1093/oons/kvac013

**Published:** 2022-08-15

**Authors:** David Richter, Micha Heilbron, Floris P de Lange

**Affiliations:** Donders Institute for Brain, Cognition and Behaviour, Radboud University Nijmegen, 6500 HB Nijmegen, The Netherlands; Donders Institute for Brain, Cognition and Behaviour, Radboud University Nijmegen, 6500 HB Nijmegen, The Netherlands; Max Planck Institute for Psycholinguistics, 6525 XD Nijmegen, The Netherlands; Donders Institute for Brain, Cognition and Behaviour, Radboud University Nijmegen, 6500 HB Nijmegen, The Netherlands

**Keywords:** dampening, sharpening, vision, expectation suppression, predictive processing, perception

## Abstract

Expectations, derived from previous experience, can help in making perception faster, more reliable and informative. A key neural signature of perceptual expectations is expectation suppression, an attenuated neural response to expected compared with unexpected stimuli. While expectation suppression has been reported using a variety of paradigms and recording methods, it remains unclear what neural modulation underlies this response attenuation. Sharpening models propose that neural populations tuned away from an expected stimulus are particularly suppressed by expectations, thereby resulting in an attenuated, but sharper population response. In contrast, dampening models suggest that neural populations tuned toward the expected stimulus are most suppressed, thus resulting in a dampened, less redundant population response. Empirical support is divided, with some studies favoring sharpening, while others support dampening. A key limitation of previous neuroimaging studies is the ability to draw inferences about neural-level modulations based on population (e.g. voxel) level signals. Indeed, recent simulations of repetition suppression showed that opposite neural modulations can lead to comparable population-level modulations. Forward models provide one solution to this inference limitation. Here, we used forward models to implement sharpening and dampening models, mapping neural modulations to voxel-level data. We show that a feature-specific gain modulation, suppressing neurons tuned toward the expected stimulus, best explains the empirical fMRI data. Thus, our results support the dampening account of expectation suppression, suggesting that expectations reduce redundancy in sensory cortex, and thereby promote updating of internal models on the basis of surprising information.

## INTRODUCTION

The perceptual system faces at least two challenges: to represent the world as quickly and accurately as possible and to promote processing of novel information. Relying on previous experience to guide perception may help to meet both challenges [1] and is advantageous to an agent acting in an information rich environment. Indeed, deriving expectations from previous experience aids performance, enabling faster and more accurate responses to expected events [2–5]. Within cortex, the consequences of prior expectations are evident during sensory processing in both early and higher visual areas [6]. One well-established neural consequence of prediction in perception is expectation suppression: the attenuation of sensory responses to expected compared with unexpected stimuli. Expectation suppression has been reported in several sensory modalities and species, using different recording methods, in a wide range of paradigms (for reviews, see [6, 7]).

However, it remains unclear what neural mechanism underlies this phenomenon. On the one hand, population sharpening models propose that expectations preferentially suppress neurons tuned away from the expected stimulus [8–10]. By inhibiting information that is inconsistent with top-down expectations, such a sharpening process would bias perception in line with our expectations, echoing Bayesian models of perception [11, 12]. The net result is a response that is reduced in amplitude, but carries a sharper, more reliable representation of the stimulus. On the other hand, dampening (or cancelation [13]) models argue that expectations preferentially suppress neurons tuned toward expected stimuli [14–18]. By canceling information in line with prior expectations, the brain would reduce redundancy in the sensory stream while at the same time favoring processing of novel or surprising information. Thus, on this account, responses are reduced and neural representations of expected stimuli dampened.

To date, studies that tried to arbitrate between these two accounts have yielded mixed results. In line with population sharpening, some studies [9, 10] found that expected stimuli evoked weaker BOLD responses but that stimulus identity was more accurately decoded from those same BOLD responses—suggesting a sharper representation. Moreover, it was found that expectation suppression was weaker in voxels that responded more strongly to the expected stimulus, in agreement with the hypothesized suppression of inconsistent information [9]. However, other studies using similar techniques found the opposite pattern of effects: reduced classification accuracies [15] or pattern similarities [14] for expected stimuli, and larger suppression magnitudes for preferred compared with non-preferred stimuli [17], in line with dampening accounts. One possible explanation for these inconsistencies is that the observed BOLD or MEG signal integrates over millions of neurons, making it difficult to infer neural-level mechanisms from population-level measurements. Indeed, in the domain of sensory adaptation, Alink *et al.* [19], building on work by Weiner *et al.* [20], recently showed that the relation between neural-level mechanisms and voxel-level results can be rather counter intuitive. Their simulations suggest that a dampening-like mechanism at the neural-level can, in principle, manifest as a sharpening-like result at the voxel-level, and vice versa. To overcome these interpretational difficulties, Alink *et al.* [19] proposed a forward modeling approach to explicitly model that underlying neural-level mechanism could best explain the observed voxel-level adaptation results. While adaptation and expectation are distinct phenomena [21–23], they share some key characteristics. This makes an analogous approach suitable to investigate expectation suppression.

Here, we build on and extend the approach of Alink *et al.* [19] and Weiner *et al.* [20] while addressing concerns raised by Ramírez and Merriam [24, 25]. In brief, we used forward models to elucidate the neural mechanism underlying expectation suppression in the ventral visual stream. First, we analyzed and integrated data of two previously published studies [17, 26], which manipulated perceptual expectations by presenting human volunteers (*n* = 56) with expected and unexpected objects images. For both studies, the effects of expectation were characterized in terms of seven fMRI outcome metrics, both univariate and multivariate. These metrics were based on previous studies, where they were interpreted as evidence for either sharpening [9, 10] or dampening accounts [14, 15, 17]. This resulted in a specific pattern of effects of expectation within three regions of interest (ROIs) across the ventral visual stream: primary visual cortex (V1), object selective lateral occipital complex (LOC) and temporal occipital fusiform cortex (TOFC). Next, we used forward models to explicitly model which neural mechanism best explained the observed effects in each ROI. We implemented a set of six distinct models, that all predict a suppression of neural responses to expected stimuli but differ in terms of the underlying mechanism of that suppression. In particular, we defined dampening as a local feature-specific gain modulation [15, 17], in which the gain of neural populations tuned toward the expected stimulus features is reduced. Conversely, we defined population sharpening as a remote feature-specific gain modulation [9, 10], in which the gain of neural populations tuned away from the expected stimulus features is reduced. Moreover, we modeled previously suggested feature-unspecific effects as a global gain modulation [17, 26]. As an additional competitor, we also implemented response tuning models, which narrow the width of the response function. These models have been suggested in the wider literature, as underlying response modulations for related phenomena like attention and adaptation [27–29].

To foreshadow the results, we show that perceptual expectations in the ventral visual stream are best modeled by a feature-specific local gain modulation of neural responses. Thus, our results are in line with dampening accounts of expectations, which advocate a suppression of neural responses particularly for neural populations tuned toward the expected stimulus features. This dampening of neural responses suggests that perceptual expectations, derived from statistical regularities, may reduce information redundancy and bias information processing toward surprising, novel information.

## RESULTS

In a first step, we analyzed the empirical fMRI data using seven outcome metrics used in previous studies investigating population sharpening and dampening [9, 10, 14, 15, 17, 19]. Next, using independent fMRI data, we validated the implemented stimulus feature spaces, which were used to model neural responses to each object stimulus in an ROI specific fashion. We then performed the simulation and analyzed the fit of the different models to the empirical results. Mimicking the interpretation in most empirical studies, we first assessed the qualitative fit in terms of the sign of the slopes per outcome metric. Additionally, we analyzed the model fit in a more detailed, quantitative fashion by calculating a weighted mean squared error (MSE) between the simulated and empirical results and assessed which model type best explained the empirical data. We then explored which parameter values resulted in the optimal fit, thereby exploring the circumstances under which the models best explain the observed data. Finally, we performed a control analysis illustrating that the here utilized forward modeling pipeline can recover known underlying neural modulations; see Text S1 and Fig. S1.

### Empirical fMRI data

First, we analyzed the empirical fMRI data. In brief, we utilized seven different outcome metrics, based on analyses used in previous studies (e.g. [9, 10, 14, 15, 17, 19]). (i) Mean amplitude modulation due to a stimulus being expected vs. unexpected, i.e. expectation suppression. (ii) Within-class correlation and (iii) between-class correlation between stimuli, for expected and unexpected occurrences of the stimuli. (iv) Classification performance defined as the difference between between-class minus within-class correlation for expected and unexpected stimuli respectively. (v) Amplitude modulation by amplitude, the amplitude modulation by expectation as a function of voxel mean amplitude. (vi) Amplitude modulation by selectivity, the amplitude modulation by expectation as function of voxel mean selectivity. (vii) Image preference analysis, assessing the mean amplitude modulation within a voxel as a function of image preference. Details are described in *Materials and Methods*, *fMRI outcome metrics*.

**Figure 1:**
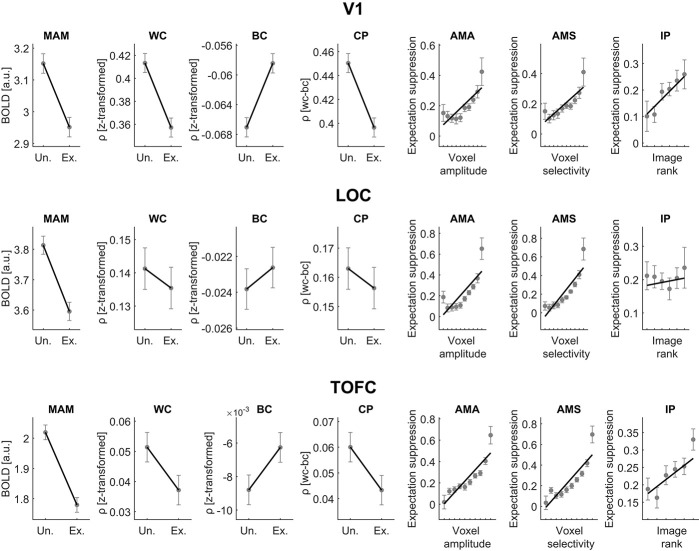
Empirical fMRI results. Shown are empirical fMRI data analysis results from V1, LOC and TOFC from a combined analysis of data from Richter and de Lange ([26]; *n* = 34) and Richter *et al.* ([17]; *n* = 22). These results constituted the data features the subsequent simulations were compared against. *Un.* indicates unexpected trials, i.e. when the trailing image was unexpected given the leading image. *Ex*. indicates expected trials, i.e. when the trailing image was expected given the leading image. Outcome metrics: *MAM* indicates mean amplitude modulation (expectation suppression); *WC*, within-class correlation; *BC*, between-class correlation; *CP*, classification performance (BC-WC); *AMA*, amplitude modulation by amplitude; *AMS*, amplitude modulation by selectivity; *IP*, image preference analysis. IP indexes the amplitude modulation by image preference rank and constitutes an addition to the set of outcome metrics assessed by Alink *et al.* [19]. The rationale of IP is to assess the magnitude of expectation suppression as a function of responsiveness within rather than across voxels. Positive IP slopes indicate that neural suppression increases the more responsive a neural population is to a given stimulus. For display purposes, only IP data of images 2–7 from Richter *et al.* [17] are displayed, but all image data were analyzed. Error bars denote within-subject SEM [30, 31].

Figure 1 depicts the fMRI results from the three ROIs, V1, LOC and TOFC. Data from Richter and de Lange [26] and Richter *et al.* [17] were combined by pooling participants. In all ROIs a substantial modulation of the mean amplitude was evident, with expected stimuli being suppressed relative to unexpected ones, i.e. constituting expectation suppression, the key phenomenon of interest. Furthermore, a difference in within-class and between-class correlation emerged between expected and unexpected stimuli, resulting in improved classification accuracies for unexpected stimuli in V1, while LOC and TOFC showed a similar albeit less reliable pattern. Moreover, voxels with larger mean amplitude and selectivity showed more expectation suppression (BOLD_unexpected_ – BOLD_expected_) in all ROIs. Within voxels we found larger suppression magnitudes for preferred compared with less preferred stimuli in V1 and TOFC. Since the goal of the analysis was only to estimate data features, which were subsequently used to compare the simulation against, we do not report inferential statistics here. However, for completeness, a full set of statistics, corresponding to the results displayed in Fig. 1, is summarized in Supporting Tables S1 (V1), S2 (LOC) and S3 (TOFC). Overall, empirical results were comparable between the three ROIs, with differences mainly emerging in terms of variability and effect sizes, while the sign of the effects (slopes) were the same.

### Feature space models explain neural variance in target ROIs

Because we modeled neural responses to different stimuli, we had to establish for each ROI a feature space model, which reliably describes the object stimuli in a manner relevant to the neural responses in the target ROIs (V1, LOC, TOFC). V1 feature space was defined by the predominant orientation of the object stimuli, as V1 neurons are tuned to stimulus orientation [32]. LOC responses were modeled by shape complexity, based on Vernon *et al.* [33]. TOFC feature space was derived from human-rated semantic similarity, which is thought to correlate with complex visual features [34]. Additional details are described in *Materials and Methods*, *Feature space*.

In a first validation step of the feature spaces, we compared stimulus arrangements between the three feature spaces using representation similarity analysis (RSA). Results demonstrated negligible correlations (Spearman’s Rho) between the arrangements (ρ_orientation & shape_ = 0.019, *P* = 0.416; ρ_orientation & semantic_ = −0.033, *P* = 0.701; ρ_shape & semantic_ = 0.002, *P* = 0.379), suggesting that each feature space captured largely distinct stimulus feature variance. A visual inspection of the object arrangements further supported that the three feature spaces successfully organized objects along the desired dimension; see Figs S5–S7. Thus, our three feature spaces resulted in distinct stimulus arrangements, a requirement for a meaningful differentiation of the stimuli for use in subsequent modeling.

**Figure 2:**
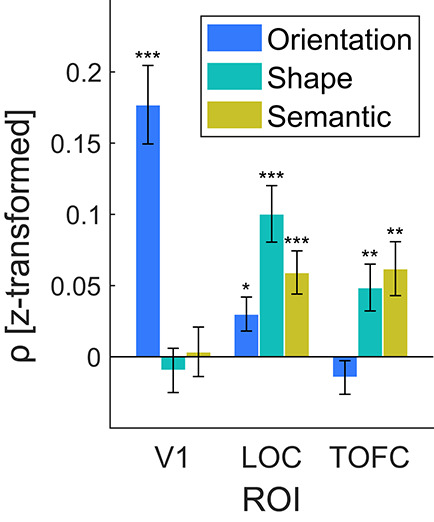
Stimulus feature space models explain neural variance in target ROIs. Shown are RSA results, in terms of fisher z-transformed Spearman’s rho (ρ), per ROI (V1, LOC, TOFC) and feature space model (orientation feature space, shape complexity feature space, semantic similarity feature space). In V1 only the orientation feature space explained significant neural variance. In LOC, all three models explained some neural variance; however, numerically the shape complexity model outperformed both other feature space models. In TOFC, the semantic similarity and shape complexity feature space models explained significant neural variance; however, the semantic similarity model explained numerically the most variance of neural responses. Thus, the designed feature spaces reliably captured neural variance in their target ROIs, validating the usefulness of the feature space models. Error bars indicate the SEM. ^*^ *P* < 0.05. ^**^ *P* < 0.01. ^***^ *P* < 0.001.

Next, to verify that each feature space captured significant variance in neural responses, we performed another RSA, using independent localizer data. In brief, for each participant, we correlated the feature space (model) representational dissimilarity matrix (RDM) with the neural RDM, obtained from an fMRI object localizer run, while controlling for the influence of the other model feature spaces using partial correlation. Subsequently, we compared the obtained correlation coefficients against zero (i.e. no correlation between feature space and neural RDM). RSA results, depicted in Fig. 2, showed that the feature spaces reliably explained unique neural variance in their target ROIs (V1, orientation feature space: *t*_(55)_ = 6.43, *P* = 3.3e-8, *d*_z_ = 0.86; LOC, shape feature space: *t*_(55)_ = 5.06, *P* = 5.1e-6, *d*_z_ = 0.68; TOFC, semantic feature space: *t*_(55)_ = 3.27, *P* = 0.002, *d*_z_ = 0.44). Detailed results of all associated tests are summarized in Tables S4 and S5. In sum, the designed feature spaces captured neural variance in their target ROIs, thereby validating the usefulness of these ROI specific feature space models.

### Simulation procedure

**Figure 3:**
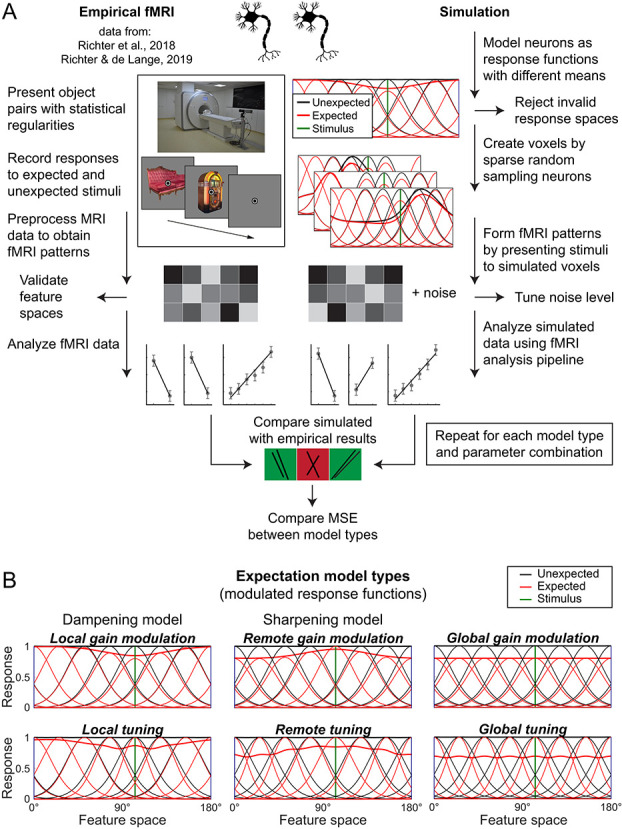
Simulation procedure and expectation models. (A) Overview of the empirical data acquisition and analysis, as well as the simulation procedure. On the left, the procedure for empirical fMRI data is depicted. Human volunteers were presented with object images, the identity of which was governed by statistical regularities, thereby making the objects expected or unexpected by virtue of the preceding image. The resulting fMRI data was analyzed using seven different outcome metrics (for details, see *Materials and Methods*, *Empirical fMRI data* and *MRI outcome metrics*). The right side illustrates the simulation procedure. Starting from the top, neural responses to different object stimuli were simulated in a ROI specific fashion, using empirically validated custom feature spaces (Fig. 2). Responses to expected stimuli were modulated using six different models. A fine-tuned amount of noise was added to the simulated response patterns, based on estimates of noise in the empirical data. Next, simulated data were analyzed using the analysis metrics also used for the empirical fMRI data analysis. Finally, simulated and empirical results were compared in terms of the sign of the slopes per outcome metric, as well as in a more fine-grained fashion by calculating a weighted MSE. For details, see Materials and Methods, Simulation. (B) Neural response functions and their modulation by expectation. Depicted are illustrations of the six neural response modulation models. Thin lines denote unmodulated (unexpected) and modulated (expected) response functions across feature space. Thick lines indicate the normalized summed response. The depicted example is from V1, thus representing a circular feature space. The vertical line shows the position of an example stimulus in feature space. Starting from the top left: local gain modulation (dampening) models reduced the amplitude of neural responses by a multiplicative factor (*a*); however, the magnitude of the response modulation depended on the distance between the expected stimulus and the response function (effect of distance is modulated by the *b* parameter). Remote gain modulation (sharpening) models were identical to local gain modulations, except that opposite neural populations, those tuned away from the expected stimulus, were modulated. Global gain modulations reduced the amplitude of the modulated response by a multiplicative factor (*a*) evenly across feature space. The tuning models (bottom row) reduced the width of the response function, with the magnitude of the reduction controlled by parameter *a*, thereby resulting in a more selective response. As with gain modulation models, the three distance functions apply. For details, see *Materials and Methods*, *Simulation*, *Modulation by expectations*. Note that the modulation by expectation was conditional on an expected stimulus being presented, thus modeling a top-down modulation. As such, the curves should not be seen as full tuning curves, but rather as an illustration how expectation differentially modulated different neural populations as a function of their tuning. See Text S2 and Fig. S2 for a discussion and illustration of alternative implementations.

An overview of the simulation procedure is depicted in Fig. 3A, and details are described in *Materials and Methods*, *Simulation*. In brief, we simulated neural responses to object stimuli using neural response functions, which modeled neural responses depending on the neuron’s response tuning and the properties of the stimulus in feature space.

Biased sampling [35] and macroscale maps [36] are two leading account of how stimulus selectivity arises in fMRI voxel data. We used a simple implementation in line with these accounts by random sampling a limited number of neurons with different feature tunings to form voxels [19]. As a consequence of the limited random sampling, simulated voxels showed distinct response preferences for different stimuli akin to the responses seen in empirical fMRI data. For more details, see *Materials and Methods*, *Simulation*, *Simulating voxels*. We then presented to these simulated voxels the same stimuli, on the same number of trials, as to the human volunteers during the fMRI experiments.

Neural responses for expected stimuli were modulated according to six distinct models, depicted in Fig. 3B. Two classes of modulations were employed. Gain modulations (‘scaling’ in Alink *et al.* [19]) linearly scaled the responsiveness of neurons, without modulating the shape of the response function. Tuning modulations (‘sharpening’ in Alink *et al.* [19]) narrowed the shape of the response function in feature space but did not affect the peak amplitude. Additionally, three distance functions were implemented, determining where in feature space, relative to the expected stimulus feature, the modulation was applied. For global models, modulations were applied equally across neural populations in feature space. In local models, neural populations tuned toward the expected feature value were modulated, while in remote models, neural populations tuned away from the expected feature value were modulated. Thus, the two models of particular interest were the local gain modulation model representing dampening (cancelation) accounts, and the remote gain modulation model implementing population sharpening accounts.

Simulated data were analyzed using the analysis pipeline as designed for the empirical data, relying on the seven outcome metrics described above. The entire procedure was repeated for each model type across a large parameter grid (*n* = 7820; for details, see *Materials and Methods*, *Simulation*, *Parameter grid*), extensively exploring the three free model parameters: *a* (suppression magnitude), *b* (effect of distance in feature space; only applicable to local and remote models) and *σ* (width of neural response function).

### Simulation results

#### Voxel-level results can be accounted for by opposite neural models

With the empirical fMRI results established, we performed the simulation. First, we analyzed results by comparing the sign of slopes per outcome metric between the empirical and simulated results, following the procedure from Alink *et al.* [19]. The rationale for this approach is that such qualitative interpretations of analysis results are used in most empirical fMRI and MEG studies. For example, improved classification accuracies of expected stimuli have been used as evidence for population sharpening [9, 10] and decreased accuracies for dampening [15]; indeed, similar qualitative interpretations apply for the other metrics as well, e.g. [14, 17].

**Figure 4:**
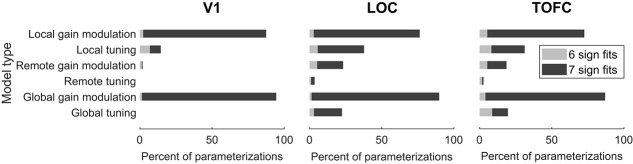
Qualitative assessment of model fits. Displayed are the percentages of parameterizations for each model type that fit all (seven sign fits; dark gray) or all but one (six sign fits; light gray) of the empirical fMRI outcomes. In higher visual areas, LOC (middle) and TOFC (right), all model types fit all seven outcome metrics under at least one parameterization. The percentage of good fits was noticeably larger for local and global gain modulations compared with all other model types. Similarly, in V1 (left), a larger proportion of parameter combinations showed a fit to the sign of slopes for local and global gain modulation models. However, in V1 remote and global tuning models failed to account for all (seven) or even all but one (six) empirical outcome metrics. Worse model parameterizations, with less than six outcome metric fits, are not displayed.

In V1, four of the six model types could, at least under one parameter combination, match the sign of the slopes of all seven outcome metrics found in the empirical data. Crucially, both models of interest, dampening (local gain modulation) and sharpening (remote gain modulations), could account for all outcome metrics. In LOC and TOFC, all model types succeeded in fitting the sign of all seven empirical outcome metrics. These results, depicted in Fig. 4, showed that no single voxel-level outcome or the combination of all seven outcome metrics was uniquely characteristic of sharpening or dampening, or any of the other implemented neural mechanisms. Thus, relying on a qualitative interpretation of only the sign of the slopes of the voxel-level results had only limited utility for the inference about underlying neural modulations. This conclusion was surprising and contrasts with the results reported by Alink *et al.* [19], who observed that only one model could qualitatively account for all outcome metrics with a single set of parameters. Therefore, they concluded that this model best explained the results overall. In our case, such qualitative reasoning alone did not suffice because all models in LOC and TOFC, and all but two models in V1, had at least one set of parameters that qualitatively matched all outcome metrics. This difference to Alink *et al.* [19] could be due to a finer and broader grid of parameters that was searched, using noise levels estimated from empirical data, and a more fine grained simulation of stimuli throughout the modeled feature-space (see *Discussion*).

While the present data showed that a qualitative analysis does not suffice to uniquely identify the best model, the results shown in Fig. 4 also demonstrated that the proportion of parameter combinations that fit all outcome metrics differed substantially between the six model types. In all three ROIs, local and global gain modulations showed the most robust fit across different parameter value combinations, fitting a maximal number of seven outcome metrics in V1 with 85% and 93% parameterizations, respectively, compared with 7% for the next best model type, local tuning. Similar results were evident in higher visual areas, with the sign of seven metrics successfully fit by local gain modulations in 73% (LOC) and 67% (TOFC) of parameterizations, and 88% (LOC) and 83% (TOFC) of global gain modulations. In contrast, local tuning did only account for all outcome metrics in 32% and 23% of parameter combinations in LOC and TOFC, respectively. In short, these results suggested that local and global gain modulations were less sensitive to the exact parameter values in producing the observed fMRI results. This robustness in turn increased the probability of these two model types reliably explaining the observed empirical results. That said, there were a substantial number of parameter combinations under which competing models did reliably produce the observed fMRI results as well, including remote gain modulation models. Moreover, a slope of the same sign did not necessarily accurately describe how well the simulated results fit the empirical results, as slope coefficients could differ drastically. Therefore, a quantitative approach was necessary to evaluate the model fit.

#### Perceptual expectations are best explained by a local gain modulation

Next, we quantitatively analyzed the fit of the simulated to the empirical results by calculating a weighted MSE for each model type and parameter combination. In brief, we compared the relative slope of the simulated and empirical results for each outcome metric. This difference in slopes was squared and the average per model type and parameter combination calculated. The average across outcome metrics was weighted by the inverse normalized standard deviation per outcome metric, thereby downweighing metrics with a high variance across simulated participants.

**Figure 5:**
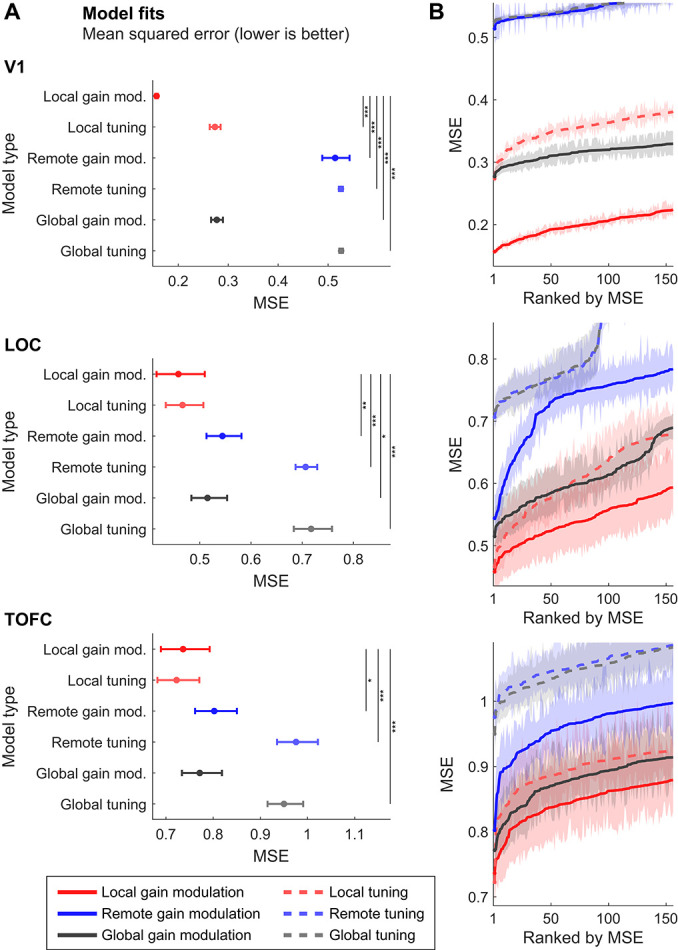
Local gain modulations best explain expectation suppression. Displayed are model fits in terms of MSE in the three ROIs (V1, LOC, TOFC) for all six model types. Local gain modulations represent dampening accounts and reduce the gain of neural populations tuned toward the expected stimulus features. Remote gain modulations represent sharpening accounts by suppressing the gain of neurons tuned away from expected stimulus features. (A) Depicted are the MSE of the best fitting parameterization for each model type. In V1 (upper panel) local gain modulation outperformed all competing models as evident by a significantly lower MSE than any other model. In LOC (middle panel) and TOFC (lower panel), local gain modulation performed better than almost all other model types and crucially, better than the remote gain modulation model. Unlike in V1, the local tuning model fit the data similarly well as the local gain modulation. Error bars indicate 95% bootstrapped confidence intervals, bootstrapped across modeled participants. (B) shows the model fit (MSE) across multiple parameterizations (top 2%; 156 parameterizations) for each model type, indicating the robustness of the model to changes in parameterization. In V1 (upper panel), local gain modulation outperformed all competing models as seen by a significantly lower MSE across all displayed parameterizations. Shaded error bars indicate 95% bootstrapped confidence intervals. In LOC (middle panel), local gain modulation reliably outperformed the remote models across all depicted parameterizations and all model types across most parameterizations. However, while numerically lower, some of the best model fits for local gain modulation did not significantly differ from local tuning models. Similarly, in TOFC (bottom panel), local gain modulation models significantly outperformed all remote models but did not provide reliably better fits compared with global gain modulation and local tuning models. In all ROIs the average performance of the local gain modulation models was reliably better than any competing model, as indexed by the mean MSE across the top 2% of models. In sum, local gain modulations, representing dampening models, performed significantly better than any other model type on all metrics in V1 and across most parametrizations in LOC. Results in TOFC were less clear, albeit a similar trend of numerical superiority of dampening models was evident as well.

Results depicted in Fig. 5 indicate how well each model type fit the empirical fMRI results. In particular, Fig. 5A shows that in V1, the best fitting model type (lowest MSE) was the local gain modulation (dampening) model. Indeed, all other model types had significantly worse fits to the empirical data. Similar results were evident, in LOC and TOFC, with almost all other model types, including the remote gain modulation (sharpening) model performing significantly worse than the local gain modulation. However, unlike in V1, in higher visual areas local tuning performed similar to local gain modulation, thus indicating that while the modulation was local, that is affecting neurons tuned toward the expected stimulus features, the precise modulation (gain modulation vs. tuning) was not distinguishable in terms of the best model fit alone.

Figure 5B provides additional information, depicting the fit of the best 2% of parameterizations (*n* = 156) for each model type, thereby indicating how reliable each model type could explain the empirical data. Results in V1 showed that all of the best parameterizations of local gain modulation performed significantly better than any competing model (for the distribution of best parameter values, see Supplemental Fig. S3 and Text S3). Thus, local gain modulation in V1 was robust to changes in the exact parameterization of the underlying model, suggesting that the superior performance of local gain modulation was stable and did not depend on exact parameter values, echoing the results shown in Fig. 4. In higher visual areas, LOC and TOFC results were similar albeit less reliable. Local gain modulations performed best across almost all parameterization and significantly outperformed any remote model across all top 2% of parameterizations. Thus, again local gain modulations performed reliably and robustly (across different parameterizations) better than the remote models, including the sharpening model. However, in LOC, local gain modulations only performed significantly better than all other model types across most, but not all parameterizations. In particular, local tuning and global gain modulation models showed numerically worse fits, but these were not reliably (statistically) different across multiple parameterizations from local gain modulations, probably due to the notably larger variance in model fits in LOC compared with V1—results were qualitatively similar in TOFC. Indeed, the model fit was overall better in V1 than in the higher visual ROIs. Nonetheless, on average, the MSE across the best parameterizations was significantly lower for local gain modulations compared with any other model type, indicating a more robust fit to the empirical data.

To ensure that successful models did not significantly violate known characteristics of neural responses, we constrained the forward models using biologically inspired criteria. In brief, we excluded models that (i) contained blind-spots for feature values, (ii) suppression profiles exceeding the average response difference of visual stimulation compared with baseline, (iii) a lack of feature tuning; for details, see *Materials and Methods*, *Simulation*, *Response requirements*. However, to demonstrate that the above presented results did not depend on this constraint, we repeated the simulation without enforcing these criteria in a control analysis. Results, depicted in Fig. S4, confirmed that local gain modulations best explained expectation suppression across all ROIs.

**Figure 6:**
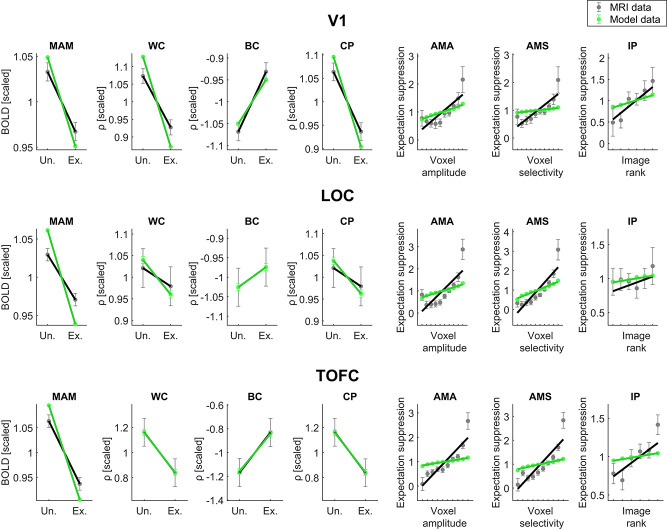
Fit of best models compared with empirical fMRI data. Depicted are the slopes and data for the best fitting forward model and fMRI data for the seven outcome metrics in each ROI. Data have been scaled (divided by the mean) for display purposes; for details on the forward modeling procedure, see *Martials and Methods*, *Simulation*. *Un.* indicates unexpected trials, i.e. when the trailing image was unexpected given the leading image. *Ex*. indicates expected trials, i.e. when the trailing image was expected given the leading image. Outcome metrics: *MAM* indicates mean amplitude modulation (expectation suppression); *WC*, within-class correlation; *BC*, between-class correlation; *CP*, classification performance (BC-WC); *AMA*, amplitude modulation by amplitude; *AMS*, amplitude modulation by selectivity; *IP*, image preference analysis.

Finally, Fig. 6 depicts the seven outcome metrics for both the empirical fMRI data and the best fitting model. Results confirmed that the sign of the modeled data matches the sign of the empirical outcome metrics. Moreover, for most metrics, the fit of the slope coefficients was evident by visual inspection. However, some divergences were also noticeable. First, mean amplitude modulation magnitudes were systematically overestimated by the best forward models. Second, the slope coefficients of the amplitude modulation by amplitude and amplitude modulation by selectivity metrics were underestimated. Furthermore, the fMRI data may have followed a quadratic trend on these metrics, while the model data showed a linear increase. These results suggest that there was more variability in suppression magnitude between voxels in the empirical data than the modeled data, constituting avenues for improvements in voxel formation for future modeling work. Combined, these results demonstrate that while the modeled data fit the sign and slope reasonably well, further improvements in model fitting exist, particularly for metrics reflecting complex, possibly non-linear relationships across voxels.

In sum, local gain modulations (dampening) models performed reliably better than any other model type in V1, both in terms of best model fit and across multiple parameterizations. In higher visual areas, local gain modulations performed better than any remote model, including the sharpening model, and reliably outperformed local tuning and global gain modulations across many, but not all parameterizations. In sum, our results favored local gain modulations, in line with dampening accounts, as underlying perceptual expectation suppression across early visual cortex (V1), and supported the dampening compared with the sharpening account in higher visual areas (LOC and TOFC).

## DISCUSSION

Predictions, based on statistical regularities in the sensory input, can be useful in guiding perception. In particular, predictions may aid to represent the world in a veridical fashion, as well as promote processing of novel and surprising information [1]. In the present study, we investigated the neural mechanism underlying a widely reported neural signature of perceptual predictions, expectation suppression: the attenuation of responses to expected compared with unexpected stimuli (for a review, see [6]). On the one hand, population sharpening suggests that expectations sharpen sensory representations in line with expectations, by suppressing neurons tuned away from the expected stimulus [8–10], modeled here as a remote gain modulation. On the other hand, the dampening (or cancelation) account proposes that expectations dampen sensory representations, by suppressing neurons tuned toward the expected stimulus [14–18], modeled as a local gain modulation.

We tried to arbitrate between population sharpening and dampening accounts of perceptual expectations by employing forward models and a large range of fMRI outcome metrics previously used by empirical studies investigating expectation suppression [9, 10, 14, 15, 17]. Our approach comprised two steps: first, we established the effects of expectations in terms of these outcome metrics for three ROIs, throughout the ventral visual stream, based on a large (*n* = 56) combined analysis of fMRI data from two prior studies [17, 26]. Next, we used forward models to quantitatively assess which underlying neural mechanism could best explain the observed effects of expectations. As neural mechanism, we modeled the two accounts of interest, sharpening and dampening, as well as alternative models based on the wider literature. In brief, we show that perceptual expectations in the ventral visual stream were best explained by a feature-specific local gain modulation, in line with dampening. These results suggest that expectations, as investigated here, selectively suppress neurons tuned toward expected stimulus features and may thereby serve to reduce information redundancy in sensory areas and highlight surprising and novel information.

### No qualitative pattern of fMRI results is unique to sharpening or dampening

First, we demonstrated that (almost) all model types, both gain modulation and tuning models, across all three distance domains, could fit the empirical fMRI results on all outcome metrics. The utilized fMRI outcome metrics have been employed by previous studies to arbitrate between different accounts underlying expectation suppression [9, 10, 14, 15, 17]. In V1, all model types, except for remote and global tuning, could, under at least one parameter combination, qualitatively fit all fMRI outcomes, i.e. the sign of the slope of the outcome metrics. In higher visual areas, LOC and TOFC, all model types could match the sign of all seven metrics. Dampening and sharpening accounts make opposite predictions in terms of the neuronal population that is most suppressed by expectations. Thus, it is counter-intuitive that opposite neural-level modulations can qualitatively fit the same voxel-level results on a broad range of analyses, even under biologically inspired constraints (see *Materials and Methods*, *Simulation*, *Response requirements*). These results highlight a crucial limitation of relying on heuristics in the interpretation of fMRI results, particularly if a limited number of fMRI analyses are utilized, echoing conclusions drawn by Alink *et al.* [19].

However, in contrast to Alink *et al.* [19], our results also show that relying on a purely qualitative interpretation of the results is not sufficient to reliably distinguish between population sharpening and dampening accounts, even if a combination of several fMRI outcome metrics is used. Indeed, sharpening and dampening models could qualitatively fit the empirical results in all three ROIs. This raises the question why the additional flexibility of the models in our simulation arose. First, it should be noted that our empirical fMRI results differ qualitatively from those presented by Alink *et al.* [19], particularly with an opposite sign of the slope of the between-class correlation metric. This difference in empirical data may relate to the different phenomena being studied, repetition suppression [19] compared with expectation suppression. It is possible that our pattern of fMRI results, especially the positive slope of the between-class correlation metric, is more readily fit by several model types. In addition, adjustments to the outcome metrics, particularly the correlation metrics, necessitated by the different fMRI paradigms (for details, see *Materials and Methods*, *fMRI outcome metrics*), may have allowed for more readily qualitative fits of the models. Another possible explanation for the additional flexibility of the models is that we modeled stimuli across the entire feature space, instead of only two specific feature values [19], hence resulting in a more dispersed and varied distribution of the stimuli across feature space. Moreover, we estimated noise levels, using independent empirical data, instead of adding a small, fixed amount of noise. A potential influence of noise on the utilized forward models was pointed out by Ramírez and Merriam [24], although follow-up analyses by Alink *et al.* [25] showed that their conclusions were (for their ‘face’ dataset, which most resembles our data) robust to variations in noise levels. However, the analysis by Alink *et al.* [25] also demonstrated that not all noise levels [specifically, signal-to-noise ratios (SNRs)] resulted in the same overall fits, thus rending differences in noise modeling a possible explanation for the discrepancy. Finally, a substantially finer resolution and broader scope of the explored parameter grid may have contributed to additional model flexibility as well, even though we also enforced additional response constraints based on plausible neural responses (see *Materials and Methods*, *Simulation*, *Response requirements*). In sum, the resulting versatility necessitated a more fine-grained, quantitative analysis of the model fits.

### Perceptual expectations dampen sensory representations

In early visual cortex (V1), local gain modulation models, representing the dampening account, performed significantly better than all other implemented models. In fact, not just the best performing model (smallest MSE) was local gain modulation, but this superior fit to the empirical data was stable and robust to variations in the precise parameter values of the model, i.e. all top 2% (*n* = 156) of local gain modulation models fit the empirical data better than any other model. Similar, albeit less clear, results were seen in higher visual areas LOC and TOFC. Crucially, the dampening model (local gain modulation) performed reliably and robustly better than the sharpening model (remote gain modulation), both in terms of the best model fit and across all of the top 2% of model parameterizations. However, unlike in V1, local gain modulations did not outperform local tuning models in terms of the best fitting model, and only fit the data better for most of the best 2% of parameterizations in LOC. Nonetheless, local gain modulations did show an overall more reliable fit to the empirical data as indicated by a superior fit compared with all other model types across multiple parameterizations, as well as on average across the best model fits.

In sum, our results, across early, intermediate and higher visual areas support the dampening account compared with the sharpening account. Indeed, overall local gain modulations appear to be the preferred model in fitting the present data, thereby converging on a similar neural mechanism as underlying expectation suppression as previously proposed based on electrophysiological recordings in non-human primates [16, 18]. Interestingly, previous work also shows that local gain modulations underlie stimulus adaptation as well [19], thus suggesting comparable neural modulation accounting for both phenomena, adaptation and expectation suppression. Similar neural modulation should, however, not be mistaken as an identity of the two phenomena [21–23].

Having established that local gain modulations likely underlie expectation suppression in the ventral visual stream, it is worth considering what functional role expectations may have in perception according to the dampening account. The hallmark of dampening (here modeled as local gain modulation) is a suppression of responses in neural populations tuned toward the expected stimulus features [14, 15, 17, 18]. By suppressing neurons tuned toward the expected stimuli, a dampening of neural responses reduces redundancy in the sensory system. That is, if a stimulus was well predicted by internal models, there is no need to vigorously respond to that stimulus, as it presents little new information. Indeed, information is particularly relevant to an agent, in so far as it is novel information, because such information is valuable for updating internal models of the world, which in turn can promote adaptive behavior. Moreover, suppressing uninformative, well-predicted input may additionally preserve processing and attentional resources. Therefore, it seems adaptive that expectations can guide perception by suppressing expected input and highlighting unexpected stimuli, i.e. events that are informative.

### Reconciling sharpening and dampening accounts

As discussed above, the present results support the dampening account of expectation suppression, with expectations highlighting novel information, and deemphasizing expected, predictable input. These results may appear incompatible with the competing population sharpening account, which, in line with Bayesian views of perception [11, 12], suggests that predictions sharpen representations in line with expectations. Indeed, our implementation of sharpening, remote gain modulation, performed poorly in all three ROIs. Moreover, our results show that, when assessed qualitatively, no single fMRI outcome metric can reliably distinguish between sharpening and dampening models. Hence, it is possible that previous results in favor of sharpening at the population level may be explained by local gain modulations. Further work is required to assess this possibility, such as by modeling neural responses in datasets previously cited in support of population sharpening.

However, there are other attempts to reconcile the two seemingly incompatible accounts. For instance, hierarchical predictive coding theory proposes that sharpening and dampening occur in parallel but in different neural populations—namely, prediction and error neurons, which would reside in superficial and deep layers of cortex, respectively [37]. Note, however, that so far there has been no direct evidence for the existence of these two neuron types and that this proposal does not explain why the present results would only reflect the dampening process occurring in the error neurons.

Alternatively, Press *et al.* [1] recently proposed that both processes, population sharpening and dampening, operate during different processing stages. The rationale is that initial processing relies on prior knowledge to sharpen sensory representations, followed by a late processing stage, dampening neural representations of the expected stimulus. Thereby, this account promises to unify results in the literature that initially appear incompatible (e.g. [8–10] vs. [14–18]). At the same time, this proposal also acknowledges the adaptive value of predictions in fulfilling both challenges facing perception, veridical representations aided by prior knowledge, as well as using prior knowledge to reduce redundancy and help in information seeking and updating of internal models.

That said, the present results only provide evidence in line with the hypothesized late dampening stage. However, given that in the analyzed fMRI datasets (for details, see [17, 26]) object stimuli were presented at full contrast, without visual noise, and for a fairly long duration (500 ms), it is conceivable that in this context a representational sharpening stage is of little relevance for veridical perception and good task performance. Consequently, the sharpening processing stage may have had little impact on the overall BOLD signal, which represents (indirectly) the integrated neural response over an extended time period. Thus, in the present data, the later representational dampening stage may have dominated the observed BOLD signal. On the other hand, in Kok *et al.* [9], participants performed a more perceptually demanding discrimination task, which may have placed emphasis on the sharpening stage, thereby resulting in the observed suppression of neural populations tuned away from the expected stimulus.

In sum, more work is required to assess whether distinct neural modulations do in fact underlie reports of population sharpening compared with dampening, or if local gain modulations account for both population level phenomena. If different modulations do in fact underlie population sharpening, the two stage processing account could be tested by utilizing more time-resolved methods. Moreover, it may proof useful to assess whether the dominance of one or the other process can be tipped by changing task demands and stimulus characteristics, e.g. perceptually challenging paradigms resulting in a sharpening of representations, overruling the dampening effect in the BOLD signal.

### Limitations

The present results must be interpreted with some limitations in mind. First, while we synthesized empirical fMRI data from two separate studies, resulting in a large dataset (*n* = 56) including separate tasks, it is crucial to consider the type of perceptual expectations investigated in these datasets. Both studies probed visual expectations extracted incidentally from statistical regularities. Thus, it remains unclear whether the perceptual expectations studied here, following incidental statistical learning, involve similar neural mechanisms and consequences as explicitly learned expectations. It is possible that different routes toward the acquisition of statistical regularities exist, relying on different neural mechanisms [38], thus raising the question whether the resulting sensory consequences may also differ. Relatedly, both datasets involved full-color object images as stimuli. While studies using various kinds of stimuli have provided support for the dampening account [14–18], it remains unclear whether specific stimulus types may result in distinct neural modulations, as at least one study in support of sharpening utilized oriented gratings [9]. In sum, at present, it is unknown whether specific task requirements, learning or stimulus types could induce different prediction-based neural modulations.

#### Oversimplification in the model setup

The implemented forward models necessarily involve an oversimplification of the neural mechanisms and responses in visual cortex. For example, we relied on limited random sampling of neurons to form voxels with different response profiles, mirroring the large scale response preferences evident in empirical fMRI data. While our approach is in line with biased sampling [35] and macroscale maps [36], two leading accounts of voxel selectivity, there certainly are more refined and biologically plausible implementations. Additionally, we chose to bypass the complex dynamics involved in the hemodynamics underlying the BOLD signal, which constitutes a significant oversimplification. Moreover, some alternative theories of stimulus selectivity may not be in agreement with our model (e.g. stimulus vignetting [39]), and thus our results cannot speak for such mechanisms. That said, the primary assumptions of our implementation appear fairly robust, a monotonic relationship between neural activity and voxel responses, and that voxel-level response preferences indirectly reflect neural tunings. Therefore, even though our model constitutes an oversimplification of the associated neural and hemodynamic processes, if these two core assumptions hold, our results are nonetheless likely to be informative about how expectations modulate neural responses. Moreover, in a control analysis, detailed in Text S1 and results depicted in Fig. S1, we demonstrate that the here utilized forward modeling approach can recover known underlying models from simulated fMRI data, thereby supporting the reliability of the results.

#### Limitations of the fitting procedure

In addition to the above outlined limitations, we acknowledge that the here utilized procedure for determining the best fitting model has inherent limitations. For example, it is known that noise across voxels in fMRI data is spatially correlated. Such spatial correlations are not accounted for in our present modeling procedure, thus constituting a divergence between the forward models and the fMRI data, likely impacting the fit of multivariate outcome metrics (e.g. the classification performance). Future work could improve on this aspect by explicitly including spatially correlated noise, informed by empirical fMRI data. Moreover, more advanced methods for model fitting, such as iterative model fitting instead of a grid search, could be used in future work to further improve the here used method. However, we also highlight that using a systematic grid search allows us to further assess the influence of the parameter values on the model fit—for details, see Supplemental Figs S8–S10.

#### Surprise enhancement or expectation suppression?

We chose to model expectation suppression as a net suppression of neural responses to expected stimuli. However, the fMRI data can also be explained by an enhanced response to unexpected stimuli, surprise enhancement. Defining a neutral comparison condition to tease apart expectation suppression and surprise enhancement is challenging, both conceptually and experimentally. Our choice to model a net suppression of neural responses is based on predictive coding theories, which suggest that expectations explain away neural activity [37, 40]. That said, inverting the modulation in our model (i.e. modeling surprise enhancement) would merely invert the remote and local models. Thus, to account for the local gain suppression found here, an opposite remote gain enhancement would result in a comparable modulation, if surprise enhancement were modeled. Therefore, our conclusion that expectations dampen neural representation relative to unexpected stimuli and that this dampening is achieved by a relative suppression of neurons tuned toward the expected stimuli, compared with the response to an unexpected stimulus, would remain unchanged. In other words, in our modeling setup, expectation suppression and surprise enhancement are different ways of describing the same effect. Note that this may also apply to the phenomenon more broadly: if response amplitude to a stimulus is inversely proportional to stimulus (log) probability—as theories of predictive processing propose [37, 41]—expectation suppression and surprise enhancement are two sides of the same coin.

#### Does pair coding account for expectation suppression?

Another alternative explanation for our results worth ruling out is that expectations may not modulate neural responses to the predicted stimuli, but that instead, expected image pairs become associated over repeated exposures, and subsequently the presentation of the first stimulus activates the expected pair as a union. On this pair-coding account, the representations of paired (expected) stimuli would be activated more frequently than of non-paired (unexpected) stimuli, thereby accounting for expectation suppression by pair repetition frequency. However, if associated stimuli become paired over time, we would also expect that their neural representations become more similar. We ruled out this explanation in a supplementary analysis (for details, see Text S4) by showing that paired stimuli were not represented in a more similar fashion than non-paired stimuli, thereby suggesting that pair-coding is unlikely to account for expectation suppression in our data.

#### Feature space limitations

Other limitations worth considering are the utilized feature space models. There is ample room to improve the feature space definitions with more complex implementations, and thereby increase the amount of explained neural variance. This may in turn help to more clearly distinguishing between the different predictions of the neural mechanism underlying expectation suppression. That said, we did show that even our simple feature spaces reliably capture neural response variance in their target ROIs and are sufficient to yield distinguishable characteristics between population sharpening and dampening accounts. Moreover, we further validated our feature space by showing that each feature space indexed unique variance of the stimulus characteristics (for details, see *Materials and Methods*, *Feature space comparison*).

#### Converging on similar conclusions using multiple methods

By casting a broad parameter grid and assessing results according to a variety of parameter combinations, instead of only the best fitting parameterization per model, we demonstrate the stability and robustness of our results to noise and changes in parameter tuning. This in turn also increases the robustness of the drawn conclusions.

Finally, the present results should not be seen in isolation. Using a novel approach in the investigation of expectation suppression, moving from neural-level models to voxel-level data, we converge on a similar conclusion as previous studies using a variety of other analyses and recording methods, including fMRI [14, 17], MEG [15], as well as single [18] and multi-unit recordings [16]. Combined, these results provide robust evidence in favor of the dampening account.

## CONCLUSION

In sum, we show that across the ventral visual stream, perceptual expectations, following statistical learning of associations between object images, result in a feature-specific suppression of the gain of neural responses. This feature-specific suppression is particularly affecting neurons tuned toward the expected stimulus features. As a result, this suppression dampens neural representations of expected stimuli, thereby potentially reducing redundancy in sensory cortex and emphasizing processing of surprising, novel information. Moreover, whether the here supported dampening can operate in concert with a sharpening of representations during different stages of visual processing [1] poses an intriguing avenue for future research.

## MATERIALS AND METHODS

### Empirical fMRI data

This section briefly describes the experimental protocol of the two fMRI datasets; for a full description, see Richter and de Lange [26] and Richter *et al.* [17].

#### Experimental paradigm

**Figure 7:**
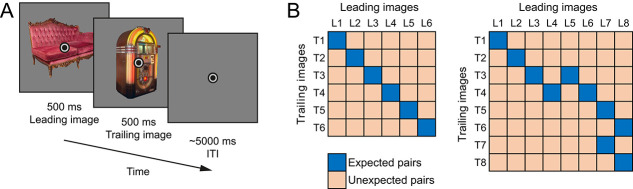
Experimental paradigm and image transition matrix. (A) Depicts a single trial. A leading image (500 ms) is followed by a trailing image (500 ms), the identity of which is (un-)expected given the leading image. The trials end with an ITI of ~5000 ms. (B) shows the image transition matrices determining the association between images. Expected image pairs are denoted as darker cells. Each trailing image occurs as expected and unexpected image, with expectation status depending only on the leading image on a given trial. The transition matrix on the left is from the experiment reported in Richter and de Lange [26], while the matrix on the right is from Richter *et al.* [17].

In both experiments, participants (*n* = 34 and *n* = 22 after data exclusion) were presented with two full-color object images in quick succession. Each image was presented for 500 ms, without interstimulus interval, and an intertrial interval of ~5 seconds; Fig. 7A depicts a single trial. Crucially, the identity of the trailing (second) image was predictable given the identity of the leading (first) image. Thus, each trailing image could either be expected or unexpected given the leading image. In Richter and de Lange [26], the transition matrix during a learning session consisted of 12 leading and 12 trailing images with deterministic associations (i.e. only expected pairs) on a total of 960 trials. During fMRI scanning, a subset of 6 × 6 images was shown using probabilistic associations (50% reliability, i.e. the expected image was five times more likely than any unexpected image; Fig. 7B, left panel) on a total of 240 trials. Transitions were task-irrelevant during the learning session (unpredictable oddball detection) but could aid task performance during fMRI scanning (classification of trailing images). In Richter *et al.* [17], eight leading and eight trailing images were shown (Fig. 7B, right panel). During the learning session (2012 trials, 100% reliability) as well as during fMRI scanning (512 trials, ~56% reliability) statistical learning was incidental, i.e. the statistical regularities were not related to, or helpful in performing the task (unpredictable oddball detection). Only non-oddball trials (456 trials), without any behavioral responses, were analyzed. Additionally, both studies had one localizer run during which stimuli were presented in an expectation neural context for ~12 seconds, one at a time, flashing at 2 Hz.

#### fMRI data acquisition

Data were acquired on a 3T Prisma [26] and 3T Skyra [17] scanner, using 32-channel head coils. In both cases, a whole-brain T2*-weighted multiband sequence was used to acquire functional MRI data. Data from Richter and de Lange [26] were acquired using a multiband 6 sequence with 2-mm isotropic voxel size, and data from Richter *et al.* [17] with a multiband 8 sequence with 2.4-mm isotropic voxel size. T1-weighted images were acquired in both studies using a magnetization prepared rapid gradient echo sequence (MP-RAGE) sequence with 1-mm voxel size.

#### fMRI data preprocessing

Preprocessing of the empirical fMRI data was performed using FSL 6.0 (FMRIB Software Library; Oxford, UK; www.fmrib.ox.ac.uk/fsl; [42]; RRID:SCR_002823). The preprocessing pipeline included: brain extraction (BET), motion correction (MCFLIRT) and temporal high-pass filtering (128 s). No spatial smoothing was applied, as voxel patterns were of primary interest. Functional images were aligned to the middle volume of the localizer run. All analyses were performed in native space to avoid unnecessary data interpolation.

#### fMRI data preparation

The preprocessed fMRI data was further analyzed using the least squares separate approach by Mumford *et al.* [43] and Turner *et al.* [44]. A separate GLM was fit for each trial, consisting of one regressor of interest, modeling the response to the stimuli on the current trial. Additionally, regressors of no interest were added, consisting of a regressor per trailing image type (excluding the current trial), one regressor modeling events of no interest (instruction events) and 24 motion regressors (FSL’s standard + extended set of motion parameters). Regressors were convolved with a standard double-gamma HRF. Finally, the parameter estimates for each trial and ROI were extracted separately, which constitute the pattern of responses to the stimuli presented on each trial.

#### ROI masks

The same ROI masks were used as described in Richter and de Lange [26] and Richter *et al.* [17]. In brief, three ROIs were defined *a priori*: primary visual cortex (V1), object selective LOC, and TOFC (akin to inferior temporal cortex). All three ROIs were defined anatomically and functionally. Moreover, ROI masks were further constraint to the 200 (data from [17]) or 300 (data from [26]) most informative voxels for decoding object identity using independent localizer data. Thus, the ROIs represent voxels that carry information about stimulus identity, across three different levels of the ventral visual stream. All three levels of the hierarchy were considered interesting, as it was not clear whether expectations may modulate responses in similar or distinct ways across the visual hierarchy.

### fMRI outcome metrics

The empirical and the simulated fMRI data were analyzed using the same analysis pipeline. In total, seven different outcome metrics were assessed. The reasoning for relying on this large number of diverse outcome metrics is based on Alink *et al.* [19], showing that, in the context of stimulus adaptation, neural models can show great flexibility in fitting empirical fMRI results and that only by combining a range of outcome metrics one can successfully distinguish between the best performing models. We modified and extended the set of outcome metrics, resulting in the analyses summarized below. The utilized outcome metrics are based on previous studies investigating the neural mechanism underlying expectation suppression, building on a diverse set of studies supporting sharpening and dampening accounts.

#### Mean amplitude modulation

Mean amplitude modulation probes the univariate differences in response amplitude between expected and unexpected stimuli. Thus, this metric indexes the commonly reported expectation suppression effect [9, 10, 14, 17, 26, 45, 46].

#### Within-class correlation

Within-class correlation assesses the correlation of neural patterns between different presentation instances of the same object stimulus, and potential difference in the size of this correlation between expected and unexpected occurrences of the stimuli. Thus, a large within-class correlation coefficient indicates that the same stimulus, presented on different trials, is represented in a similar fashion.

#### Between-class correlation

Like the within-class correlation, between-class correlation measures the correlation in neural responses. Between-class concerns the correlation in representations between different object stimuli. A low between-class correlation thus indicates that different stimuli elicit dissimilar response patterns. Parameter estimates are z-scored before within-class correlation and between-class correlation calculation. The two correlational metrics (within-class and between-class correlation) are similar to representational analyses used by Blank and Davis [14]. The within-class and between-class correlation metrics were modified compared with their use in Alink *et al.* [19] to account for challenges specific to the study of expectation suppression and the fMRI paradigm used in the empirical data acquisition. Specifically, due to the sluggishness of the BOLD response, the obtained data represented the combined response to leading and trailing images (each stimulus presented for 500 ms without ISI). Given that each expected trailing image was always preceded by the same leading, while unexpected trailing images could be preceded by various leading images, the average correlation would be highly biased (larger) in favor of the expected condition. We accounted for this by calculating the correlation metrics between an expectation free localizer run (i.e. presentation of the trailing images in isolation, without leading image) and the average pattern elicited by the stimuli during the main task split into the two expectation conditions.

#### Classification performance

Classification performance is defined as the difference between between-class and within-class correlations, similar to the classification approach originally outlined in Haxby *et al.* [47]. A higher classification performance thus indicates that object representations are more distinct. As above, data were z-scored. The classification metric is indexing similar information as assessed by Kok *et al.* [9], Yon *et al.* [10] and Han *et al.* [15], albeit using differences in correlation instead of classifiers such as support vector machines.

#### Amplitude modulation by amplitude

Amplitude modulation by amplitude concerns the magnitude of the amplitude modulation (i.e. expectation suppression = response_unexpected_ - response_expected_) as a function of mean voxel amplitude. In other words, it expresses whether the amount of expectation suppression increases (or decreases) as a function of the average responsiveness of a voxel within an ROI. This metric thereby indexes whether expectation suppression scales with general responsiveness. Data were binned into 10 equally sized bins. The responsiveness ranking was established on independent localizer data. This analysis is based on Alink *et al.* [19].

#### Amplitude modulation by selectivity

Amplitude modulation by selectivity, similar to amplitude modulation by amplitude, also expresses the magnitude of expectation suppression, but as a function of voxel selectivity. Selectivity is established based on independent localizer data, by fitting a GLM to each voxel’s response regressed onto the response amplitude ranked images. Thus, a highly selective voxel responds strongly to some images and weakly to others, while a low selectivity voxel responds similarly to all images. Amplitude modulation by selectivity thereby assesses whether the response selectivity of a voxel correlates with the amount of expectation suppression experienced by that voxel. Note: the ROI masks contain the most informative voxels concerning object identity decoding (see *ROI masks*). Thus, all voxels in the ROI are likely to be stimulus driven. The amplitude modulation by selectivity metric is based on Richter *et al.* [17] and Alink *et al.* [19].

#### Image preference

Image preference indexes the amount of expectation suppression within each voxel as a function of image preference. Image preference is established based on localizer data and ranks the response of a voxel to each trailing image by amplitude (i.e. image preference rank). Thereby, it expresses whether the magnitude of expectation suppression differs within a voxel depending on whether the displayed image is a preferred or non-preferred stimulus for this voxel. Image preference is an addition to the set of outcome metrics used by Alink *et al.* [19] and hence worth disseminating in more detail. It contributes additional information over the previous metrics because it indexes expectation suppression as a function of image selectivity (preference) within a voxel, rather than as a function of average selectivity across voxels, like the amplitude modulation by selectivity metric introduced above. Indeed, the slope of image preference can be independent of the amplitude modulation by selectivity slope. Consider for example that suppression would be larger in more selective voxels (i.e. a positive slope of amplitude modulation by selectivity). In this case, it is possible that these highly selective voxels suppress responses for preferred stimuli compared with less preferred stimuli, thus resulting in a positive image preference slope. However, it is also plausible that these selective voxels suppress non-preferred stimuli compared with preferred ones, thereby yielding a negative image preference slope. This outcome metric is based on Richter *et al.* [17] and Kok *et al.* [9].

### Feature space

For each ROI a feature space was defined on a neural response theoretical basis. V1 neurons are thought to be orientation selective [32], and thus V1 feature space was defined by the predominant orientation of the stimulus. LOC has been shown to represent shape complexity [33], which formed the basis of the LOC feature space. TOFC represents complex visual features, which appear related along semantic categories [34], thereby suggesting a feature space representing semantic similarity. Thus, for each ROI, a 1D feature space was constructed, along which each object stimulus could be expressed as a point in feature space. Finally, each feature space was validated using RSA, performed on independent localizer fMRI data. For a feature space to be considered usable, it should account for a statistically significant amount of neural response variance in its designated ROI (see *fMRI data analysis for feature space validation*).

#### V1 feature space

For primary visual cortex, feature space was defined by the predominant orientation of each object stimulus. To this end Gabor filters of different frequencies (from $4/\sqrt{2}$ to the hypotenuse of the length of the input image; [48]) and orientations (in steps of 20 degrees) were constructed, and the Gabor energy for each orientation extracted. Energy was averaged over the different spatial frequencies and the orientation with maximal energy was used. This orientation thus represents the maximal orientation energy present in the object stimulus and thereby determined the position of a stimulus in V1 feature space. Figure S5 shows the arrangement of the object stimuli in orientation feature space. Given that orientation is circular, this feature space was modeled as circular (i.e. feature space ranged from 0 to π). The resolution of feature space was set to 180 * π.

#### LOC feature space

For LOC, feature space was defined by shape complexity. Following Vernon *et al.* [33], we calculated several metrics describing the complexity of each object’s shape. These metrics included (i) the number of concavities, as well as (ii) the area of these concavities, (iii) the area and (iv) perimeter of the smallest convex hull encompassing the object and (v) the area of the smallest circle enclosing the object, as well as (vi) the ratio of the silhouette of the object and the smallest circle. In brief, the number and area of the concavities describe shape complexity because the number of concavities is directly related to the number of protrusions, which in turn is considered a metric for complexity (i.e. more complex shapes have more protrusions). Similarly, the area and perimeter of the smallest convex hull, encompassing the object, will be larger for complex objects with more protrusions. The area and ratio of the smallest circle encompassing the object relative to the silhouette indexes how compact an object is, with less compact (complex) objects yielding larger values. Because we aimed to describe feature space along one dimension for each ROI, the shape complexity metrics were subjected to a principle component analysis. Finally, the first principle component was extracted and used as the primary shape complexity descriptor. The arrangement of the stimuli in shape complexity feature space is depicted in Fig. S6. As the shape complexity metric is arbitrary in its range relative to LOC responses (only relative positions and distances are interpretable), the same feature space resolution was used as for V1. However, LOC feature space was not circular, as the shape complexity dimension arranges objects from the most to the least complex (i.e. non-circular).

#### TOFC feature space

For TOFC, feature space was defined by semantic similarity. The central idea is that TOFC responses are related to complex visual feature, which are correlated along semantic categories [34]. We used a multiple-arrangement task [49], in which a separate sample of participants (*n* = 32; 16 females; age 26.9 ± 8.5 years) arranged object stimuli in an arena by their semantic similarity. Participants were instructed to arrange each object display by the similarity of the objects. It was emphasized that the arrangement should be made along semantic/categorical similarities, and not by low level features. Distances between objects thus represent the semantic dissimilarity between each object pair. Sixteen participants arranged the object arrays that the fMRI participants in Richter *et al.* [17] were exposed to, while the other 16 participants arranged the objects shown to participants in Richter *et al.* [26]. Each arrangement trial was the object matrix shown to one participant in the fMRI studies, thus constituting the context in which participants were exposed to the object stimuli, as semantic similarities are sensitive to context (e.g. cats and dogs are more similar to one another in a set of images also containing inanimate objects but more dissimilar in a set comprised only of mammals). In addition, randomly sampled arrangements, as well as one arrangement of all object images in the database for each study, were appended until 75 minutes of experiment time passed. Finally, the individual RDMs were collapsed by averaging, thus resulting in one RDM for the stimulus set of each fMRI experiment, which describe the human-rated, semantic dissimilarity (distance) of each object to each other object. Finally, multidimensional-scaling was used to extract a single dimension to describe the position of each object along the semantic similarity feature space. Figure S7 depicts the object stimuli arranged along the subjective similarity feature space. The precise values of this TOFC feature space are arbitrary, and only relative positions are meaningful. As with LOC, the feature space was non-circular.

#### Feature space comparison

In a validation of the feature spaces, we assessed the similarity of the stimulus arrangements in the three feature spaces. We compared the RDMs of the three feature spaces using RSA. Average correlations between the model RDMs of the three feature spaces were calculated as Spearman’s Rho and bootstrapped 95% confidence intervals were obtained by resampling each RDM 10 000 times. *P* values, comparing the obtained correlation coefficients, were calculated using a Mantel test.

#### fMRI data analysis for feature space validation

Each participant’s fMRI object localizer run was analyzed in an event-related approach using FSL FEAT, modeling each object stimulus as a regressor of interest. Twenty-four motion regressors (FSL’s standard + extended set of motion parameters), as well as other regressors of no interest (instruction screen) were added to the model. The contrasts of interest were the parameter estimate maps representing the responses to each object image compared with baseline (no visual stimulation). These contrast parameter estimates were extracted for each ROI and used to validate the three above described feature spaces.

To validate the feature space for each ROI, we performed RSA. In brief, for each participant of the MRI experiments, the positions in feature space of the objects shown to that participants were determined using the above outlined feature space models. From these positions in feature space, a RDM was calculated describing the dissimilarity between each object pair in the relevant feature space, i.e. for V1 distance in predominant orientation, for LOC distance in shape complexity and for TOFC distance in semantic category. Next, the neural RDM was constructed by extracting the parameter estimates from the localizer run for each ROI separately (also see *ROI masks*). The parameter estimates were z-scored and pairwise correlations between object representations were calculated. Thus, this RDM (1 – correlation) indexed the neural representational dissimilarity during the localizer run. Finally, we correlated the neural and feature space RDMs for each participant and ROI using Spearman’s rank correlation. We calculated partial correlation coefficients, including the other two feature space RDMs as control variables, to assess the unique contribution of each feature space. This correlation coefficient describes the correlation between the feature space RDM and the neural RDM for each participant and ROI. After Fisher z-transforming the obtained correlation coefficients, data were combined across participants by subjecting the obtained correlation coefficients to a two-sided, one-sample t-test, comparing the correlation coefficients against zero (i.e. no correlation). A significant, positive correlation would thus indicate that the constructed feature space does account for variance in the neural responses, which was considered a requirement for each feature space in its designated ROI.

### Simulation

The following section first describes the simulation of neural responses and their modulation by expectations. Next, we outline the sampling of neurons to voxels, as well as the estimation and addition of noise to the voxel responses. Finally, requirements imposed on simulated neural responses are described, as well as the analysis of model fits.

#### Neural responses

**Figure 8:**
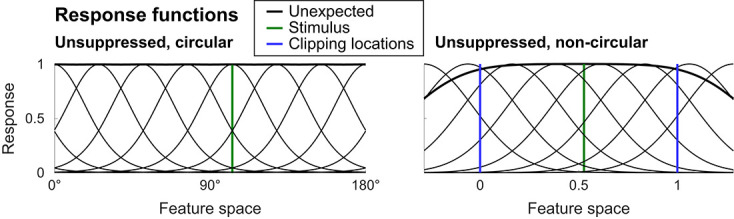
Unsuppressed neural response functions. Depicted is an example set of unmodulated neural response functions (i.e. response to an unexpected stimulus). The left panel shows response functions covering a circular feature space (V1), while the right panel shows a non-circular feature space (LOC or TOFC). The thin lines are individual response functions with different means along the feature space. Summed responses (normalized to one) are shown as the thick line. The vertical lines denote an example stimulus and the clipping boundaries of the non-circular feature space—i.e. the region of feature space in which all stimuli are placed. The boundary is determined for each non-circular feature space by the minimal response criterion of 95% (see *Neural responses*).

We modeled neural responses using neural response functions in ROI specific feature spaces. In the simplest case, a neural response was described as a Gaussian distribution with a mean in feature space and a standard deviation (σ). The full feature space was covered with eight neural response functions, each with a different mean, following the implementation of Alink *et al.* [19]. The maximal response of each neural population was normalized to one. Given that orientation feature space is circular, the circular normal (von Mieses) distribution was used for modeling V1 responses. Figure 8 (left panel) shows an example for V1. As the veridical standard deviation (σ) is unknown, σ was a free parameter in the model.

**Figure 9:**
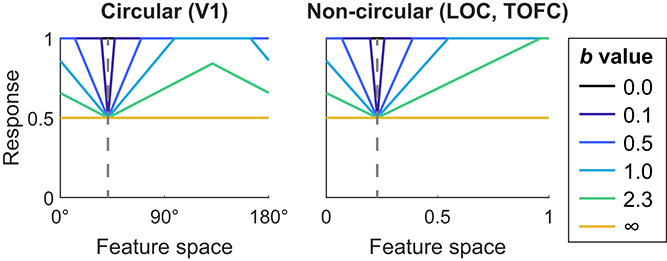
Influence of the b parameter on suppression magnitude. Illustration of response suppression as a function of distance from an expected stimulus in feature space for different values of b. The dashed vertical lines indicate the position of an example stimulus in feature space. The example model is local gain modulation with a fixed a parameter value of a = 0.5 for illustration purposes. As the value for b increases (brighter colors), the broader the suppression profile becomes. For b = ∞ local/remote gain modulations are equal to global gain modulation. Grid search for b was limited to 0.1 > = b < = 2.3. Note the extreme locality of suppression for small b values. For example, for b = 0.1 neuronal populations tuned approximately 5 degrees away from the expected stimulus orientation would not experience any suppression. On the other extreme, for large b values, such as b = 2.3, local gain modulations would still substantially (suppression below 80% of responsiveness) affect neural populations tuned to exactly the opposite orientation of the expected stimulus (i.e. 90 degrees away in V1 feature space).

Because non-circular feature spaces yield significantly lower summed responses at their extremes, due to a lack of overlap of response functions beyond the boundary of the feature space (see Fig. 8, right panel), each non-circular feature space was clipped to the central region of feature space in which the summed response exceeded at least 95% of the maximal response. In other words, this criterion avoids that some stimuli elicit a significantly reduced response simply due to its position at the extremes of feature space, as there is no theoretical or bilogical reason why a stimulus at the end of, for instance the semantic category feature space, should elicit lower responses in TOFC than a stimulus in the center of that feature space.

#### Modulation by expectations

Next, neural responses were modulated by expectations, if a trial contained an expected trailing image, thus implementing a modulation of the neural response following a top-down modulation. This modulation can for instance result after recurrent message passing, in line with predictive coding accounts, representing a more extensive resolution of prediction errors for expected input. We did consider, and ruled out, alternative model implementation in which expectations are instantiated irrespective of an expected stimulus being shown. However, only local (dampening) models could result in expectation suppression, the core phenomenon of interest, in this alternative implementation. Thus, these alternative implementations rule out remote (sharpening) and global models by design. For a more detailed discussion and illustration, see Text S2 and Fig. S2. In the present stimulation, the suppression of neural responses by expectations was modeled using two primary classes of modulations. Gain modulation models reduced the response of a neuron in a linear fashion—in Alink *et al.*, referred to as ‘scaling’ models [19]. The magnitude of suppression was determined by a free parameter (*a*). In the simplest case, *a* was an unspecific multiplicative reduction, i.e. if *a* = 0.7, the maximal response was reduced to 70% and all other responses were scaled proportionally in the global gain modulation model. On the other hand, tuning models modulated responses by reducing the width of the response functions—in Alink *et al.*, referred to as ‘sharpening’ models [19]. Thus, this modulation did not affect the maximal response of a neuron but sharpened its response by making the responses more selective, with the *a* parameter determining the extend of this tuning modulation.

Besides the two main model types (gain modulation and tuning), we also modeled three different distance functions. Distance functions determined where the effect of the response modulation occurred, relative to the expected stimulus in feature space. Global models affected the response functions equally across parameter space and do not have the parameter influencing the distance function. Local models exerted the modulation for neural populations close to the expected stimulus in feature space and reduced in their modulation strength the further a neural population was tuned away from the expected stimulus in feature space. Remote models were opposite to local models, as they exerted the influence at the opposite side of feature space from the expected stimulus. A free parameter (*b*) influenced the distance over which the response modulation changed, i.e. large *b* parameter resulted in a broad influence across feature space. In fact, remote and local models are equivalent to global models if *b* = ∞. Figure 9 illustrates the effect different *b* parameter values have on suppression magnitudes across feature space.

The combination of two model types and three distance functions resulted in six models, which were used to describe response modulations by expectations. Thus, the response to an expected stimulus was the sum of the modulated response functions at the point in feature space of that expected stimulus. Figure 3B shows the six models in response to an example stimulus.

#### Model formulation

Model types:

Gain modulation: ${f}_i(j)=c(i,j)\times g({x}_j;{\mu}_i,\sigma )$

Tuning:

\begin{equation*} {f}_i(j)=g\left({x}_j;{\mu}_i,c\left(i,j\right)\times \sigma \right) \end{equation*}

**Distance functions:**

Local:

\begin{equation*} c\left(i,j\right)=\min \left(1,a+\left|\frac{d\left(i,j\right)}{b}\right|\ \left(1-a\right)\right) \end{equation*}

Remote:

\begin{equation*} c\left(i,j\right)=\mathit{\max}\left(a,1-\left|\frac{d\left(i,j\right)}{b}\right|\ \left(1-a\right)\right) \end{equation*}

Global:

\begin{equation*} c\left(i,j\right)=a \end{equation*}

where

${f}_i(j)$ = activity of neural population *i* given stimulus *j*;

$c(i,j)$ = suppression given stimulus *j*, neural population *i* and applicable distance function;

${x}_j$ = position of stimulus *j* in feature space;

${\mu}_i$ = mean of neural population *i* in feature space;

$\sigma$ = width of response function (free parameter);

$a$ = suppression magnitude parameter (free parameter);

$b$ = distance parameter (free parameter);

$d(i,j)$ = distance of stimulus *j* to the mean of the neural population *i* in feature space.

#### Parameter grid

Given the above formulation, the present simulation has one free parameter determining the unmodulated response: the width of the neural response functions (σ). Furthermore, the modulated response functions contain two additional free parameters: the amount of suppression (*a*) and the effect of distance in feature space (*b*). A wide grid search was utilized to cover plausible parameter combinations (also see *Response requirements*). The σ parameter value ranged from 0.1 to 6. Given the feature space width of π, these values represent a large range of σ values. Step size for σ was 0.1 from 0.1 to 1, 0.5 from 1 to 4 and 1 from 4 to 6. The *a* parameter was explored from *a* = 0.05 to 1, in steps of 0.05. An *a* parameter value of 0.05 corresponds to maximal suppression (e.g. for global gain modulation models, this would result in a reduction of neural responses by 95% due to expectations), while *a* = 1 corresponds to no modulation by expectations. The final parameter, *b*, spanned values from *b* = 0.1 to 2.3 (i.e. approximately ¾ π), in steps of 0.1. Small *b* values represent maximal locality of modulations, while for *b* = ∞ local/remote would be identical to global models. We limited *b* to 2.3 in order to properly distinguish global from non-global models. However, it should also be noted that *b* = 2.3 already represents a fairly global modulation, given that feature space ranges from 0 to π (see Fig. 9). Combined, the three parameters resulted in a total grid size of 7820 parameter combinations, which were explored for each of the six model types. As a grid search was utilized, two concerns need to be addressed. One, the parameter grid must cover the whole parameter space of interest, i.e. the explored grid needs to be broad enough and/or bounded by theoretical or mathematical reasons. Two, the step size of explored values must be sufficiently small to accurately sample the error landscape. We addressed both concerns by showing that the error landscape was smooth and contained the minima well within the explored bounds, which additionally were limited by theoretical considerations. Results and an additional discussion of the parameter ranges are presented in Supplemental Figs S8–S10.

We chose not to include the number of tuning functions as a free parameter in favor of expanding the size of the explored parameter grid to the above noted 7820 parameter combinations, compared with 648 in Alink *et al.* [19]. This expansion of the explored parameter values was necessary as our stimuli occupied the whole feature space instead of only two extremes. Crucially, we do not rely on adding a fixed amount of noise to each model iteration, but rather estimate appropriate noise levels for each unmodulated response model (for details, see *Noise estimation* below), thereby addressing the concern that tuning function granularity correlates with signal strength [24].

### Simulating voxels

Biased sampling [35] is arguably a leading account of how stimulus selectivity arises in fMRI voxel data. Simplified, the idea is that voxels pool over millions of neurons in a biased fashion, with different neural tunings being overrepresented in different voxels. Alternatively, global biases (maps) have been suggested to underlie the large scale response preferences evident in voxel-level data [36]. As in Alink *et al.* [19], we used a simple implementation in line with both macroscale maps and biased sampling, by random sampling a limited number of neurons with different feature tunings to form simulated voxels (eight per voxel). Spatial information was not modeled in the present simulation, as none of the ROI based analyses methods utilize spatial information beyond classical multivariate pattern analysis. The consequence of this limited random sampling procedure was simulated voxel-level data, which showed response preference for different stimuli. While this approach certainly constitutes a crude method for sampling neurons to form voxels, bypassing the complexities involved in the mapping of neural responses to BOLD signals (hemodynamics, etc.), it does succeed in creating voxels, which mirror the response profile seen in empirical fMRI data. In fact, the central assumptions of this approach are only that voxel-level selectivity reflects in an indirect manner neural tuning and that there is some monotonic relationship between neural activity and voxel responses. To further improve the similarity of the simulated and empirical data, we created the same number of voxels as were analyzed in the empirical fMRI data and added a customized amount of noise to the simulated voxels.

#### Noise estimation

For each ROI and σ parameter combination, we added a custom amount of Gaussian noise to the voxel responses. The appropriate noise magnitude was determined by performing a separate SVM-based decoding analysis on the empirical localizer data and simulated localizer data. In brief, unmodulated neural responses to example stimuli were simulated and decoding was performed using linear SVMs. Iteratively more noise (noise parameter, the SD of the Gaussian distribution increased in steps of 1) was added to the simulation until the decoding performance of the simulated data was less than the decoding performance of the empirical fMRI data. Subsequently, the noise value yielding results closest to the empirical results was chosen as the noise level for the ROI and σ combination. This approach differs from Alink *et al.* [19] and addresses one of the central issues outlined by Ramírez and Merriam concerning the influence of (implausibly low) noise levels [24]. Estimating custom noise levels, we aimed to ensure that no σ value is biased due to significant SNR advantage. Moreover, estimating noise levels further increases the comparability of the simulated and empirical data in terms of SNR and accounts for potential effects of ROI specific SNR levels on the observed results.

#### Response requirements

Given that the precise parameter values resulting in the most biologically plausible responses are unknown, we performed a broad grid search across parameter space. However, a broad grid search will inevitably result in some parameter combinations yielding implausible neural responses. The aim of the current study is to elucidate what type of neural modulation may underlie expectation suppression and not to show the theoretical flexibility of unconstraint computational models. Thus, we enforced three biological plausibility criteria to the constructed neural responses spaces (NRSs) that any parameter combination had to fulfill to be considered for the main simulation.

**Figure 10:**
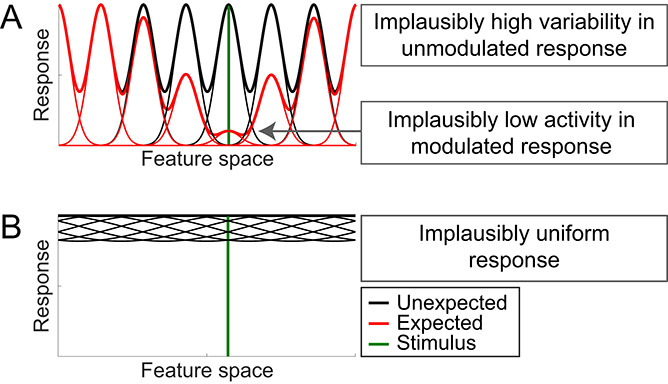
Implausible responses. Shown are neural responses, which were considered biologically implausible and thus rejected from the simulation. (A) shows a neural response space for which the parameterization resulted in an implausibly high variability in the unmodulated neural response (thick dark line). On this parameterization, the maximal unmodulated response to feature values would vary with more than 50%, e.g. the maximum possible responses of any neuron to a stimulus of 100 degrees orientation would be more than 50% larger than the response to a stimulus of 120 degrees. Any difference exceeding 75% in the unmodulated responses was deemed implausible. Note: This does not affect population differences due to an overrepresentation of neurons preferentially responding to specific features but concerns the maximal possible response of any neuron type. The thick bright line shows an implausibly low modulated response, i.e. the maximal response to this expected stimulus would be ~5% of the unmodulated response, which was considered biologically implausible, given that the approximate difference between maximal responsiveness and baseline (no visual stimulation) for expectation-sensitive, visually driven IT neurons is ~24% (see [18]). (B) depicts an example of an implausibly uniform neural response space. Thin lines indicate individual neural response functions, which are implausibly broadly tuned, i.e. the neurons are hardly selective for any feature, as their response exceeds 75% of their maximal response irrespective of the feature, which was deemed implausible.

First, any unmodulated NRS had to cover the feature space reasonably well. This criterion rejects NRSs with too much variability or even ‘holes’ in its responses. An example of an excessively variable NRS is depicted in Fig. 10A. Note that the unmodulated summed neural response (thick black line in Fig. 10A) to some stimuli in feature space is drastically reduced compared with the response to other stimuli. In the case of V1, this would mean that neural responses to an oriented bar of, e.g. 20 degrees would be more than twice as large as the maximum possible response to an oriented bar of approximately 30 degrees. Indeed, more extreme cases would even result in a *de-facto* blindness to certain feature values. While response biases on the population level certainly exist (e.g. in V1 to cardinal orientations; [50, 51]), the variable response criterion enforced here does not concern the population (voxel) response, but the maximal response of any possible neuron to a particular feature value. Given that neural responses, at least to the unmodulated stimuli, should be fairly uniform (i.e. there are at least some neurons that respond to a given stimulus in the relevant feature space), we rejected any unmodulated NRS that had a point in feature space to which the unmodulated summed response was less than 75% of the maximal response. This ensured a reasonably uniform responsiveness to unmodulated stimuli.

Second, a similar minimum response criterion was also enforced for modulated NRSs. However, this criterion was set to a more liberal threshold to allow for a larger modulation range. In particular, the threshold was set such that the modulated summed response to any point in feature space was at least 10% of the maximal summed response. This criterion avoids outright blindness (i.e. zero response) to expected features. To put the threshold of 10% in perspective, one can consider that the ratio of the maximal spiking rates of expectation-sensitive IT neurons (population average) compared with baseline (no visual stimulation) is ~24%, as reported by Meyer and Olson [18]. In other words, we reject NRSs for which the expectation modulation was more than twice as strong as the response to visual stimulation compared with baseline in visually driven IT neurons.

Third, neurons of any unmodulated NRS had to be sufficiently selective. This criterion rejects NRSs in which neural tuning is implausibly uniform, as depicted in Fig. 10B. The rationale for this criterion is based on the notion that feature selective neurons were simulated and that feature selective neurons ought not to respond (almost) equally to all features. In particular, we rejected any unmodulated NRS in which the response of a neuron is >75% of its maximal response ½ π away from its mean. In other words, this criterion precluded neurons that would respond maximally to an orientation of 0 degrees and still respond with >75% of its maximum to orientations of 90 degrees.

In total, these response requirement criteria resulted in the rejection of 3.2% of NRS in V1 and 42.2% in LOC and TOFC. Rejection numbers were noticeably larger in LOC and TOFC because V1 feature space was circular, utilizing von Mieses distributions instead of Gaussian distributions in LOC and TOFC. Thereby, the number of NRS with too low responses for any feature space position for very small σ values, or too unspecific responses for large σ values in the unmodulated NRS, was significantly lower in V1 than LOC and TOFC. However, more important than differences in rejection percentages between ROIs were possible differences between model types within the same ROI. Differences between model types were relevant to consider, as radically different rejection percentages would result in some model types having more valid parameterization, and thereby a higher chance of fitting the empirical results simply due to a larger number of valid simulations. Reassuringly, differences in rejection percentages between different model types were less than 10% between almost all model types in all three ROIs. Details are summarized in Table 1. Thus, each model type was sampled approximately equally often, with minor differences due to poor feature space coverage being slightly more prevalent in some model types (see rejection criteria above). In sum, the similar rejection rates imply that any potential differences in the performance of different model types are unlikely to be explained by differences in the number of sampled parameter combinations. Moreover, NRS rejections ensured that the obtained results are more likely to be meaningful, as the implemented rejection criteria support biological plausibility of the considered models.

**Table 1:** Percentage of neural response space rejections

	ROI	
Model types	V1	LOC and TOFC
Global gain modulation	10.0%	41.8%
Local gain modulation	0.2%	35.5%
Remote gain modulation	2.0%	39.0%
Global tuning	4.1%	46.8%
Local tuning	0.7%	38.5%
Remote tuning	2.4%	45.9%

Shown are the percentages of neural response space rejections for each model type and ROI. While there are large differences in rejection rates between circular (V1) and non-circular (LOC and TOFC) feature space ROIs, importantly within each ROI rejection rates between models are similar (differences of usually <10%). This suggests that differences in the number of sampled parameter combinations are unlikely to account for differences in the observed results, as all model types are sampled approximately equally often.

#### Simulation procedure

After establishing the model formulation and creating voxels per simulated participant, we proceeded to present object stimuli to the simulated voxels. In fact, we simulated the presentation of the same stimuli, on the same number of trials as was used in the empirical data collection. In other words, each participant of the empirical fMRI data was simulated with the original trial matrix. We simulated each participant ten times to ensure that results were not driven by noise, resulting in a total of *n* = 560 simulated participants. Data for each participant was simulated for each of the three ROIs, using each of the six model types across the 7820 unique parameter combinations, thus resulting in a total of 78.8 million (560 × 3 × 6 × 7820) simulated subject data points. Additionally, we also simulated localizer data by adding ½ of the estimated noise level, thereby mimicking the higher SNR afforded by the localizer run’s design, again using the same number of simulated localizer trials as in the empirical localizer data. Finally, the simulated fMRI responses were analyzed using the same analyses as for the empirical fMRI data (see *fMRI outcome metrics*).

#### Model comparison

The central question in the present study concerns which type of neural modulation best explains the empirical results. Frequently, in fMRI studies, statistically significant outcome metrics are interpreted in a binary fashion, i.e. either the effect reduces or increases the response. Mimicking this binary approach, we first analyzed our data by comparing the sign of statistically significant simulated outcome metrics with the empirical results. Thus, in this analysis a model either can or cannot account for a particular outcome metric. By summing the number of matching slopes, we can determine how many outcome metrics each model can account for. Additionally, we assessed how robust the results are to changes in the precise parameterization, i.e. how many different parameterizations of each model could explain a large number of outcome metrics. To this end, we calculated for each model type the percentage of valid model parameterization (i.e. parameter combinations that fulfilled the criteria listed in *Response requirements*) that resulted in the maximal number of matching signs of slopes. This metric thus gives an indication not only how well each model type can account for the sign of the slopes under one ideal parameterization, but how robust the result is to changes in the parameter values, with more robust models being preferable.

Besides this binary approach, we also performed a more fine-grained quantitative analysis of the model fit. In particular, we calculated a weighted MSE for each model type and parameter combination. We first calculated the normalized slope for each outcome metric and model parameterization. A normalization step was necessary because the empirical and simulated data, depending on the outcome metric, encompass different numerical ranges. Normalization was done by dividing the modulated response by the average unmodulated response per outcome metric (for mean amplitude modulation, within-class and between-class correlation and classification performance). Thus, for instance normalized mean amplitude modulation expresses how much the average response to an expected input is suppressed relative to the unmodulated response. For all outcome metrics indexing ranked expectation suppression (amplitude modulation by amplitude and selectivity, as well as image preference), the suppression score was divided by the unmodulated average BOLD response. The normalized image preference metric for instance, therefore, indicates how strong expectation suppression is per image relative to the unmodulated response. Next, the slope for each outcome metric was calculated on these normalized values. We then compared these slopes for each simulated result (i.e. model type and grid point combination) to the empirical fMRI results by calculating the squared error. The squared errors per outcome metric were scaled by dividing the errors by the grand median per outcome metric (i.e. the median per outcome metric across model types and parameterization). This scaling step ensured that each outcome metric on average contributes roughly equally to the assessment of the overall model fit, following the rationale of the approach by Alink *et al.* [19] to analyze a combination of several fMRI outcome metrics. The scaled squared errors were then averaged across outcome metrics; hence, each model and parameter combination resulted in one MSE value, with the smallest MSE constituting the model that most closely fit the empirical fMRI results. In this final averaging step, we used the inverse of the normalized SD (SD/mean) across participants to weight the relative contribution per outcome metric to the MSE. The rationale of using a weighted averaged is to reflect the reliability across participants of the outcome metrics in the MSE, thereby discounting metrics with high interindividual variability (akin to calculating a t-score). MSEs were calculated for each ROI separately and averaged across participants.

We investigated the resulting MSEs by displaying the lowest MSE per model type as well as the 2% (*n* = 156) best fitting parameter combinations for each model type. Both assessments of the results were considered important, since the lowest MSE model best describes the empirical data. However, also the robustness to changes in the exact parameterization was deemed crucial, as a reliable fit across multiple parameterizations indicates that a model type can fit the empirical data well without requiring exact parameter tunings and thereby increasing the likelihood that the modulation underlies the neural responses in the empirical data.

Finally, we inspected the parameter values under which the models performed well. For this purpose, we were interested in the *a* parameter, as the best parameter value may provide additional information in characterizing the neural mechanism underlying perceptual expectations. In particular, the *a* parameter value gives an indication of the suppression magnitude.

## Supplementary Material

Supplementary material is available at *Oxford Open Neuroscience* online.

## Study Funding

This work was supported by The Netherlands Organization for Scientific Research, Vidi Grant 452-13-016 awarded to F.P.d.L. and the EC Horizon 2020 Program ERC Starting Grant 678 286 ‘Contextvision’ awarded to F.P.d.L.

## Conflict of interest

None declared.

## Authors’ contributions

Conceptualization: D.R., M.H., F.Pd.L.; data curation: D.R.; formal analysis: D.R.; funding acquisition: F.Pd.L.; investigation: D.R.; methodology: D.R., M.H.; project administration: D.R.; resources: D.R.; software: D.R., M.H.; supervision: F.Pd.L.; validation: D.R.; visualization: D.R.; writing (original draft): D.R.; writing (review and editing): D.R., M.H., F.Pd.L.

## Ethics and consent

The conducted experiments followed institutional guidelines of the local ethics committee (CMO region Arnhem-Nijmegen, The Netherlands; METC no. 2014-288) and were in accordance with the Declaration of Helsinki.

## Data availability

MRI data were preprocessed and analyzed using FSL 6.0 (FMRIB Software Library; Oxford, UK; www.fmrib.ox.ac.uk/fsl; [42]; RRID:SCR_002823). Additional fMRI data analysis was performed using custom Python (Python Software Foundation, RRID:SCR_008394) and Matlab 2018b (The MathWorks, Inc., Natick, Massachusetts, United States, RRID:SCR_001622) scripts. The simulations were performed using Matlab 2018b. Data and code are available in https://doi.org/10.34973/2jxe-d940.

## Supplementary Material

suppl_material_kvac013
