# Supplementary material for: Dampened sensory representations for expected input across the ventral visual stream
Source: Oxf Open Neurosci. 2022 Aug 15;1:kvac013. doi: 10.1093/oons/kvac013 (PMC10939312; doi:10.1093/oons/kvac013)
Supplement: suppl_material_kvac013 [file suppl_material_kvac013.zip › RHDL_2022_supplementary_material.pdf]

Supplementary Material

S1 Text. Model recovery

In a control analysis we sought to establish that our forward modelling approach can recover known model types used to generate simulated fMRI data. If successful, this analysis supports that the modelling approach reliably differentiates between the six model types, thereby increasing confidence that inferences about neural modulation underlying the empirical fMRI data are valid.

To this end we first generated several fMRI datasets using the same modelling procedure as used during the main analysis (see Materials and Methods, Simulation). The only change was that we used the forward models to generate fMRI datasets, with added Gaussian noise (SD = 1). For fMRI data generation we subsampled the parameter grid (see Materials and Methods, Simulation, Parameter grid) in equal steps to cover a broad range of datasets. The exact parameter values used to create the fMRI datasets were a = [0.3, 0.6, 0.9], b = [0.7, 1.5], σ= [0.3, 2.0], representing a selection of values throughout the whole parameter space. Additionally, we varied the number of tuning functions covering the feature space; n tuning functions = [8, 16, 32]. This additional manipulation allowed for more variation in the generated datasets, including parameter combinations not assessed in the recovery grid, increasing the difficulty of success model recovery. In total this resulted in a parameter grid of 36 combinations. Moreover, we generated data for each of the three ROIs of interest (V1, LOC and TOFC) in the main analysis, because each ROI is modelled by a different feature/stimulus space. The six model types (local gain modulation, local tuning, remote gain modulation, etc.) were used to generate one dataset for each ROI and parameter combination, thus resulting in a total of 3 (ROIs) x 36 (parameter combinations) x 6 (model types) = 648 generated fMRI datasets. Each generated dataset encompassed the same number of simulated participants (n = 34) and trials per participant (n = 240) as in the empirical fMRI dataset from Richter and de Lange [26].

Next, we applied the same forward modelling pipeline for model recovery, as used for the analysis of the empirical fMRI data. However, we limited the searched parameter grid to a subsample of the full parameter grid used for the empirical data (see Materials and Methods, Simulation, Parameter grid)– this reduction was necessary due to the computational cost of running the forward modelling pipeline on 648 generated datasets, instead of only two combined empirical fMRI datasets. Specifically, we reduced the parameter grid to values of a = 0.15 to 0.9 in steps of 0.15, b = 0.3 to 1.9 in steps of 0.4, and σ = [0.3, 0.6, 0.9, 2, 4]. Except for the reduced parameter grid size, the forward modelling procedure was identical to the main analysis.

Finally, we analyzed the percentage of generated datasets for which the correct model type was recovered by our forward modelling pipeline. For this we used several outcome metrics per model type, with the results averaged across the three ROIs. First, we assessed the average MSE per model type, split into datasets where the model was used to generate the underlying data and where another model type was used to generate the fMRI data. Results, depicted in Figure S1A, showed that MSEs were consistently lower for each model type when that model was used to generate the fMRI data. These results confirmed that our forward models fit the fMRI data better when the associated model generated the underlying data compared to datasets where another model type generated the fMRI data. Additionally, dampening models (red) and sharpening models (blue) did not show any systematic differences in their average MSE, suggesting that our modelling pipeline was not biased towards lower MSEs for dampening models.

Figure S1B shows additional model recovery metrics further supporting the reliability of the analysis pipeline. In particular, the left panel in Figure S1B shows how often the correct (that is generating) model type was recovered as the model with the lowest MSE (akin to main result Figure 5A) across all generated datasets, split into each model type. Thus, on average all models could be reliably recovered by finding the model with the lowest MSE, except for the global tuning model. Additionally, the middle panel of Figure S1B shows the percentage of model types among the top 2% of lowest MSE models, similar to assessing the model fit across multiple parameterizations as in main result Figure 5B. These results showed that on average a larger proportion of models with low MSEs belonged to the generating model type than expected by chance (dashed line). Finally, the right panel in Figure S1B depicts the total frequency that each model type was found to have the lowest MSE irrespective of the generating model type. Crucially, the latter result indicated that there was no bias in favor of recovering the dampening (local gain modulation) models over the sharpening (remote gain modulation) model irrespective of the generated fMRI data. In fact, the sharpening model was more frequently recovered in total.

In sum, none of the model recovery metrics yielded evidence of a bias favoring dampening models. That is, the recovered average MSEs (Figure S1A) appeared comparable between dampening and sharpening models, as did the percentage of successful model recoveries (Figure S1B, left and middle panels) and the frequency of model types yielding the lowest MSE (Figure S1B, right panel). Thereby, this control analysis suggested that the better fit of dampening models in the analysis of the empirical fMRI data did reflect a true superior fit of the dampening models to the empirical data instead of a bias in the modelling approach. Moreover, the successful recovery of generating model types did support the validity and reliability of the forward models and the utilized outcome metrics.

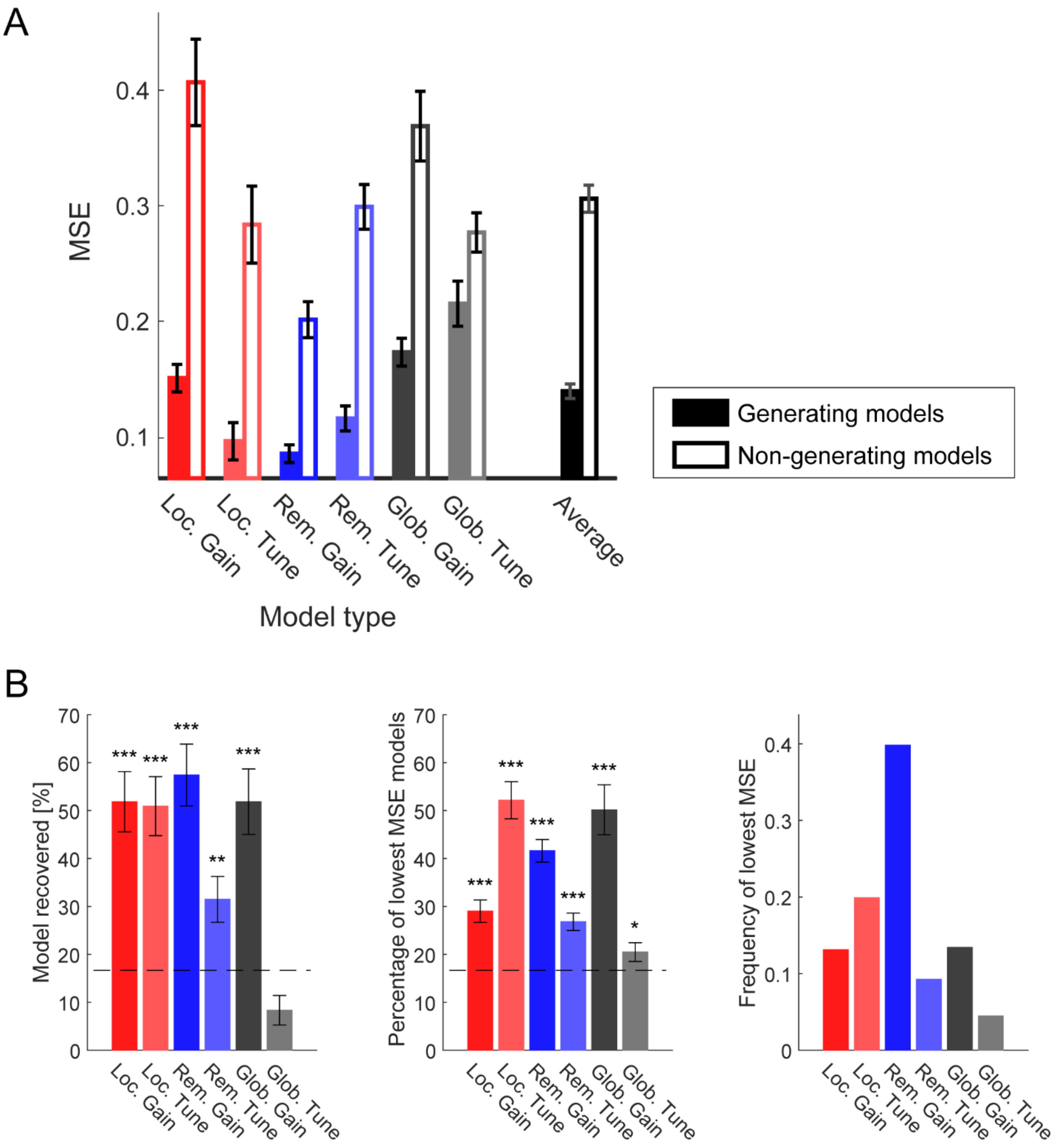

S1 Fig. Model recovery from generated fMRI data

Shown are the results of a model recovery control analysis, demonstrating that models used to generate simulated fMRI data can be recovered using the forward modelling pipeline. **A**, Shows the average MSE (lower is better) per model type split into generating and non-generating model types. Confirming the reliability of the analysis pipeline, all MSEs were smaller for each model type when that model was used to generate the analyzed data compared to the average MSE when that model type was not used the generating model. **B**, Shows model recovery statistics indicating successful model recovery (higher is better). The percentage of correct generating model recovered by

the forward modelling pipeline are depicted per model type in the left panel. All except one model type could be reliably recovered from the generated fMRI data. Horizontal dashed lines indicate chance level. The middle panel depicts the percentage of correct (generating) model types among the best 2% models with the lowest MSE (akin to Figure 5). Error bars indicate the SEM calculated over generated datasets with different parameter values for each model type (n=36). One-sample t-test results assessing recovery above chance level: * $p < 0.05$. ** $p < 0.01$. *** $p < 0.001$. The panel on the right shows the frequency that each model type was found to show the lowest MSEs across all generated fMRI datasets irrespective of the generating model type, indicating that there was no bias towards recovering the dampening model more frequently than other model types.

S2 Text. Alternative implementations of expectation suppression

In our simulations the neural response was only computed using a modulated response function when the expected stimulus was presented (red curve in Figure 3B). By contrast, when a different (unexpected) stimulus was presented, the unmodulated response function was used to compute the response (black curve in Figure 3B). As such, the response function was conditional on the identity of the stimulus. While such stimulus-conditional response modulations can be conceptualized in biological terms as reflecting a top-down modulatory effect (i.e., a modulation happening after the initial feedforward sweep), it is not the only logically possible way to formalize a modulation. An alternative way would be to formalize response modulations conditional on the expectation. This way, expectations would affect responses not only to expected but also unexpected stimuli. In this case, different stimuli would be affected differently by virtue of their location along feature space, rather than because of the use of a different response function for expected compared to unexpected stimuli. While this alternative formulation aligns with how response modulations were conceptualized for other modulatory effects, such as attention [29], it cannot provide a coherent mechanism that implements both of the main theoretical accounts of expectation suppression; sharpening and dampening. Here, we demonstrate this using a toy simulation.

Figure S2A, illustrates the alternative model definitions in which the responses to both, expected and unexpected stimuli are modulated by expectations. Thus, in these models the modulation by expectations is not conditional on the stimulus being expected, thereby representing not a consequence of recurrent message passing following stimulus presentation (as implemented in the main simulation), but for instance a prestimulus expectation and subsequent suppression of the responses. Crucially, as can be seen in Figure S2A and Figure S2B, only local models can reliably result in expectation suppression in this implementation. In other words, the response to an expected compared to an unexpected stimulus is exclusively suppressed (lower summed response in Figure S2A) for local models. Indeed, population sharpening (remote modulations) can only result in expectation *enhancement* in this model definition. This is not only the case in the depicted example in figure S2A, but true for any combination of expected and unexpected stimuli; i.e., for remote models in S2A, the summed response to the unexpected stimulus will always be lower than to the expected stimulus, precisely because the remote modulation is affecting neural populations tuned away from the expected stimulus. In fact, the only case in which sharpening, or any other remote model, under this alternative implementation can account for expectation suppression, is for expected compared to expectation-free stimuli; i.e., stimuli for which no prediction is instantiated. Indeed, for the specific case of expectation-free stimuli this alternative implementation is identical to our implementation. That said, the vast majority of studies exploring expectation suppression contrast

unexpected with expected stimuli, thus requiring a model that can account for expectation suppression of expected relative to unexpected (not only expectation-free) stimuli. Thus, we considered these alternative model implementations of little relevance in arbitrating between accounts underlying expectation suppression, because only local modulations (dampening) can reliably result in the phenomenon of interest (expectation suppression), thereby categorically ruling out sharpening, and any other remote or global model.

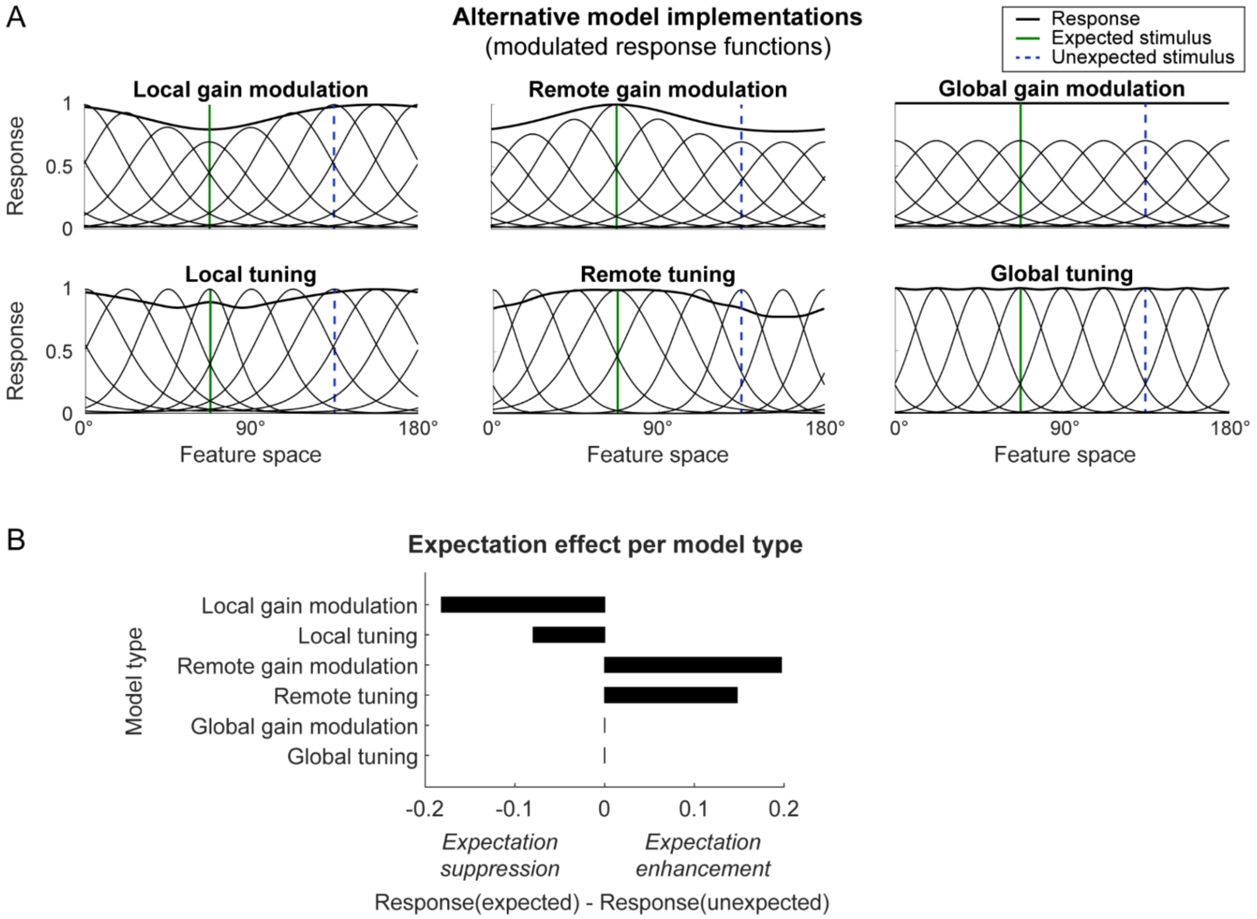

S2 Fig. Alternative model formulation.

A, Depicts the modulated neural response functions, with an example of the six modulation models. Thin black lines denote individual neural response functions across feature space, while thick lines indicated the summed response (normalized to one). There is no distinction between expected and unexpected stimuli in terms of the response functions, because a modulation is not conditional on the stimulus being expected in this implementation, but rather always occurs based on the expectation elicited by the leading image. Green shows the position of an expected stimulus in feature space (e.g., ~75 degrees orientation), according to which the responses are modulated. Blue dashed line shows a possible unexpected stimulus in feature space. **B,** Shows the expectation effect (expectation suppression or enhancement) in response to the expected (solid green line) and unexpected (dashed blue line)

stimulus shown in A. The contrast expected – unexpected is shown; i.e., negative values indicate expectation suppression, positive values expectation enhancement. As evident, only local models result in expectation suppression in this model implementation. Global models do not result in amplitude differences and remote models result in expectation enhancement. Notice, based on the summed response in A (thick line) that, the results in B are not dependent on the chosen unexpected or expected stimulus, but qualitatively would remain identical for each chosen stimulus. For example, for each position in feature space (stimulus) remote gain modulations would result in expectation enhancement, an increased response to expected (green solid line) compared to unexpected (blue dashed line) stimuli. In other words, sharpening accounts, and in fact any remote model, under this implementation cannot result in expectation suppression; similarly, global models cannot reliably produce expectation suppression either.

S3 Text. Best parameter values per ROI

To further explore the local gain modulations, we investigated which parameter values resulted in the best model fits. Of particular interest was the *a* (suppression magnitude) parameter value, indexing how strong neural gain is suppressed by expectations. To this end S3 Fig., shows histograms of the *a* parameter value distribution for the best 2% of local gain modulation models in the three ROIs. Additionally, the distribution for the *b* parameter is also depicted. The median *b* parameter was 1.1 in V1 and LOC, and 1.3 in TOFC. Given the possible range 0.1 to 2.3, these median values may imply a comparably broad spread of suppression across neuronal tuning. See *Materials and Methods: Simulation: Modulation by expectations* for details and Figure 9 for a depiction of the effect of the *b* parameter. However, the distribution of *b* parameter values itself was broad, preventing strong conclusions on the locality of neural suppression. The median *a* parameter was 0.85 in V1, 0.75 in LOC and 0.7 in TOFC, with a narrow distribution. On average (mean over ROIs) the optimal *a* parameter value was approximately 0.77, corresponding to a suppression of neural responses to approximately 77% of its unsuppressed response if a stimulus was expected. Interestingly, this number roughly matched the magnitude of suppression reported in terms of single and multi-unit firing rates recorded in inferior temporal cortex (IT) of non-human primates (figure 2A in [18] and figure 6A/B in [52]), suggesting a possible convergence on similar suppression magnitudes measured by different recording modalities. However, given the great number of differences between modalities, no formal link can be established.

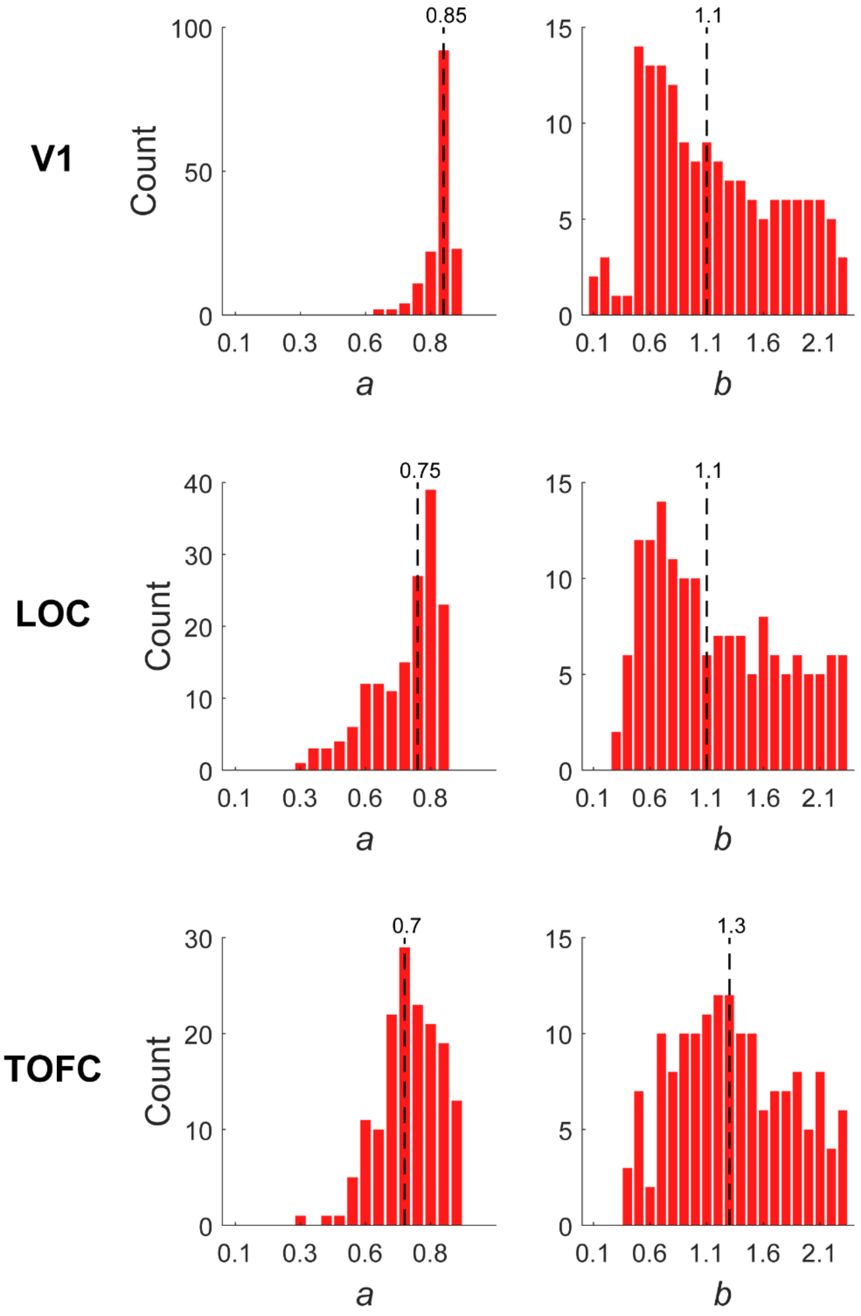

S3 Fig. Best parameter values.

Histograms of parameter values associated with the best 2% of models for local gain modulations in V1 (top), LOC (middle), and TOFC (bottom). Suppression magnitudes (*a*) are shown on the left and distance function (*b*) parameters on the right. Black dashed vertical lines indicate the median of the distribution. Depicted are the count of specific parameter values among the best models. The *a* parameter (left) reflects the magnitude of suppression. On average, across the three ROIs, an *a* parameter value of approximately 0.77 best explained the results, suggesting that response rates of neurons were reduced to ~77% of their unsuppressed response by perceptual expectations.

S4 Text. Ruling out pair-coding

We aim to explore the suppression of neural responses by valid predictions. However, an alternative explanation for expectation suppression is that expected stimulus pairs become associated over time. A consequence of this associative connection might be that the neural representations of paired stimuli become activated as a union, possibly by the presentation of the leading stimulus alone. As expected pairs are shown more frequently than unexpected pairs, neural responses may adapt more to paired stimuli than non-paired stimuli, hence accounting for the apparent expectation suppression by pair presentation frequency. On this account, expectation suppression would reflect a pair adaptation response rather than an effect of prediction. This account makes the testable prediction that neural representations of two stimuli forming a pair should be more similar to one another, reflecting the activation of the coded pair, than representations of non-paired stimuli. We addressed this prediction by comparing the representational dissimilarity between paired and non-paired stimuli during an independent object localizer run. The localizer run was performed at the end of the experiment reported in Richter et al. [17], and consisted of repeated presentations of the object stimuli a participant saw during the main experiment. Stimuli were shown in a random order; each individual stimulus shown for 12 seconds flashing at 2Hz.

First, we calculated in each ROI (V1, LOC, TOFC) the representational dissimilarity between all images by correlating each image's neural representation with the representation of each other image (dissimilarity = correlation – 1). We then averaged the dissimilarity metrics separately for paired (expected) and non-paired (unexpected) image pairs for each participant. Finally, across participants we submitted these dissimilarity metrics to a Bayesian paired t-test, performed using default settings in JASP 0.11.1.0.

Results showed that neural representations did *not* differ between paired and non-paired stimuli in any of our ROIs (V1: $BF_{10} = 0.223$; LOC: $BF_{10} = 0.232$; TOFC: $BF_{10} = 0.245$), with Bayes Factors showing moderate support for the null hypothesis (no difference). Thus, images of expected stimulus pairs were represented equally similar to one another than stimuli that were not usually associated (unexpected pairs). Therefore, it is unlikely that pair-coding can account for expectation suppression in the present data.

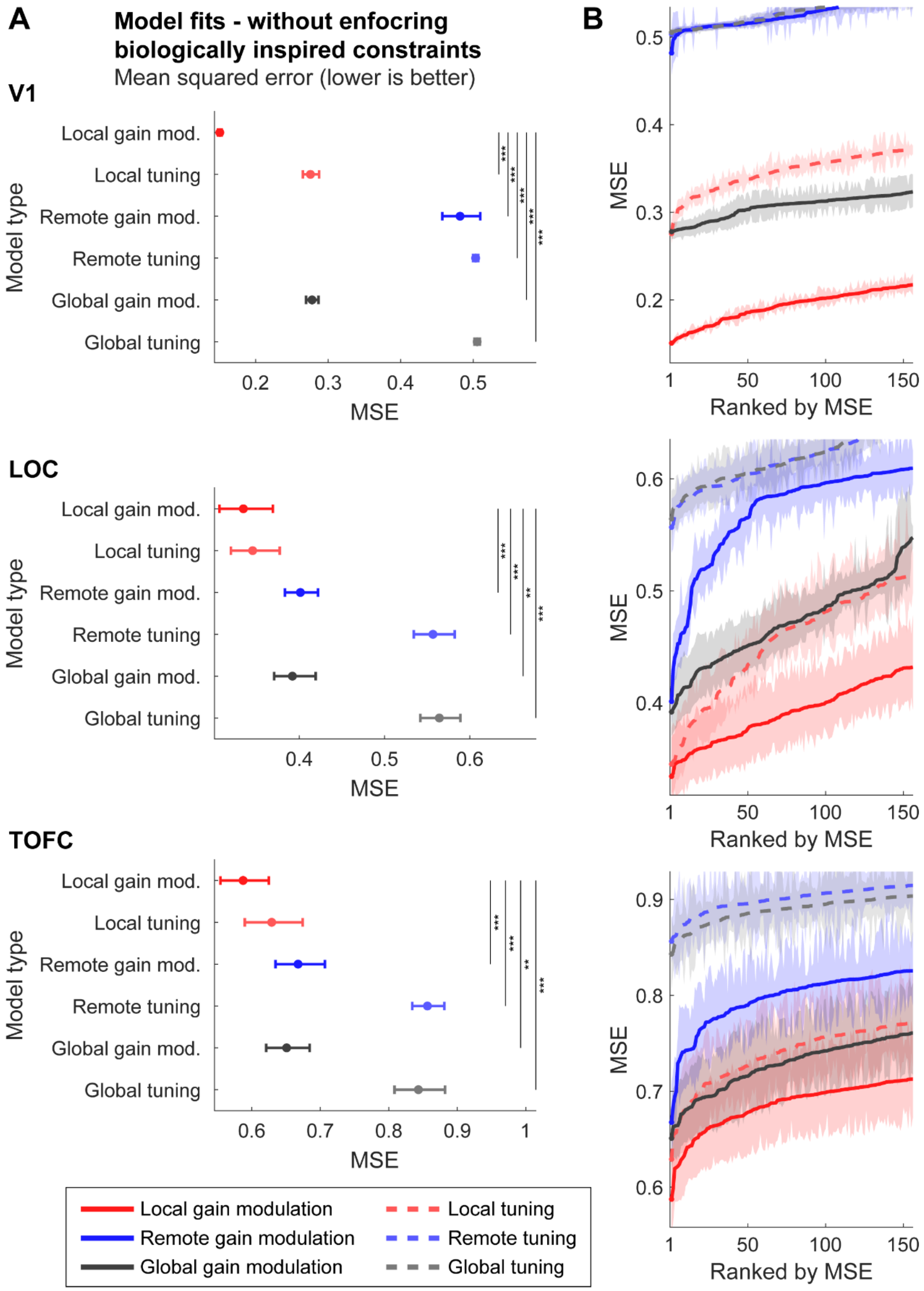

S4 Fig. Control analysis of model fits without enforcing biologically inspired constrains for neural response spaces.

Displayed are model fits in terms of mean squared error (MSE) in the three ROIs (V1, LOC, TOFC) for all six model types, identical to the analysis results depicted in Figure 5, except that no neural response space rejections were applied. Thus, all parameter combinations were assessed – for more details on neural response space rejection see:

Materials and Methods, Simulation, Response requirements. Results are qualitatively similar to those of the main analysis in Figure 5, with minor differences in best model fits in TOFC (local gain modulation) and slightly better overall fits (lower MSEs). In brief, in **A**, are depicted the MSE of the best fitting parameterization for each model type. In V1 (upper panel) local gain modulation outperformed all competing models as evident by a significantly lower MSE than any other model. In LOC (middle panel) and TOFC (lower panel), local gain modulation performed better than all other model types and crucially, better than the remote gain modulation model. Unlike in V1, the local tuning model fit the data similarly well as the local gain modulation. Error bars indicate 95% bootstrapped confidence intervals, bootstrapped across modelled participants. **B**, Shows the model fit (MSE) across multiple parameterizations (top 2%; 156 parameterizations) for each model type, indicating the robustness of the model to changes in parameterization. In V1 (upper panel), local gain modulation outperformed all competing models as seen by a significantly lower MSE across all displayed parameterizations. Shaded error bars indicate 95% bootstrapped confidence intervals. In LOC (middle panel) and TOFC (bottom panel), local gain modulation reliably outperformed the remote models across all depicted parameterizations and all model types across most parameterizations. However, while numerically lower, some of the best model fits for local gain modulation did not significantly differ from local tuning models. In all ROIs the average performance of the local gain modulation models was reliably better than any competing model, as indexed by the mean MSE across the top 2% of models. In sum, local gain modulations, representing dampening models, performed significantly better than any other model type on all metrics in V1 and across most parametrizations in LOC and TOFC.

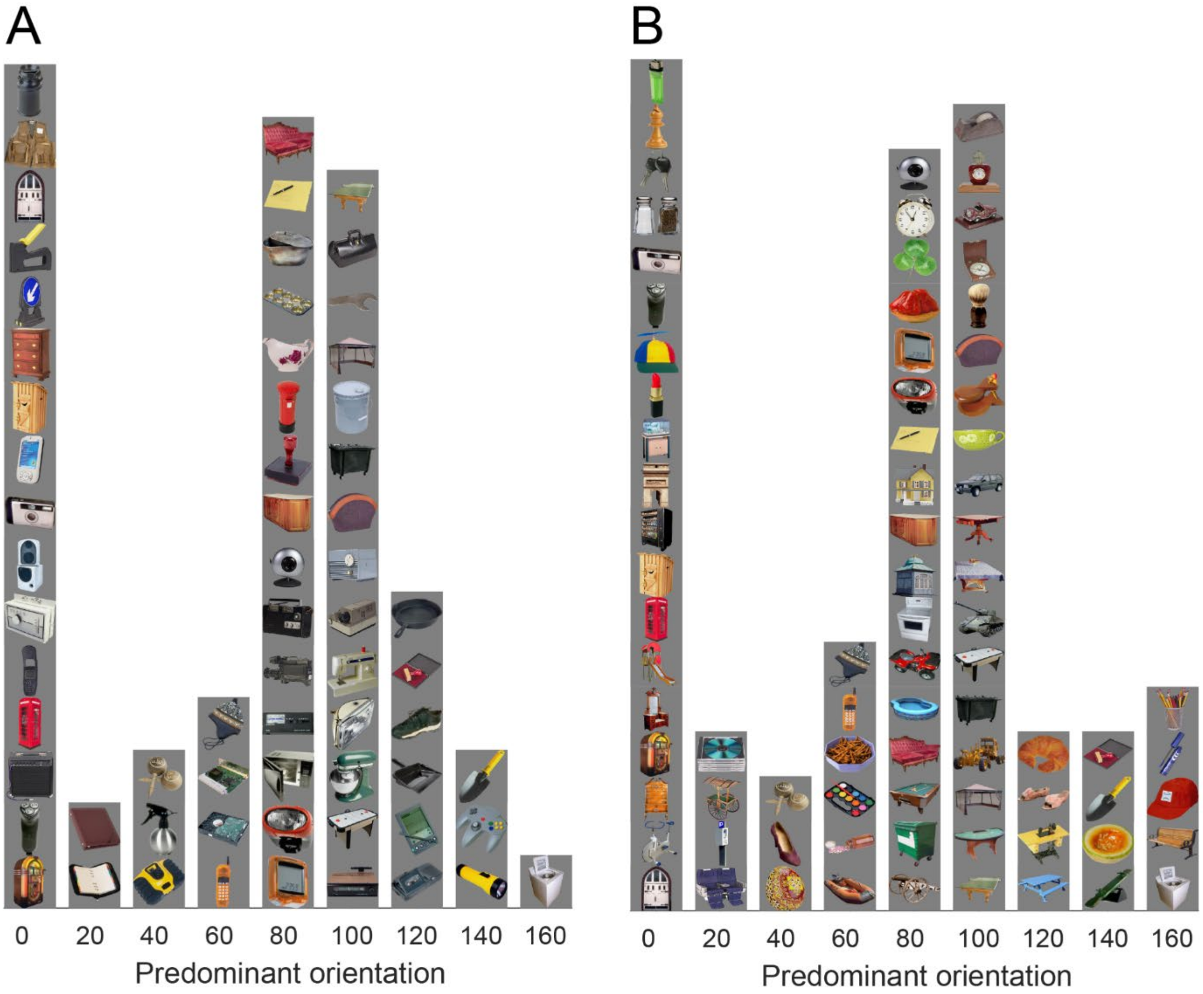

S5 Fig. Stimuli in orientation feature space (V1).

Utilized object stimuli arranged by their predominant orientation in steps of 20 degrees. **A**, Stimuli from Richter and de Lange [26]. **B**, Stimuli from Richter et al. [17].

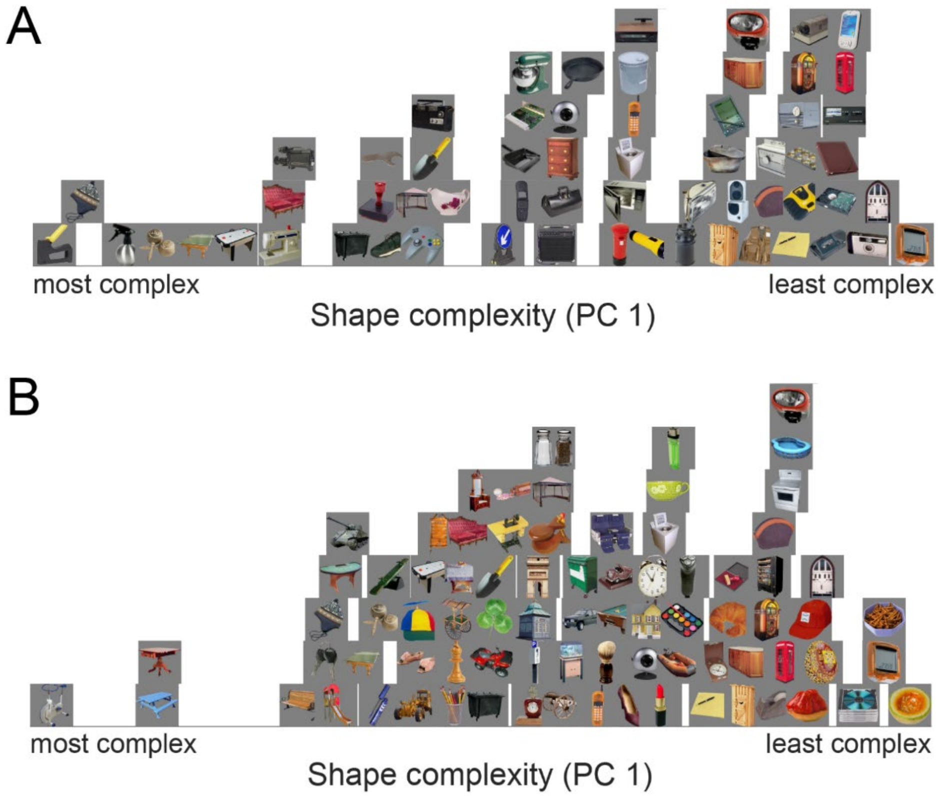

S6 Fig. Stimuli in shape complexity feature space (LOC).

Object stimuli arranged by shape complexity; first principle component (PC 1) on shape complexity measures. The most complex stimuli (e.g., irregular shapes with many protrusions) are displayed on the left, while the least complex objects (simple, squared, or circular objects) are found on the right. Horizontal distances between stimuli represent their shape dissimilarity (i.e., objects close together are similar in shape complexity). Stimuli are stacked vertically only for display purposes. **A**, Stimuli from Richter and de Lange [26]. **B**, Stimuli from Richter et al. [17].

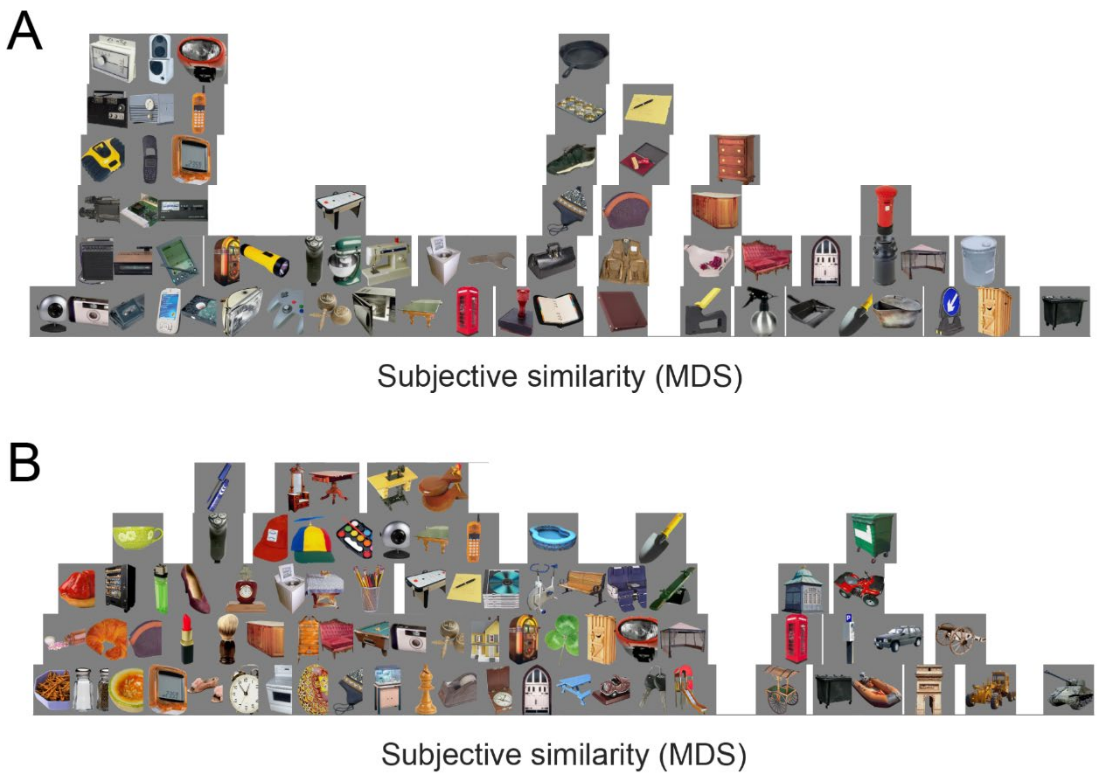

S7 Fig. Stimuli in semantic similarity feature space (TOFC).

Object stimuli arranged by semantic similarity (after multidimensional scaling; MDS), based on human ratings. For example, note that vehicles on the right in panel **B** are clustered together, in close proximity to structures and other objects found outside, but very distant to food and kitchen items on the left side. Distances on the horizontal axis represent the semantic dissimilarity (i.e., similar object are close together). Stimuli are stacked vertically for display purposes. **A**, Stimuli from Richter and de Lange [26]. **B**, Stimuli from Richter et al. [17].

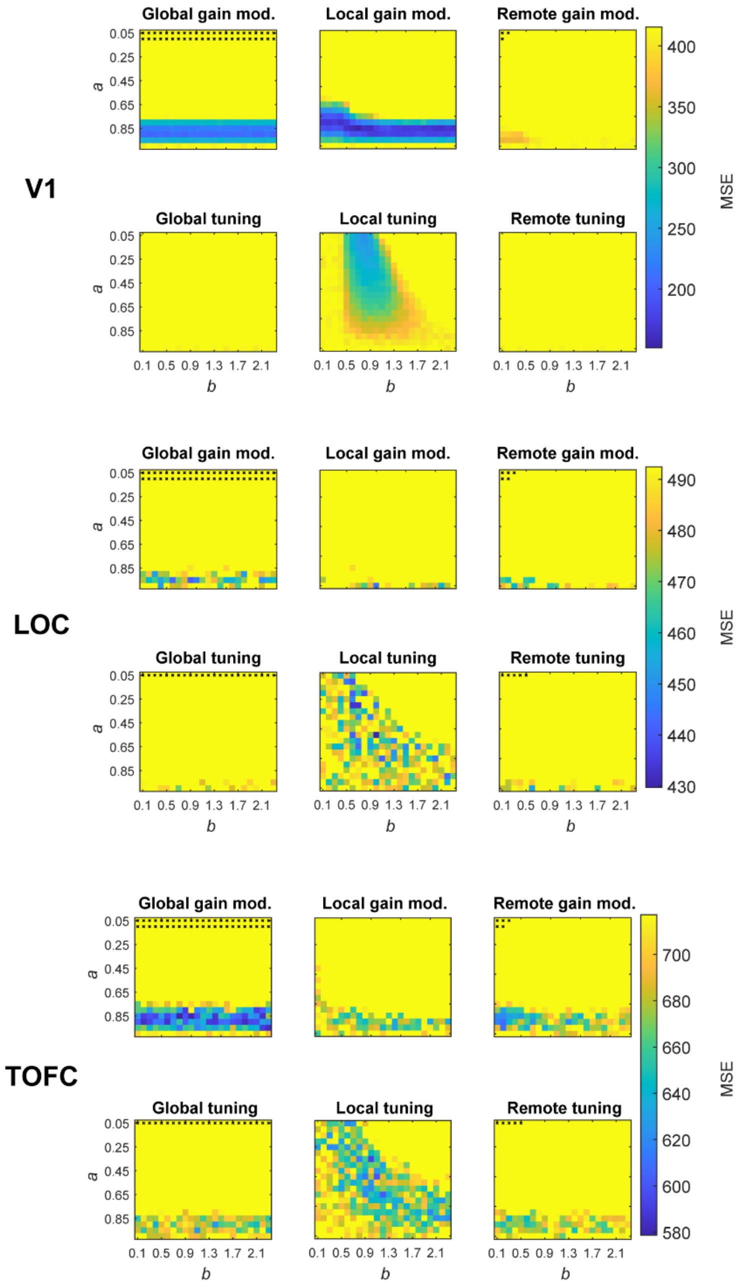

S8 Fig. MSE across parameter grid of parameters *a* and *b*.

Shown are median MSEs, averaged over σ, per model type and ROI (V1 top, LOC middle, TOFC bottom). For each model type the median MSE for all parameter combinations of *a* and *b* are shown. Colors are thresholded such that the median MSE of *a* = 1 (i.e., no suppression by expectation) is yellow, and the lowest MSE is dark blue. To explore the full parameter space, we did not apply any constraints on the response spaces for this analysis, unlike in the primary data analysis. Thus, here we also allowed parameter combinations that were considered biologically implausible (also see: *Response requirements* in *Materials and Methods*). Such implausible response spaces are marked as cells with a black x and were not considered in the main results. Results without such constraints are

depicted in S4 Fig. The error landscape for each model type and ROI appears well sampled and well contains the respective minima within the sampled range, unless the parameter was bound by theoretical reasons, indicating a good sampled parameter space.

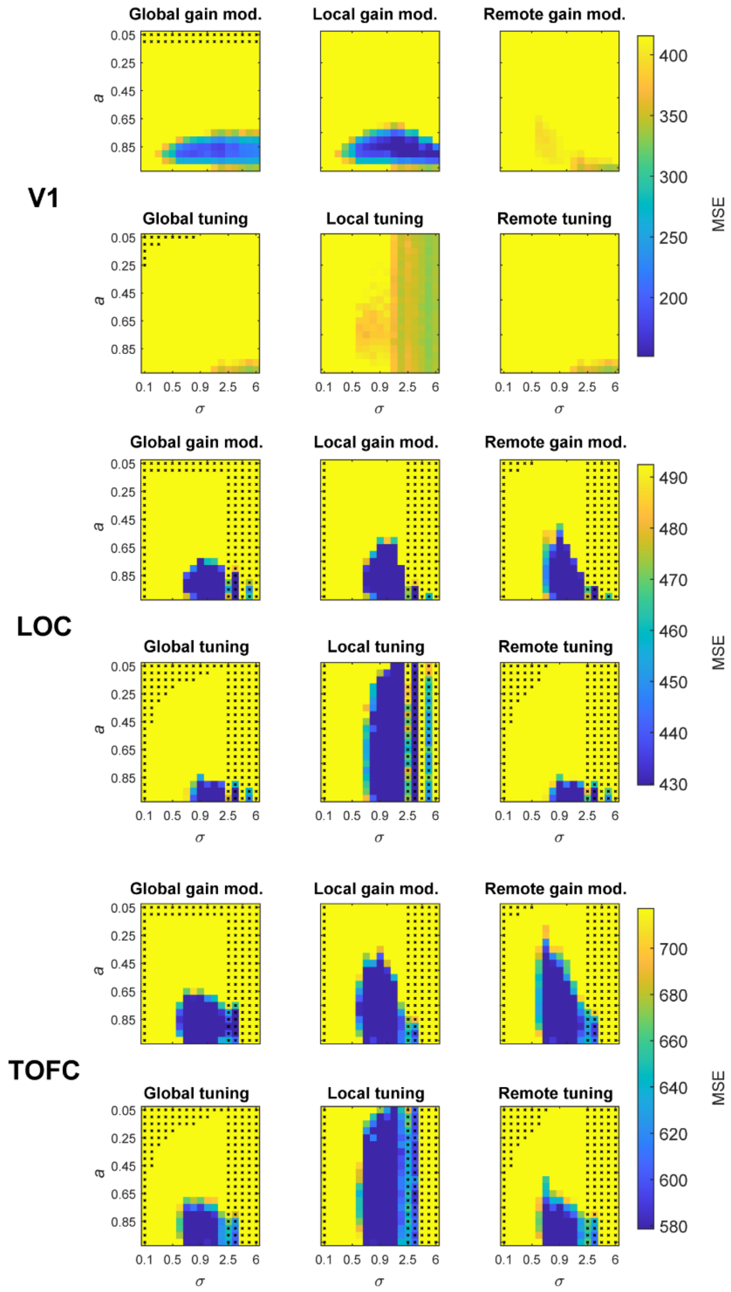

S9 Fig. MSE across parameter grid of parameters *a* and σ

Shown are median MSEs, averaged over *b*, per model type and ROI (V1 top, LOC middle, TOFC bottom). For each model type the median MSE for all parameter combinations of *a* and σ are shown. Colors are thresholded such that the median MSE of *a* = 1 (i.e., no suppression by expectation; in *a*, *b* parameter space) is yellow, and the lowest MSE is dark blue. To explore the full parameter space, we did not apply any constraints on the response spaces for this analysis, unlike in the primary data analysis. Thus, here we also allowed parameter combinations that were considered biologically implausible (also see: *Response requirements* in *Materials and Methods*). Such implausible response spaces are marked as cells with a black x and were not considered in the main results. Results without such

constraints are depicted in S4 Fig. Results show that the change of MSE values, particularly for low MSE values, is smooth, suggesting that parameter space has been well sampled in all ROIs.

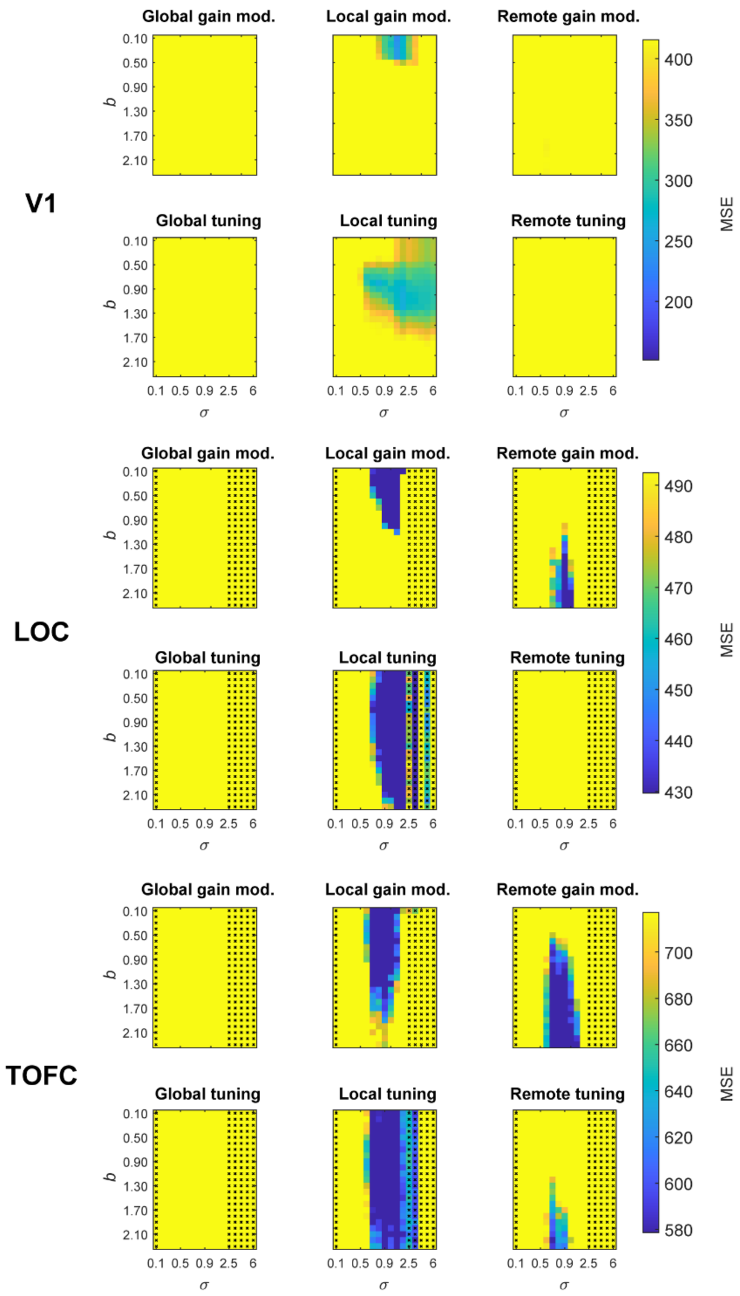

S10 Fig. MSE across parameter grid of parameters *b* and σ

Shown are median MSEs, averaged over *a*, per model type and ROI (V1 top, LOC middle, TOFC, bottom). For each model type the median MSE for all parameter combinations of *b* and σ are shown. Colors are thresholded such that the median MSE of *a* = 1 (i.e., no suppression by expectation; in *a*, *b* parameter space) is yellow, and the lowest MSE is dark blue. To explore the full parameter space, we did not apply any constraints on the response spaces for this analysis, unlike in the primary data analysis. Thus, here we also allowed parameter combinations that were considered biologically implausible (also see: *Response requirements* in *Materials and Methods*). Such implausible response spaces are marked as cells with a black x and were not considered in the main results. Results without such

constraints are depicted in S4 Fig. Results show that the change of MSEs, particularly for low MSE values, is smooth, suggesting that parameter space has been well sampled in all ROIs.

S1 Table. Statistics from empirical fMRI data analysis in V1

	Statistic			
**Outcome metric**	Intercept	Slope	t-statistic	p value
MAM	3.352	-0.200	-4.63	2.3e-05
WC	0.469	-0.056	-4.79	1.3e-05
BC	-0.076	0.009	4.69	1.8e-05
CP	0.504	-0.054	-4.77	1.4e-05
AMA	0.047	0.027	2.87	0.0059
AMS	0.056	0.025	2.57	0.0013
IP	0.081	0.028	1.85	0.0701

Shown are the results of the analysis of fMRI data from V1 per outcome metric (*MAM* = mean amplitude modulation, *WC* = within-class correlation, *BC* = between-class correlation, *CP* = classification performance (BC-WC), *AMA* = amplitude modulation by amplitude, *AMS* = amplitude modulation by selectivity, *IP* = image preference analysis; see *fMRI outcome metrics* for details). For each outcome metric the intercept and slope are reported, as well as the associated t-statistic and p value.

S2 Table. Statistics from empirical fMRI data analysis in LOC

	Statistic			
**Outcome metric**	Intercept	Slope	t-statistic	p value
MAM	4.031	-0.218	-5.15	3.6e-06
WC	0.147	-0.006	-0.66	0.5125
BC	-0.025	0.001	0.75	0.4572
CP	0.170	-0.007	-0.67	0.5081
AMA	-0.029	0.046	4.10	0.0001
AMS	-0.096	0.058	4.39	5.2e-05
IP	0.178	0.004	0.35	0.7307

Shown are the results of the analysis of fMRI data from LOC per outcome metric (*MAM* = mean amplitude modulation, *WC* = within-class correlation, *BC* = between-class correlation, *CP* = classification performance (BC-WC), *AMA* = amplitude modulation by amplitude, *AMS* = amplitude modulation by selectivity, *IP* = image preference analysis; see *fMRI outcome metrics* for details). For each outcome metric the intercept and slope are reported, as well as the associated t-statistic and p value.

S3 Table. Statistics from empirical fMRI data analysis in TOFC

	**Statistic**			
**Outcome metric**	Intercept	Slope	t-statistic	p value
MAM	2.259	-0.240	-6.96	4.3e-09
WC	0.066	-0.014	-2.05	0.0448
BC	-0.011	0.003	2.02	0.0487
CP	0.077	-0.017	-2.06	0.0442
AMA	-0.049	0.053	6.02	1.5e-07
AMS	-0.066	0.057	6.24	6.6e-08
IP	0.155	0.020	2.24	0.0292

Shown are the results of the analysis of fMRI data from TOFC per outcome metric (outcome metric (*MAM* = mean amplitude modulation, *WC* = within-class correlation, *BC* = between-class correlation, *CP* = classification performance (BC-WC), *AMA* = amplitude modulation by amplitude, *AMS* = amplitude modulation by selectivity, *IP* = image preference analysis; see *fMRI outcome metrics* for details). For each outcome metric the intercept and slope are reported, as well as the associated t-statistic and p value.

S4 Table. Feature spaces explain neural variance in their target ROIs

	**ROI**		
**Feature space**	**V1**	**LOC**	**TOFC**
Orientation energy	$t_{(55)}$ = 6.43, p = 3.3e-8, d_z = 0.86	$t_{(55)}$ = 2.52, p = 0.015, d_z = 0.34	$t_{(55)}$ = -1.23, p = 0.223, d_z = -0.16
Shape complexity	$t_{(55)}$ = -0.62, p = 0.540, d_z = -0.08	$t_{(55)}$ = 5.06, p = 5.1e-6, d_z = 0.68	$t_{(55)}$ = 2.96, p = 0.005, d_z = 0.40
Semantic similarity	$t_{(55)}$ = 0.20, p = 0.841, d_z = 0.03	$t_{(55)}$ = 3.90, p = 2.6e-4, d_z = 0.52	$t_{(55)}$ = 3.27, p = 0.002, d_z = 0.44

Shown are the results of one-sample t-tests, and associated effect sizes (Cohen's d), comparing the obtained partial correlation coefficients of neural and model RDM, while controlling for the other two model RDMs, against zero. Results show that the designed feature spaces explain significant unique neural variance in their target ROI (gray cells; V1 = orientation energy; LOC = shape complexity; TOFC = semantic similarity).

S5 Table. Contrasting explained neural variance of feature space models within ROIs

	**ROI**		
**Feature space**	**V1**	**LOC**	**TOFC**
Orientation energy vs Shape complexity	$t_{(55)} = 6.54$, $p = 2.1e\text{-}8$, $d_z = 0.88$	$t_{(55)} = -3.23$, $p = 0.002$, $d_z = -0.44$	$t_{(55)} = -3.03$, $p = 0.004$, $d_z = -0.40$
Orientation energy vs Semantic similar.	$t_{(55)} = 5.69$, $p = 5.1e\text{-}7$, $d_z = 0.73$	$t_{(55)} = -1.63$, $p = 0.110$, $d_z = -0.23$	$t_{(55)} = -3.37$, $p = 0.001$, $d_z = -0.44$
Shape complexity vs Semantic similar.	$t_{(55)} = -0.54$, $p = 0.592$, $d_z = -0.07$	$t_{(55)} = 1.80$, $p = 0.078$, $d_z = 0.25$	$t_{(55)} = -0.58$, $p = 0.566$, $d_z = -0.08$

Shown are the results of paired t-tests, and associated effect sizes (Cohen's d), comparing the obtained partial correlation coefficients of neural and model RDMs between different feature space models, while controlling for the influence of the other two model RDMs. Results show that the designed feature space in V1 (orientation energy) explains more unique neural variance than any other feature space model. In LOC the designated feature space, shape complexity, reliably outperforms the orientation energy feature space model. However, while numerically larger, it does not significantly explain more variance than the semantic feature space. Similarly, in TOFC semantic similarity significantly explains more neural variance than the orientation energy feature space model. Again, while numerically larger, it does not reliably explain more variance than the shape complexity feature space.
