# Supplementary material for: Dampened sensory representations for expected input across the ventral visual stream
Source: Oxf Open Neurosci. 2022 Aug 15;1:kvac013. doi: 10.1093/oons/kvac013 (PMC10939312; doi:10.1093/oons/kvac013)
Supplement: suppl_material_kvac013 [file suppl_material_kvac013.zip › Open peer review statements .docx]

Open peer review statements for OXFNSC-2021-002.R2 Dampened sensory representations for expected input across the ventral visual stream

Reviewer 1 – Minor revision

This manuscript describes simulation results of forward modelling of fMRI effects of expectation suppression from two previous studies. Though similar to a previous simulation study applying forward modelling to repetition suppression by Alink et al, the present study makes several important advancements, e.g, new data, new paradigm, new ROIs, better modelling of the feature space, quantitative fitting and tests of model recovery. The data and simulation code are provided for (relatively) easy access via the Donders website. I think it makes an excellent contribution to the literature, and have only minor comments (though I should declare that I was an author on the Alink et al paper, so obviously have a vested interest.)

1. I think it might help if the authors provided the winning model(s)’ best fit to the data properties in a format like Figure 1. For example, the real data for AMA and AMA show suggestion of a quadratic effect for all three ROIs – is this significant (rather than just a linear fit), and if so, does the winning model reproduce this effect (e.g, is it a grouping effect of endpoints)?

2. I wasn’t sure that comparing the best “a” values on page 18 to single-cell data was very convincing, because so many other variables are likely to differ, e.g, precise task, stimuli, measurement details, etc. And how “similar” is similar? I wonder whether this observation would be better in Supplementary Material, rather than at end of Results. Also, could the authors report the best “b” values as well, to see how “local” effects were?

3. While it is commendable that the authors went beyond Alink et al in assessing quantitative fits (at least to normalised slopes of the data properties), they could add to the limitations they mention in the Discussion (eg pages 24-25) that there are likely to be many other factors that affect those quantitative fits. For example, we know that the voxel-level noise is likely to be spatially-correlated across voxels, and such spatial correlation of noise affects properties like CP, yet the authors assume independent noise across voxels (unless I misunderstand what is stated on page 45). Furthermore, rather than a grid search, the authors could mention (for future work) that one could use iterative fitting techniques (based on a cost function like their MSE) to optimise the parameters. Indeed, one could even fit the full dataset, rather than the abstracted data properties, and include a model complexity term to penalise more flexible models (though on the latter point, the authors could make more of their model recovery, which suggests that no model was overly flexible).

4. While the three “biological plausibility criteria” seem defensible, it would be interesting to know whether the same results arise without them – e.g, are they important for only gain modulation to fit the data? Could the authors also make the white blocks on Figs S7-S9 transparent, so one could see whether there are additional local (or even global) minima within these regions?

5. I think it’s a bit unfair on Alink et al to say on page 21 that “The influence of noise on the utilized forward models has been demonstrated by Ramírez and Merriam [24] and could thus explain the difference in results as well”, since in Alink et al’s reply to Ramírez and Merriam, we did explore a wide range of noise levels and still found that only local scaling could capture the qualitative patterns in our Experiment 1. However, I admit that I do not have a ready explanation why the present authors found that other models could explain their data properties qualitatively, since those qualitative patterns were identical to those in our Experiment 1. Thus while I doubt this discrepancy owes to noise levels (or density/range of grid search for that matter), I accept it could owe to the more fine-grained feature space (or cropping of non-circular spaces, or even different statistical criteria for “fitting”?).

6. Do the authors have a theoretical reason for using the terms “dampening” and “gain modulation” rather than the “scaling” used by Alink et al? I appreciate that “dampening” might be more descriptive, or “gain modulation” might be more precise, but it might confuse the literature to have too many terms – could the authors at least make explicit somewhere in the paper that the terms are (or are not) equivalent?

7. When introducing the new, seventh data pattern of “IP” on page 7, a brief rationale could be added, e.g., summarising what is stated in Supplementary Material on page 34.

8. In Fig S1, why do the x-axis labels in Panel A and B have different ordering of models (and colours)?

9. Line 1293: “assed” = “assessed”

Reviewer 2 - Minor revision

Richter et al. explore the mechanisms underlying expectation suppression, a consistently reduced neural response to expected compared to unexpected stimuli. This down-regulation has been explained by two alternative accounts: first, expectations can filter out neural responses redundant with high-level predictions (“dampening” model). Second, expectations could suppress neural responses tuned away from the expected stimulus (“sharpening” model). However, previous studies inferred either dampened or sharpened responses at the neural level from voxel-level activity. Here, the authors set out to extend a forward modelling approach recently used to model a similar effect (adaptation suppression) to investigate expectation suppression across seven (uni- and multivariate) fMRI outcomes within three regions of interests in the ventral visual stream. Their modelling results are in line with a dampening account of expectations.

I was pleased to read this paper, which tries to answer a crucial question in a timely debate. Overall, I think the study is well-motivated, and its contribution should be of interest to a wide range of researchers. Moreover, the methods and the analytical approach are solid. I further appreciate that the authors fully share the data and analysis scripts employed. Thus, my overall impression is very positive. I only have a couple of points for clarification.

- I am primarily concerned by the fact that the data in this paper come from studies that originally showed a dampened response. Although the authors briefly mention this in the discussion, I wonder to what extent it limits the conclusions that can be drawn from the modelling approach, since as the authors describe, the specifics of the task and the stimuli frequently lead to either sharpening or dampening of responses. Thus, I would recommend toning down the conclusions and highlighting that the results apply to this experimental context in particular, and not necessarily to all instances of expectation suppression.

- Regarding the analysis plotted in Figure 2, I wonder if it would be clearer to compute partial correlations between the three models and the neural data. That way, you could test whether the relevant feature space model significantly explain a unique portion of variance, while controlling for the variance explained by the other two models.

Decision letter first revision

From:

sam.gilbert@ucl.ac.uk

To:

david.richter@donders.ru.nl

CC:

BCC:

sam.gilbert@ucl.ac.uk

Subject:

Oxford Open Neuroscience - Decision on Manuscript ID OXFNSC-2021-002

Body:

02-Mar-2022

Dear Dr. Richter,

Manuscript ID OXFNSC-2021-002 entitled "Dampened sensory representations for expected input across the ventral visual stream" which you submitted to the Oxford Open Neuroscience, has been reviewed. The comments of the reviewer(s) are included at the bottom of this letter.

The reviewer(s) have recommended publication, but also suggest some minor revisions to your manuscript. Therefore, I invite you to respond to the reviewer(s)' comments and revise your manuscript.

To revise your manuscript, log into https://mc.manuscriptcentral.com/oxfnsc and enter your Author Centre, where you will find your manuscript title listed under "Manuscripts with Decisions." Under "Actions," click on "Create a Revision." Your manuscript number has been appended to denote a revision.

You may also click the below link to start the revision process (or continue the process if you have already started your revision) for your manuscript. If you use the below link you will not be required to login to ScholarOne Manuscripts.

*** PLEASE NOTE: This is a two-step process. After clicking on the link, you will be directed to a webpage to confirm. ***

https://mc.manuscriptcentral.com/oxfnsc?URL_MASK=326f03922bfd4c229942b26073c51024

You will be unable to make your revisions on the originally submitted version of the manuscript. Instead, revise your manuscript using a word processing program and save it on your computer. Please also highlight the changes to your manuscript within the document by using the track changes mode in MS Word or by using bold or colored text.

Once the revised manuscript is prepared, you can upload it and submit it through your Author Centre.

When submitting your revised manuscript, you will be able to respond to the comments made by the reviewer(s) in the space provided. You can use this space to document any changes you make to the original manuscript. In order to expedite the processing of the revised manuscript, please be as specific as possible in your response to the reviewer(s).

IMPORTANT: Your original files are available to you when you upload your revised manuscript. Please delete any redundant files before completing the submission.

Because we are trying to facilitate timely publication of manuscripts submitted to the Oxford Open Neuroscience, your revised manuscript should be uploaded as soon as possible. If it is not possible for you to submit your revision in a reasonable amount of time, we may have to consider your paper as a new submission.

Once again, thank you for submitting your manuscript to the Oxford Open Neuroscience and I look forward to receiving your revision.

Sincerely,

Dr. Sam Gilbert

Senior Editor, Oxford Open Neuroscience

sam.gilbert@ucl.ac.uk

Reviewer: 1

Comments to the Author

This manuscript describes simulation results of forward modelling of fMRI effects of expectation suppression from two previous studies. Though similar to a previous simulation study applying forward modelling to repetition suppression by Alink et al, the present study makes several important advancements, e.g, new data, new paradigm, new ROIs, better modelling of the feature space, quantitative fitting and tests of model recovery. The data and simulation code are provided for (relatively) easy access via the Donders website. I think it makes an excellent contribution to the literature, and have only minor comments (though I should declare that I was an author on the Alink et al paper, so obviously have a vested interest.)

1. I think it might help if the authors provided the winning model(s)’ best fit to the data properties in a format like Figure 1. For example, the real data for AMA and AMA show suggestion of a quadratic effect for all three ROIs – is this significant (rather than just a linear fit), and if so, does the winning model reproduce this effect (e.g, is it a grouping effect of endpoints)?

2. I wasn’t sure that comparing the best “a” values on page 18 to single-cell data was very convincing, because so many other variables are likely to differ, e.g, precise task, stimuli, measurement details, etc. And how “similar” is similar? I wonder whether this observation would be better in Supplementary Material, rather than at end of Results. Also, could the authors report the best “b” values as well, to see how “local” effects were?

3. While it is commendable that the authors went beyond Alink et al in assessing quantitative fits (at least to normalised slopes of the data properties), they could add to the limitations they mention in the Discussion (eg pages 24-25) that there are likely to be many other factors that affect those quantitative fits. For example, we know that the voxel-level noise is likely to be spatially-correlated across voxels, and such spatial correlation of noise affects properties like CP, yet the authors assume independent noise across voxels (unless I misunderstand what is stated on page 45). Furthermore, rather than a grid search, the authors could mention (for future work) that one could use iterative fitting techniques (based on a cost function like their MSE) to optimise the parameters. Indeed, one could even fit the full dataset, rather than the abstracted data properties, and include a model complexity term to penalise more flexible models (though on the latter point, the authors could make more of their model recovery, which suggests that no model was overly flexible).

4. While the three “biological plausibility criteria” seem defensible, it would be interesting to know whether the same results arise without them – e.g, are they important for only gain modulation to fit the data? Could the authors also make the white blocks on Figs S7-S9 transparent, so one could see whether there are additional local (or even global) minima within these regions?

5. I think it’s a bit unfair on Alink et al to say on page 21 that “The influence of noise on the utilized forward models has been demonstrated by Ramírez and Merriam [24] and could thus explain the difference in results as well”, since in Alink et al’s reply to Ramírez and Merriam, we did explore a wide range of noise levels and still found that only local scaling could capture the qualitative patterns in our Experiment 1. However, I admit that I do not have a ready explanation why the present authors found that other models could explain their data properties qualitatively, since those qualitative patterns were identical to those in our Experiment 1. Thus while I doubt this discrepancy owes to noise levels (or density/range of grid search for that matter), I accept it could owe to the more fine-grained feature space (or cropping of non-circular spaces, or even different statistical criteria for “fitting”?).

6. Do the authors have a theoretical reason for using the terms “dampening” and “gain modulation” rather than the “scaling” used by Alink et al? I appreciate that “dampening” might be more descriptive, or “gain modulation” might be more precise, but it might confuse the literature to have too many terms – could the authors at least make explicit somewhere in the paper that the terms are (or are not) equivalent?

7. When introducing the new, seventh data pattern of “IP” on page 7, a brief rationale could be added, e.g., summarising what is stated in Supplementary Material on page 34.

8. In Fig S1, why do the x-axis labels in Panel A and B have different ordering of models (and colours)?

9. Line 1293: “assed” = “assessed”

Reviewer: 2

Comments to the Author

Richter et al. explore the mechanisms underlying expectation suppression, a consistently reduced neural response to expected compared to unexpected stimuli. This down-regulation has been explained by two alternative accounts: first, expectations can filter out neural responses redundant with high-level predictions (“dampening” model). Second, expectations could suppress neural responses tuned away from the expected stimulus (“sharpening” model). However, previous studies inferred either dampened or sharpened responses at the neural level from voxel-level activity. Here, the authors set out to extend a forward modelling approach recently used to model a similar effect (adaptation suppression) to investigate expectation suppression across seven (uni- and multivariate) fMRI outcomes within three regions of interests in the ventral visual stream. Their modelling results are in line with a dampening account of expectations.

I was pleased to read this paper, which tries to answer a crucial question in a timely debate. Overall, I think the study is well-motivated, and its contribution should be of interest to a wide range of researchers. Moreover, the methods and the analytical approach are solid. I further appreciate that the authors fully share the data and analysis scripts employed. Thus, my overall impression is very positive. I only have a couple of points for clarification.

- I am primarily concerned by the fact that the data in this paper come from studies that originally showed a dampened response. Although the authors briefly mention this in the discussion, I wonder to what extent it limits the conclusions that can be drawn from the modelling approach, since as the authors describe, the specifics of the task and the stimuli frequently lead to either sharpening or dampening of responses. Thus, I would recommend toning down the conclusions and highlighting that the results apply to this experimental context in particular, and not necessarily to all instances of expectation suppression.

- Regarding the analysis plotted in Figure 2, I wonder if it would be clearer to compute partial correlations between the three models and the neural data. That way, you could test whether the relevant feature space model significantly explain a unique portion of variance, while controlling for the variance explained by the other two models.

Associate Editor

Comments to the Author:

Dear Dr Richter,

Thank you for submitting this interesting manuscript. As you will see, both reviewers found your manuscript of interest in principle, but also raise a number of concerns that would be important to address in a revision.

In addition to the comments made by the reviewers, I would like to add a few points:

1. As noted by Reviewer 1, it would be good to show the final fits of the winning model in comparison to the data in an overview figure. This would help readers to take away the key message.

2. I would recommend to use less acronyms. Please revisit your acronyms throughout the paper and try to reduce them as much as possible.

3. Please provide an overview of the number of parameters per model in the main text

4. Please use the same axis scaling in Figure 5, such that readers can appreciate the differences in the overall level of fits

5. Following Reviewer 1’s suggestion, I would carefully word the possible implications to the comparison of the suppression parameters to animal studies.

Date Sent:

02-Mar-2022

Author response to first decision letter

Reply to reviewer comments

We thank the reviewers and editor for the positive evaluation of our manuscript and the excellent suggestions for improvements. Below we provide a point by point reply (in blue) to the individual comments. Additionally, we have marked all adjustments in the manuscript (in blue). We hope that the additional analyses and adjustments satisfy the reviewers concerns in full.

Reviewer 1

Comments to the Author

This manuscript describes simulation results of forward modelling of fMRI effects of expectation suppression from two previous studies. Though similar to a previous simulation study applying forward modelling to repetition suppression by Alink et al, the present study makes several important advancements, e.g, new data, new paradigm, new ROIs, better modelling of the feature space, quantitative fitting and tests of model recovery. The data and simulation code are provided for (relatively) easy access via the Donders website. I think it makes an excellent contribution to the literature, and have only minor comments (though I should declare that I was an author on the Alink et al paper, so obviously have a vested interest.)

1. I think it might help if the authors provided the winning model(s)’ best fit to the data properties in a format like Figure 1. For example, the real data for AMA and AMA show suggestion of a quadratic effect for all three ROIs – is this significant (rather than just a linear fit), and if so, does the winning model reproduce this effect (e.g, is it a grouping effect of endpoints)?

- We agree with the reviewer that providing the winning model’s best fit to the outcome metrics is a useful addition to our results. Thus, we now display in Figure 6 for each ROI the MRI data and the best model, using the same outcome metrics as in Figure 1. The associated description in the Results section reads:
“Finally, Figure 6 depicts the seven outcome metrics for both the empirical fMRI data and the best fitting model. Results confirmed that the sign of the modelled data matches the sign of the empirical outcome metrics. Moreover, for most metrics the fit of the slope coefficients was evident by visual inspection. However, some divergences were also noticeable. First, mean amplitude modulation magnitudes were systematically overestimated by the best forward models. Second, the slope coefficients of the amplitude modulation by amplitude and amplitude modulation by selectivity metrics were underestimated. Furthermore, the fMRI data may have followed a quadratic trend on these metrics, while the model data showed a linear increase. These results suggest that there was more variability in suppression magnitude between voxels in the empirical data than the modelled data, constituting avenues for improvements in voxel formation for future modelling work. Combined, these results demonstrate that while the modelled data fit the sign and slope reasonably well, further improvements in model fitting exist, particularly for metrics reflecting complex, possibly non-linear relationships across voxels.

**Figure 6. Fit of best models compared to empirical fMRI data.**

Depicted are the slopes and data for the best fitting forward model (green) and fMRI data (black) for the seven outcome metrics in each ROI. Data has been scaled (divided by the mean) for display purposes; for details on the forward modelling procedure see: *Martials and Methods, Simulation*. *Un.* = Unexpected trials, i.e., when the trailing image was unexpected given the leading image. *Ex*. = expected trials, i.e., when the trailing image was expected given the leading image. Outcome metrics: *MAM* = mean amplitude modulation (expectation suppression), *WC* = within-class correlation, *BC* = between-class correlation, *CP* = classification performance (BC-WC), *AMA* = amplitude modulation by amplitude, *AMS* = amplitude modulation by selectivity, *IP* = image preference analysis.”

2. I wasn’t sure that comparing the best “a” values on page 18 to single-cell data was very convincing, because so many other variables are likely to differ, e.g, precise task, stimuli, measurement details, etc. And how “similar” is similar? I wonder whether this observation would be better in *Supplementary Material*, rather than at end of Results. Also, could the authors report the best “b” values as well, to see how “local” effects were?

- We agree that our previous conclusion with respect to the parameter value comparison to results from electrophysiology was not sufficiently convincing to warrant inclusion in the main text. Thus, we moved the results and discussion to the supplemental material and toned down the associated conclusion. Additionally, as suggested by the reviewer, we now also explore and depict the best ‘b’ parameter values in the same supplemental material – S3 Text and S3 Fig.:

“**S3 Text. Best parameter values per ROI**
To further explore the local gain modulations, we investigated which parameter values resulted in the best model fits. Of particular interest was the *a* (suppression magnitude) parameter value, indexing how strong neural gain is suppressed by expectations. To this end

S3 Fig., shows histograms of the *a* parameter value distribution for the best 2% of local gain modulation models in the three ROIs. Additionally, the distribution for the *b* parameter is also depicted. The median *b* parameter was 1.1 in V1 and LOC, and 1.3 in TOFC. Given the possible range 0.1 to 2.3, these median values may imply a comparably broad spread of suppression across neuronal tuning. See *Materials and Methods: Simulation: Modulation by expectations* for details and Figure 9 for a depiction of the effect of the *b* parameter. However, the distribution of *b* parameter values itself was broad, preventing strong conclusions on the locality of neural suppression. The median *a* parameter was 0.85 in V1, 0.75 in LOC and 0.7 in TOFC, with a narrow distribution. On average (mean over ROIs) the optimal *a* parameter value was approximately 0.77, corresponding to a suppression of neural responses to approximately 77% of its unsuppressed response if a stimulus was expected. Interestingly, this number roughly matched the magnitude of suppression reported in terms of single and multi-unit firing rates recorded in inferior temporal cortex (IT) of non-human primates (figure 2A in [18] and figure 6A/B in [52]), suggesting a possible convergence on similar suppression magnitudes measured by different recording modalities. However, given the great number of differences between modalities, no formal link can be established.

S3 Fig. Best parameter values.

Histograms of parameter values associated with the best 2% of models for local gain modulations in V1 (top), LOC (middle), and TOFC (bottom). Suppression magnitudes (*a*) are shown on the left and distance function (*b*) parameters on the right. Black dashed vertical lines indicate the median of the

distribution. Depicted are the count of specific parameter values among the best models. The *a* parameter (left) reflects the magnitude of suppression. On average, across the three ROIs, an *a* parameter value of approximately 0.77 best explained the results, suggesting that response rates of neurons were reduced to ~77% of their unsuppressed response by perceptual expectations.”

3. While it is commendable that the authors went beyond Alink et al in assessing quantitative fits (at least to normalised slopes of the data properties), they could add to the limitations they mention in the Discussion (eg pages 24-25) that there are likely to be many other factors that affect those quantitative fits. For example, we know that the voxel-level noise is likely to be spatially-correlated across voxels, and such spatial correlation of noise affects properties like CP, yet the authors assume independent noise across voxels (unless I misunderstand what is stated on page 45).

Furthermore, rather than a grid search, the authors could mention (for future work) that one could use iterative fitting techniques (based on a cost function like their MSE) to optimise the parameters. Indeed, one could even fit the full dataset, rather than the abstracted data properties, and include a model complexity term to penalise more flexible models (though on the latter point, the authors could make more of their model recovery, which suggests that no model was overly flexible).

- We agree with the reviewer that explicitly stating limitations of our current approach and suggesting future improvements for similar studies is a valuable addition. Accordingly, we added a separate section to the discussion of the limitations on page 28:
“**Limitations of the fitting procedure**

In addition to the above outlined limitations, we acknowledge that the here utilized procedure for determining the best fitting model has inherent limitations. For example, it is known that noise across voxels in fMRI data is spatially correlated. Such spatial correlations are not accounted for in our present modelling procedure, thus constituting a divergence between the forward models and the fMRI data, likely impacting the fit of multivariate outcome metrics (e.g. the classification performance). Future work could improve on this by including spatially correlated noise, informed by empirical fMRI data. Moreover, iterative model fitting methods instead of a grid search could be used in future work to further improve the here utilized method. However, we also highlight that using a grid search allows a systematic assessment of the influence of the parameter values on the model fit – for details see: Supplemental Figures S8-S10 Fig.”

4. While the three “biological plausibility criteria” seem defensible, it would be interesting to know whether the same *results arise without them* – e.g, are they important for only gain modulation to fit the data? Could the authors also make the white blocks on Figs S7-S9 transparent, so one could see whether there are additional local (or even global) minima within these regions?

- We think that providing additional transparency by displaying the results also for ‘implausible’ parameter combinations is a valuable addition. Accordingly, we performed the forward modelling again, but without applying any ‘biological plausibility criteria’. The results of this control analysis are now displayed in Supplemental Figure S4. In brief, results largely mirror those of the main analysis with biological plausibility criteria applied. Differences are: (1) slightly superior fits overall, evident as smaller MSEs and (2) a change of the numerically best fitting model type in TOFC, now also being local gain modulation as in the other ROIs (unlike in the main results, local tuning). Thus, overall conclusions are unchanged, and in favour of local gain modulation underlying expectation suppression, without applying biological plausibility criteria. However, we do believe that constraining the main analysis to plausible neural response spaces is sensible, as it does ensure the reader that any winning model cannot contain highly implausible neural responses, without the need to inspect specific parameter combinations.
We added a short paragraph to the Results section highlighting this additional control analysis:
“To ensure that successful models did not significantly violate known characteristics of

neural responses, we constrained the forward models using biologically inspired criteria. In brief, we excluded models which (1) contained blind-spots for feature values, (2) suppression profiles exceeding the average response difference of visual stimulation compared to baseline, (3) a lack of feature tuning; for details see: *Materials and Methods, Simulation, Response requirements*. However, to demonstrate that the above presented results did not depend on this constraint we repeated the simulation without enforcing these criteria in a control analysis. Results, depicted in S4 Fig. confirmed that local gain modulations best explained expectation suppression across all ROIs.”

- Furthermore, the corresponding S4 Figure and figure text are now added to the supplemental material:

“S4 Fig. Control analysis of model fits without enforcing biologically inspired constrains for neural response spaces.
Displayed are model fits in terms of mean squared error (MSE) in the three ROIs (V1, LOC, TOFC) for all six model types, identical to the analysis results depicted in Figure 5, except that no neural response space rejections were applied. Thus, all parameter combinations were assessed – for more details on neural response space rejection see: *Materials and Methods, Simulation, Response requirements*. Results are qualitatively similar to those of the main analysis in Figure 5, with minor differences in best model fits in TOFC (local gain modulation) and slightly better overall fits (lower

MSEs). In brief, in **A**, are depicted the MSE of the best fitting parameterization for each model type. In V1 (upper panel) local gain modulation outperformed all competing models as evident by a significantly lower MSE than any other model. In LOC (middle panel) and TOFC (lower panel), local gain modulation performed better than all other model types and crucially, better than the remote gain modulation model. Unlike in V1, the local tuning model fit the data similarly well as the local gain modulation. Error bars indicate 95% bootstrapped confidence intervals, bootsrapped across modelled participants. **B**, Shows the model fit (MSE) across multiple parameterizations (top 2%; 156 parameterizations) for each model type, indicating the robustness of the model to changes in parameterization. In V1 (upper panel), local gain modulation outperformed all competing models as seen by a significantly lower MSE across all displayed parameterizations. Shaded error bars indicate 95% bootstrapped confidence intervals. In LOC (middle panel) and TOFC (bottom panel), local gain modulation reliably outperformed the remote models across all depicted parameterizations and all model types across most parameterizations. However, while numerically lower, some of the best model fits for local gain modulation did not significantly differ from local tuning models. In all ROIs the average performance of the local gain modulation models was reliably better than any competing model, as indexed by the mean MSE across the top 2% of models. In sum, local gain modulations, representing dampening models, performed significantly better than any other model type on all metrics in V1 and across most parametrizations in LOC and TOFC.”

- For more transparency of the results, and as suggested by the reviewer, we now also depict the MSEs across the parameter grid in S8-S10 Figs. without applying the biological plausibility criteria but marked any rejected response spaces.

S8 Fig. MSE across parameter grid of parameters *a* and *b*.
Shown are median MSEs, averaged over σ, per model type and ROI (V1 top, LOC middle, TOFC

bottom). For each model type the median MSE for all parameter combinations of *a* and *b* are shown. Colors are thresholded such that the median MSE of *a* = 1 (i.e., no suppression by expectation) is yellow, and the lowest MSE is dark blue. To explore the full parameter space, we did not apply any constraints on the response spaces for this analysis, unlike in the primary data analysis. Thus, here we also allowed parameter combinations that were considered biologically implausible (also see: *Response requirements* in *Materials and Methods*). Such implausible response spaces are marked as cells with a black x and were not considered in the main results. Results without such constraints are depicted in S4 Fig. The error landscape for each model type and ROI appears well sampled and well contains the respective minima within the sampled range, unless the parameter was bound by theoretical reasons, indicating a good sampled parameter space.

S9 Fig. MSE across parameter grid of parameters *a* and σ
Shown are median MSEs, averaged over *b*, per model type and ROI (V1 top, LOC middle, TOFC bottom). For each model type the median MSE for all parameter combinations of *a* and σ are shown. Colors are thresholded such that the median MSE of *a* = 1 (i.e., no suppression by expectation; in *a*, *b* parameter space) is yellow, and the lowest MSE is dark blue. To explore the full parameter space, we did not apply any constraints on the response spaces for this analysis, unlike in the primary data analysis. Thus, here we also allowed parameter combinations that were considered biologically implausible (also see: *Response requirements* in *Materials and Methods*). Such implausible response spaces are marked as cells with a black x and were not considered in the main results. Results without

such constraints are depicted in S4 Fig. Results show that the change of MSE values, particularly for low MSE values, is smooth, suggesting that parameter space has been well sampled in all ROIs.

S10 Fig. MSE across parameter grid of parameters *b* and σ
Shown are median MSEs, averaged over *a*, per model type and ROI (V1 top, LOC middle, TOFC, bottom). For each model type the median MSE for all parameter combinations of *b* and σ are shown. Colors are thresholded such that the median MSE of *a* = 1 (i.e., no suppression by expectation; in *a*, *b* parameter space) is yellow, and the lowest MSE is dark blue. To explore the full parameter space, we did not apply any constraints on the response spaces for this analysis, unlike in the primary data analysis. Thus, here we also allowed parameter combinations that were considered biologically implausible (also see: *Response requirements* in *Materials and Methods*). Such implausible response spaces are marked as cells with a black x and were not considered in the main results. Results without such constraints are depicted in S4 Fig. Results show that the change of MSEs, particularly for low MSE values, is smooth, suggesting that parameter space has been well sampled in all ROIs.

5. I think it’s a bit unfair on Alink et al to say on page 21 that “The influence of noise on the utilized forward models has been demonstrated by Ramírez and Merriam [24] and could thus explain the difference in results as well”, since in Alink et al’s reply to Ramírez and Merriam, we did explore a wide range of noise levels and still found that *only local scaling could capture the qualitative*

*patterns in our Experiment 1*. However, I admit that I do not have a ready explanation why the present authors found that other models could explain their data properties qualitatively, since those qualitative patterns were identical to those in our Experiment 1. Thus while I doubt this discrepancy owes to noise levels (or density/range of grid search for that matter), I accept it could owe to the *more fine-grained feature space* (or cropping of non-circular spaces, or even different statistical criteria for “fitting”?).

- We revisited the corresponding section in the manuscript, as we certainly want to do justice to the original paper and the reply by Alink et al. We adjusted the discussion and now also make reference to the reply by Alink et al. to the comment by Ramírez and Merriam. The discussion now reads:

“Moreover, we estimated noise levels, using independent empirical data, instead of adding a small, fixed amount of noise. A potential influence of noise on the utilized forward models was pointed out by Ramírez and Merriam [24], although follow-up analyses by Alink et al. [25] showed that their conclusions were (for their ‘face’ dataset, which most resembles our data) robust to variations in noise levels, thus rending differences in noise modelling an unlikely explanation for the discrepancy.”

6. Do the authors have a theoretical reason for using the terms “dampening” and “gain modulation” rather than the “scaling” used by Alink et al? I appreciate that “dampening” might be more descriptive, or “gain modulation” might be more precise, but it might confuse the literature to have too many terms – could the authors at least make explicit somewhere in the paper that the terms are (or are not) equivalent?

- We agree with the reviewer that reducing the number of terms referring to similar concepts is important. Indeed, we chose to use the term ‘dampening’ and ‘gain modulation’ here as they have been previously used in studies and reviews of predictive processing (e.g. see (De Lange et al., 2018; Han et al., 2019; Press et al., 2020; Richter et al., 2018; Teufel & Fletcher, 2020). For us it was important here to use the terminology as applied in studies of predictive processing, rather than the terms used in the original modelling paper by Alink et al., to ensure that our readers familiar with the predictive processing literature can relate the terminology to previous debates. However, we do appreciate that this could lead to confusion for those more familiar with the paper by Alink et al. In the Results section, describing the summary of the forward modelling procedure, we now clarify the terminology with reference to Alink et al., by stating that:

“Gain modulations (“scaling” in Alink et al. [19]) linearly scaled the responsiveness of neurons, without modulating the shape of the response function. Tuning modulations (“sharpening” in Alink et al. [19]) narrowed the shape of the response function in feature space but did not affect the peak amplitude.“

A similar clarification was added to the Methods section, introducing the model types: “Gain modulation models reduced the response of a neuron in a linear fashion – in Alink et al., referred to as “scaling” models [19]” and “On the other hand, tuning models modulated responses by reducing the width of the response functions – in Alink et al., referred to as “sharpening” models [19]”.

7. When introducing the new, seventh data pattern of “IP” on page 7, a brief rationale could be added, e.g., summarising what is stated in Supplementary Material on page 34.

- We now briefly describe the rationale of “IP” on page 7:
“IP indexes the amplitude modulation by image preference rank and constitutes an addition to the set of outcome metrics assessed by Alink et al. [19]. The rationale of IP is to assess the magnitude of expectation suppression as a function of responsiveness within rather than across voxels. Positive IP slopes indicate that neural suppression increases the more responsive a neural population is to a given stimulus.”

8. In Fig S1, why do the x-axis labels in Panel A and B have different ordering of models (and colours)?

- We thank the reviewer for noticing this discrepancy. We have adjusted the x-axis labels in Fig S1A.

9. Line 1293: “assed” = “assessed”
- Adjusted the spelling mistake.

Reviewer 2

Comments to the Author
Richter et al. explore the mechanisms underlying expectation suppression, a consistently reduced neural response to expected compared to unexpected stimuli. This down-regulation has been explained by two alternative accounts: first, expectations can filter out neural responses redundant with high-level predictions (“dampening” model). Second, expectations could suppress neural responses tuned away from the expected stimulus (“sharpening” model). However, previous studies inferred either dampened or sharpened responses at the neural level from voxel-level activity. Here, the authors set out to extend a forward modelling approach recently used to model a similar effect (adaptation suppression) to investigate expectation suppression across seven (uni- and multivariate) fMRI outcomes within three regions of interests in the ventral visual stream. Their modelling results are in line with a dampening account of expectations.

I was pleased to read this paper, which tries to answer a crucial question in a timely debate. Overall, I think the study is well-motivated, and its contribution should be of interest to a wide range of researchers. Moreover, the methods and the analytical approach are solid. I further appreciate that the authors fully share the data and analysis scripts employed. Thus, my overall impression is very positive. I only have a couple of points for clarification.

- I am primarily concerned by the fact that the data in this paper come from studies that originally showed a dampened response. Although the authors briefly mention this in the discussion, I wonder to what extent it limits the conclusions that can be drawn from the modelling approach, since as the authors describe, the specifics of the task and the stimuli frequently lead to either sharpening or dampening of responses. Thus, I would recommend toning down the conclusions and highlighting that the results apply to this experimental context in particular, and not necessarily to all instances of expectation suppression.

- The reviewer is correct that we have previously shown support for a dampened response in one of the two analysed datasets (Richter et al., 2018), using the image preference (IP) analysis, which was also used as one of the outcome metrics in the present manuscript. However, the motivation of the present study is to go beyond such voxel level analyses as relationships between neural modulations and voxel level results can be counter-intuitive, as demonstrated by Alink et al. (2018). In other words, it cannot be taken for granted that voxel level metrics necessarily map onto neural modulations as we may intuitively expect. Thus, here we question and scrutinise the inferences we previously made on the voxel level by using the forward model approach by Alink et al.

That said, we do agree with the reviewer that task, stimulus choice, and the type of statistical learning in the utilized datasets are important to consider and could have an impact on the observed results. Accordingly, we have expanded the discussion section

dealing with the limitations of the present study to better reflect these concerns. “**Limitations**
The present results must be interpreted with some limitations in mind. First, while we synthesized empirical fMRI data from two separate studies, resulting in a large dataset (n = 56) including separate tasks, it is crucial to consider the type of perceptual expectations investigated in these datasets. Both studies probed visual expectations extracted incidentally from statistical regularities. Thus, it remains unclear whether the perceptual expectations studied here, following incidental statistical learning, involve similar neural mechanisms and consequences as explicitly learned expectations. It is possible that different routes towards the acquisition of statistical regularities exist, relying on different neural mechanisms [38], thus raising the question whether the resulting sensory consequences may also differ. Relatedly, both datasets involved full-color object images as stimuli. While studies using various kinds of stimuli have provided support for the dampening account [14–18] it remains unclear whether specific stimulus types may result in distinct neural modulations, as at least one study in support of sharpening utilized oriented gratings [9]. In sum, at present it is unknown whether specific task requirements, learning or stimulus types could induce different prediction-based neural modulations.”

- Regarding the analysis plotted in Figure 2, I wonder if it would be clearer to compute partial correlations between the three models and the neural data. That way, you could test whether the relevant feature space model significantly explain a unique portion of variance, while controlling for the variance explained by the other two models.

- We agree with the reviewer that displaying the unique contribution of each feature space is more informative. Accordingly, figure 2 now displays partial correlation coefficients. The corresponding results text, as well as supplemental tables have been adjusted. In brief, results are qualitatively unchanged.

The results section now reads:
“In brief, for each participant we correlated the feature space (model) representational dissimilarity matrix (RDM) with the neural RDM, obtained from an fMRI object localizer run, while controlling for the influence of the other model feature spaces using partial correlation. Subsequently, we compared the obtained correlation coefficients against zero (i.e., no correlation between feature space and neural RDM). RSA results, depicted in Figure 2, showed that the feature spaces reliably explained unique neural variance in their target ROIs (V1, orientation feature space: *t*(55) = 6.43, p = 3.3e-8, *d*z = 0.86; LOC, shape feature space: *t*(55) = 5.06, p = 5.1e-6, *d*z = 0.68; TOFC, semantic feature space: *t*(55) = 3.27, p = 0.002, *d*z = 0.44).”

**Figure 2: Stimulus feature space models explain neural variance in target ROIs.** Shown are RSA results, in terms of Fisher z-transformed Spearman’s Rho (ρ), per ROI (V1, LOC, TOFC) and feature space model (blue = orientation feature space, green = shape complexity feature space, yellow = semantic similarity feature space). In V1 only the orientation feature space (blue) explained significant neural variance. In LOC all three models explained some neural variance, however numerically the shape complexity model (green) outperformed both other feature space models. In TOFC the semantic similarity and shape complexity feature space models explained significant neural variance, however the semantic similarity model explained numerically the most variance of neural responses. Thus, the designed feature spaces reliably captured neural variance in their target ROIs, validating the usefulness of the feature space models. Error bars indicate the SEM. * p < 0.05. ** p < 0.01. *** p < 0.001.

Editor

Comments to the Author:

Dear Dr Richter,
Thank you for submitting this interesting manuscript. As you will see, both reviewers found your manuscript of interest in principle, but also raise a number of concerns that would be important to address in a revision.

In addition to the comments made by the reviewers, I would like to add a few points:

1. As noted by Reviewer 1, it would be good to show the final fits of the winning model in comparison to the data in an overview figure. This would help readers to take away the key message.

- We agree that showing the fit of the winning model is a good addition to the manuscript. For details please refer to our reply to R1’s comment 1.

2. I would recommend to use less acronyms. Please revisit your acronyms throughout the paper and try to reduce them as much as possible.

- We have reduced the number of acronyms by stating the full name of each outcome metric throughout the manuscript text.

3. Please provide an overview of the number of parameters per model in the main text

- When providing a summary of the modelling procedure in the results section of the main text (p. 10), we now clarify that:
“The entire procedure was repeated for each model type across a large parameter grid (n = 7,820; for details see: *Materials and Methods, Simulation, Parameter grid*), extensively exploring the three free model parameters: *a* (suppression magnitude), *b* (effect of distance in feature space; only applicable to local and remove models), and σ (width of neural response function).”

4. Please use the same axis scaling in Figure 5, such that readers can appreciate the differences in the overall level of fits

- We agree that explicitly recognizing the differences in fits across ROIs is useful. The figure below depicts the results shown in Figure 5 on the same axis scaling, illustrating the difference in fits between ROIs.

However, using the same axis scaling across ROIs makes interpreting the within ROI differences between model types visually more challenging. We believe that differences between model types is more important for the conclusions of the present study than fits across ROIs. Hence, we think it is preferrable to keep Figure 5 with different axis scaling to make visual interpretation of the results simpler for our readers. However, we do believe that explicitly highlighting the different overall fits between ROIs in the main text is a valuable addition. Thus, the results section now contains this addition:

“In particular, local tuning and global gain modulation models showed numerically worse fits, but these were not reliably (statistically) different across multiple parameterizations from local gain modulations, probably due to the notably larger variance in model fits in LOC compared to V1 – results were qualitatively similar in TOFC. Indeed, the model fit was overall better in V1 than in the higher visual ROIs.“

5. Following Reviewer 1’s suggestion, I would carefully word the possible implications to the comparison of the suppression parameters to animal studies.

- We agree that our previous conclusion with respect to the parameter value comparison to results from electrophysiology was not sufficiently convincing to warrant inclusion in the

main text. Thus, we moved the associated results and discussion to the supplemental material and toned down the associated conclusion. For details please see our reply to R1’s comment 2.

Reviewer 1- first revision. ACCEPT

The authors have done a good job revising their paper, and I have no further major points.

For the record, I did download their code and run parts of it, because I wanted to check whether their models could still fit qualitatively when the BC slope is negative, like in the two datasets of Alink et al (rather than positive, as in the authors’ datasets). This might help explain why Alink et al only found one model (for their Experiment 1 at least) was consistent with the qualitative pattern of significant results in their data, whereas the present authors found multiple models. Unless I am mistaken, simply by reversing the sign of “mri_metrics_slopes(3,:)”, I found that none of the authors’ models could fit the qualitative results for their “LO” or “TO” ROIs, and though four models could satisfy p<.05 (or p<.01 to match Alink) for their “V1” ROI, this was for a tiny fraction of the parameter space, and so could still be false positives (given the large number of tests performed; determining the correct significance threshold being a weakness of this qualitative approach).

I realise that this “qualitative” fitting based on p-values is far from satisfactory (and their quantitative MSE fits are superior), but I still think it suggests that the BC data pattern may be critical for comparing their results with those of Alink et al. While it remains a puzzle why none of their models seems able to explain negative BC values (which local scaling could in Alink et al) – which may reflect their superior modelling of the stimulus space – and while the authors do acknowledge the different pattern of empirical BC results across studies in their Discussion – perhaps they could say a bit more about the importance of BC, eg in relation to other possible differences in statements like “This difference to Alink et al. [19] could be due to a finer and broader grid of parameters that was searched, using noise levels estimated from empirical data, and a more fine grained simulation of stimuli throughout the modeled feature-space (see Discussion).”

Reviewer 2 – first revision. ACCEPT

The authors have addressed all of my points. I have no further comments.

Decision letter

| **From:** | sam.gilbert@ucl.ac.uk |  |
| --- | --- | --- |
| **To:** | david.richter@donders.ru.nl |  |
| **CC:** |  |  |
| **BCC:** | sam.gilbert@ucl.ac.uk |  |
| **Subject:** | Oxford Open Neuroscience - Decision on Manuscript ID OXFNSC-2021-002.R1 |  |
| **Body:** | 27-Apr-2022   Dear Dr. Richter,   Manuscript ID OXFNSC-2021-002.R1 entitled "Dampened sensory representations for expected input across the ventral visual stream" which you submitted to the Oxford Open Neuroscience, has been reviewed.  The comments of the reviewer(s) are included at the bottom of this letter.   Thanks for this very responsive revision. We are returning the manuscript to you with a decision of 'minor revision' so that you have the opportunity to respond to the comment made by the second reviewer if you wish. Once we receive your resubmission we will endeavour to deal with it swiftly without further review.   Please note that this journal operates with transparent peer review. This means that if your submission is accepted for publication, the full peer review history of your article will publish online alongside your article. This includes reviewer comments, editor decision letters, and your author responses.   To revise your manuscript, log into https://mc.manuscriptcentral.com/oxfnsc and enter your Author Centre, where you will find your manuscript title listed under "Manuscripts with Decisions."  Under "Actions," click on "Create a Revision."  Your manuscript number has been appended to denote a revision.   You may also click the below link to start the revision process (or continue the process if you have already started your revision) for your manuscript. If you use the below link you will not be required to login to ScholarOne Manuscripts.   *** PLEASE NOTE: This is a two-step process. After clicking on the link, you will be directed to a webpage to confirm. ***   https://mc.manuscriptcentral.com/oxfnsc?URL_MASK=28e9ca3992ac46988c0d741c55332028    You will be unable to make your revisions on the originally submitted version of the manuscript.  Instead, revise your manuscript using a word processing program and save it on your computer.  Please also highlight the changes to your manuscript within the document by using the track changes mode in MS Word or by using bold or colored text.   Once the revised manuscript is prepared, you can upload it and submit it through your Author Centre.   When submitting your revised manuscript, you will be able to respond to the comments made by the reviewer(s) in the space provided.  You can use this space to document any changes you make to the original manuscript.  In order to expedite the processing of the revised manuscript, please be as specific as possible in your response to the reviewer(s).   IMPORTANT:  Your original files are available to you when you upload your revised manuscript.  Please delete any redundant files before completing the submission.   Because we are trying to facilitate timely publication of manuscripts submitted to the Oxford Open Neuroscience, your revised manuscript should be uploaded as soon as possible.  If it is not possible for you to submit your revision in a reasonable amount of time, we may have to consider your paper as a new submission.   Once again, thank you for submitting your manuscript to the Oxford Open Neuroscience and I look forward to receiving your revision.   Sincerely,  Dr. Sam Gilbert  Senior Editor, Oxford Open Neuroscience  sam.gilbert@ucl.ac.uk    Reviewer: 2   Comments to the Author  The authors have addressed all of my points. I have no further comments.   Reviewer: 1   Comments to the Author  The authors have done a good job revising their paper, and I have no further major points.   For the record, I did download their code and run parts of it, because I wanted to check whether their models could still fit qualitatively when the BC slope is negative, like in the two datasets of Alink et al (rather than positive, as in the authors’ datasets). This might help explain why Alink et al only found one model (for their Experiment 1 at least) was consistent with the qualitative pattern of significant results in their data, whereas the present authors found multiple models. Unless I am mistaken, simply by reversing the sign of “mri_metrics_slopes(3,:)”, I found that none of the authors’ models could fit the qualitative results for their “LO” or “TO” ROIs, and though four models could satisfy p<.05 (or p<.01 to match Alink) for their “V1” ROI, this was for a tiny fraction of the parameter space, and so could still be false positives (given the large number of tests performed; determining the correct significance threshold being a weakness of this qualitative approach).   I realise that this “qualitative” fitting based on p-values is far from satisfactory (and their quantitative MSE fits are superior), but I still think it suggests that the BC data pattern may be critical for comparing their results with those of Alink et al. While it remains a puzzle why none of their models seems able to explain negative BC values (which local scaling could in Alink et al) – which may reflect their superior modelling of the stimulus space – and while the authors do acknowledge the different pattern of empirical BC results across studies in their Discussion – perhaps they could say a bit more about the importance of BC, eg in relation to other possible differences in statements like “This difference to Alink et al. [19] could be due to a finer and broader grid of parameters that was searched, using noise levels estimated from empirical data, and a more fine grained simulation of stimuli throughout the modeled feature-space (see Discussion).”   Associate Editor  Comments to the Author:  Thanks for thoroughly revising the paper. As you can see, both reviewers found the revision satisfactory and are happy with the paper as it is. Note, however, that based on his/her own investigations of the code, R2 raises a possible issue when negative BC slides are considered. If the authors can verify this issue, I believe it warrants at least discussion. | |
| **Date Sent:** | 27-Apr-2022 |  |

Author response second revision 2

We are delighted that the adjustments to our manuscript adequately addressed the reviewers’ and editor’s previous comments. Below we address R1’s remaining comment.

We thank R1 for pointing out the discrepancy between our forward models and the ones used by Alink et al. [19] in fitting their empirical fMRI data pattern. Specifically, none of our forward models (in LO and TO) could fit the empirical results of Alink et al. [19] – that is, a negative slope for the between-class correlation (BC) metric, compared to our positive BC slopes. We agree with the reviewer that the difference in empirical fMRI metrics is important to highlight, as it likely explains differences in the qualitative model fitting results between the two studies. We now stress the relevance of the BC slope in the corresponding section of our Discussion: “First, it should be noted that our empirical fMRI results differ qualitatively from those presented by Alink et al. [19], particularly with an opposite sign of the slope of the between-class correlation metric. This difference in empirical data may relate to the different phenomena being studied, repetition suppression [19] compared to expectation suppression. It is possible that our pattern of fMRI results, especially the positive slope of the between-class correlation metric, is more readily fit by several model types” (lines 441-446).

In addition, we investigated multiple factors that may explain the discrepancy in qualitatively fitting the empirical data from Alink et al. [19]. First, we confirm R1’s observation that our forward models, as reported in the manuscript, only fit the empirical outcomes reported in Alink et al.’s face dataset for very few parameter combinations in V1 and not at all in LO and TO. We then performed several control simulations, as detailed below (see: Control analyses), to assess possible contributions of various factors and their combination. The factors we explored are: (1) modelling noise as a free parameter, (2) modelling stimuli as two distinct classes, (3) adjustments to the BC metric. To foreshadow and summarize the results, several factors, related to the paradigm (BC metric calculation and stimulus modelling), as well as noise modelling, do in combination appear to explain the differences between our forward models, those reported in Alink et al. [19], and the ability of our models to fit their qualitative results (negative BC slope). While most of these factors were already discussed in our manuscript to some degree, we now modified the Discussion section to better reflect these insights. However, we do believe that the control analyses do not warrant inclusion in the manuscript, as they are beyond the main aim of the paper (i.e., modelling expectation suppression, not comparing forward modelling approaches), and are limited in scope; i.e., do not systematically investigate the influence of each factor across a large parameter grid, but rather serve to support R1’s and our previous interpretation that these factors are potential candidates to account for the differences in model fits.

The relevant part of the Discussion section now states: “In addition, adjustments to the outcome metrics, particularly the correlation metrics, necessitated by the different fMRI paradigms (for details see: Materials and Methods: fMRI outcome metrics), may have allowed for more readily qualitative fits of the models. Another possible explanation for the additional flexibility of the models is that we modelled stimuli across the entire feature space, instead of only two specific feature values [19], hence resulting in a more dispersed and varied distribution of the stimuli across feature space. Moreover, we estimated noise levels, using independent empirical data, instead of adding a small, fixed amount of noise. A potential influence of noise on the utilized forward models was pointed out by Ramírez and Merriam [24], although follow-up analyses by Alink et al. [25] showed that their conclusions were (for their ‘face’ dataset, which most resembles our data) robust to variations in noise levels. However, the analysis by Alink et al. [25] also demonstrated that not all noise levels (specifically, signal-to-noise ratios) resulted in the same overall fits, thus rending differences in noise modelling a possible explanation for the discrepancy. Finally, a substantially finer resolution and broader scope of the explored parameter grid may have contributed to additional model flexibility as well, even though we also enforced additional response constraints based on plausible neural responses (see: Materials and Methods, Simulation, Response requirements)” (lines 446-461).

Additionally, the Materials and Methods section now explains the changes to the WC/BC metric calculation in more detail: “The within-class and between-class correlation metrics were modified compared to their use in Alink et al. [19] to account for challenges specific to the study of expectation suppression and the fMRI paradigm used in the empirical data acquisition. Specifically, due to the sluggishness of the BOLD response, the obtained data represented the combined response to leading and trailing images (each stimulus presented for 500ms without ISI). Given that each expected trailing image was always preceded by the same leading, while unexpected trailing images could be preceded by various leading images, the average correlation would be highly biased (larger) in favor of the expected condition. We accounted for this by calculating the correlation metrics between an expectation free localizer run (i.e., presentation of the trailing images in isolation, without leading image) and the average pattern elicited by the stimuli during the main task split into the two expectation conditions” (lines 745-755).

Control analyses

The following sections describe the control analyses referenced above in more detail. Each control simulation was identical to the main forward models, except for modifying one or more factors of interest, only considering the 6 outcome metrics (at p<0.01) as used in Alink et al. [19], and exploring a smaller grid of n=700 or n=900 parameter combinations, depending on the analysis (limited in size due to computational costs for performing multiple control analyses). Additionally, for all control analyses the empirical fMRI BC outcome metric slope was artificially inverted to qualitatively match the empirical fMRI metrics from Alink et al.’s face dataset (i.e., a negative BC slope).

First, we performed a control analysis modelling noise as a free parameter. This control analysis was motived by differences in how noise levels were set in Alink et al. [19] and estimated from empirical data in our model. Moreover, the relevance of modelling noise has previously been highlighted by Ramírez and Merriam [24] in a comment on the original paper by Alink et al. [19]. That said, in their reply Alink et al. [25] showed that their conclusions remained unchanged even when noise was modelled as a free parameter, demonstrating the robustness of their results. Nonetheless, Figure 1 of the reply by Alink et al. [25] to Ramírez and Merriam [24], demonstrates that the proportion of parameter combinations and indeed whether the model fit the face dataset at all, did depend on the SNR and hence also on the noise parameter value. Since our forward models did not include noise levels as a free parameter, but instead estimated a suitable noise parameter from the empirical fMRI data (see: Materials and Methods: Simulation: Noise estimation) it is plausible that differences in noise levels can account for discrepancies in qualitative model fitting. Our control analysis that included noise as a free parameter, showed that multiple model types could now qualitatively fit the empirical results with an inverted BC slope. In fact, in LO and TO all model types could fit the six outcome metrics, with local and global gain modulations (“scaling” in Alink et al. [19]) showing the most robust fits of all model types (4-5% of parameter combinations). Similarly, in V1 global and local gain modulations fit all outcome metrics with 6-7% of parameter combinations, while global and remote tuning failed to fit all metrics. Thus, the inclusion of noise level as a free parameter contributed additional flexibility to our models to qualitatively fit the empirical results of Alink et al. [19] and hence constitutes one factor which may explain why our forward models showed different characteristics in qualitatively fitting the empirical outcome metric slopes and particularly the negative BC slope. However, we believe that estimating appropriate noise levels from the empirical fMRI data is superior to including noise as a free parameter in our model, as the goal of the forward modelling approach is to explain empirical fMRI data.

The next factor we explored concerns modelling stimuli across the whole continuous feature space, as done here, compared to constraining stimuli to two distinct classes, as in Alink et al. [19]. Repeating our analysis after shifting all stimuli into two classes resulted in no qualitative fits of our forward models to the combined six outcome metrics. Thus, these results suggest that when assessed in isolation, modelling stimuli across the feature space did not account for the loss of flexibility for our models in fitting the negative BC slope. It is possible that results would have differed if a larger parameter gird would have been explored in the control analysis (n=700 parameter combinations compared to n=7820 in the main analysis), however given the computational cost, this was not possible for the various control analyses reported here.

Given the relevance of noise parameter levels, we also explored the combination of including noise as a free parameter and modelling stimuli as two distinct categories of a feature space. Results showed that in V1 only local gain modulations could fit the empirical fMRI results with negative BC slopes (3% of parameter combinations), echoing conclusions of Alink et al. [19]. In LO and TO local gain modulations most reliably fit the empirical data (~1% of parameter combinations), but also local tuning and remote gain modulations fit for some parameter combinations (most <0.5%). Thus, the combination of modelling noise as a free parameter and collapsing our stimulus set to two categories within the feature space increased the resemblance of our results to Alink et al. [19] (i.e., mostly local gain modulation (scaling) fit the negative BC slopes). Hence both factors combined may play a role in explaining discrepancies between our main forward modelling results and those reported by Alink et al. [19].

Next, due to the nature of the experimental design of the underlying MRI dataset (expectation suppression compared to repetition suppression), we adjusted the method for how the within-class correlation (WC) and between-class correlation (BC) metrics were calculated. In brief, our measured BOLD response consists of the mix of the neural responses to leading and (un)expected trailing image (stimulus presentation 500ms without ISI; for details see: Materials and Methods: Empirical fMRI data). Because leading images predicted trailing images in our design, an expected stimulus was always preceded by the same leading image, while any other leading image could precede the unexpected occurrence of that stimulus. Hence, the neural representation of the unexpected images varies significantly more across trials due to the BOLD response reflecting the mix of various leading images (on different trials) with the unexpected trailing image. We accounted for this bias by calculating the WC and BC metrics with reference to an independent, expectation-free localizer dataset, during which trailing images were presented one at a time without leading images in a blocked design, thereby yielding an unbiased and clearer representation of the trailing image. To assess the relevance of this adjustment, we performed a control analysis in which we calculated the BC metrics more similar to the original Alink et al. [19] paper within the main task, rather than with reference to a localizer dataset, and without normalizing the parameter estimates. Note: this adjustment does not introduce bias in the forward models, as the temporal dynamics of the BOLD response were not modelled. However, for the main analysis it is still advisable to use the same analysis procedure as for the empirical fMRI data, because otherwise empirical and modelled data would be analyzed with different procedures. Results showed that a high proportion of the parameter space now qualitatively fit the empirical fMRI data reported by Alink et al.; specifically, 23% (V1), 12% (LO) and 0.7% (TO) for local gain modulations (scaling). Other model types now also fit all slopes with global gain modulations being particular successful in fitting the empirical data: 22% (V1), 43% (LO) and 6% (TO). Other model types only fit the empirical outcomes on a small proportion of parameter combinations <1% in TO and <5% in LO. Thus, adjustments to the BC metric, necessary in our main analysis to account for design specific challenges of studying expectation suppression, did significantly affect the ability of our forward models to fit the empirical fMRI data of Alink et al. [19]. Similar results, albeit with an overall smaller percentage of the parameter space fitting the empirical outcome metrics were obtained when investigating the combination of changes to the BC metric calculation and modelling stimuli as two classes.

Last, we performed a control analysis assessing the combination of the three factors explored above, thereby yielding a variation of our forward modelling approach that may be most suited to match the results reported in Alink et al. [19,25]. Thus, here we included (1) modelling noise as a free parameter, (2) modelling stimuli as two distinct classes, and (3) adjusted the BC metric. Results yielded exclusive fits of the local gain modulation model to the six empirical outcome metrics from Alink et al. [19] in V1 (3.6% of parameter space). In LO and TO, local tuning and remote gain modulation fit the empirical outcome metrics under at least one parameterization each; 0.1% and 0.3% of the parameter space respectively. However, local gain modulations fit all outcome metrics across a larger part of the parameter space (0.8%). Thus, the combination of the three explored factors appears to contribute to differences in qualitative fitting of the slopes of the outcome metrics. Accordingly, we adjusted our manuscript to reflect the importance of these factors, as noted above.

New reviewer invited for additional opinion

I think they have very thoroughly addressed the final issue that came up and explained the inconsistencies in their simulations compared to the results of Alink et al.

Final decision letter

| **From:** | sam.gilbert@ucl.ac.uk |
| --- | --- |
| **To:** | david.richter@donders.ru.nl |
| **CC:** | sam.gilbert@ucl.ac.uk |
| **BCC:** | sara.yannytillar@oup.com |
| **Subject:** | Oxford Open Neuroscience - Decision on Manuscript ID OXFNSC-2021-002.R2 |
| **Body:** | 12-Jul-2022   Dear Dr. Richter,   It is a pleasure to accept your revised manuscript entitled "Dampened sensory representations for expected input across the ventral visual stream" in its current form for publication in the Oxford Open Neuroscience.     Please note that this journal operates with transparent peer review. This means that  the full peer review history of your article will publish online alongside your article. This includes reviewer comments, editor decision letters, and your author responses.   Thank you for your fine contribution.  On behalf of the Editors of the Oxford Open Neuroscience, we look forward to your continued contributions to the Journal.   Sincerely,  Dr. Sam Gilbert  Senior Editor, Oxford Open Neuroscience  sam.gilbert@ucl.ac.uk     Associate Editor  Comments to the Author:  (There are no comments.) |
